# From Theory to Practice: Development and Evaluation of a Quality Improvement Curriculum for Psychiatry Residents

**DOI:** 10.1177/23821205241228200

**Published:** 2024-01-30

**Authors:** Aditya Nidumolu, David Freedman, Mark Bosma

**Affiliations:** 1 12361 Department of Psychiatry, Dalhousie University, Canada; 2 12366 Department of Psychiatry, University of Toronto, Canada

**Keywords:** Quality improvement, education, medical, psychiatry, curriculum

## Abstract

**OBJECTIVES:**

Quality improvement (QI) is a systematic approach used to analyze and address problems in healthcare. Evidence of its success has led some national regulatory bodies to require QI education in residency training programs. However, limited work to date has demonstrated how residency programs can integrate best practices in QI education to design their own curriculum. This study describes the implementation and evaluation of a new QI curriculum, grounded in a theoretical model of how QI education works, for Canadian psychiatry residents.

**METHODS:**

PGY-2 and PGY-4 psychiatry residents received a 2.5-h mixed didactic and simulation-based QI workshop as a part of the 2021-2022 academic curriculum. Their knowledge and attitudes toward QI were assessed using the QI Knowledge Application Tool Revised (QIKAT-R) and the Beliefs and Attitudes subscale of the Beliefs, Attitudes, Skills, and Confidence in QI (BASiC-QI).

**RESULTS:**

Eleven of 12 residents (92%) who completed the curriculum participated in the study. Average QIKAT-R scores improved from 4.45 to 7.00. Average BASiC-QI Beliefs and Attitudes subscale scores increased by 5.55 points. Residents reported enjoying QI and an increased desire to participate in future QI projects.

**CONCLUSION:**

This study demonstrates how a programme theory of QI education can be used to develop an effective, locally-tailored curriculum. This approach can be replicated by other educators to develop or improve QI curricula.

## Introduction

Quality improvement (QI) is a methodology commonly used to assess and improve the performance of healthcare systems.^1^ To equip physicians with the skills to support healthcare improvement efforts, regulatory bodies for residency training in the United States, Canada, and the United Kingdom now require trainees to receive training in QI.^2–4^ Despite the wealth of studies describing how QI can be taught, the theoretical foundations of what factors lead to effective QI curricula are yet to be routinely integrated into curricular development.^5^

This theory-practice gap was explored by Brown et al in their realist review of QI in medical education which developed a programme theory: a model to explain how QI educational programs lead to positive outcomes.^5^ A programme theory can be used by healthcare educators to rapidly identify which strategies in QI education will provide the greatest value. For example, a subanalysis of Brown et al’s realist review which focused exclusively on psychiatry trainees identified that situating QI projects halfway through residency was optimal because it ensured residents had (a) adequate knowledge of their health system to identify quality gaps and (b) enough time remaining in their programs to implement QI projects during longitudinal ambulatory care blocks.^6^

The present study describes the implementation and evaluation of a new psychiatry QI curriculum in a medium-sized institution. We describe how theoretical principles of effective QI education identified by Brown et al were used to develop the curriculum, and thus, help to close the theory-practice gap of education.^5^ This approach can be replicated by educators and researchers in any medical specialty to rapidly translate theoretical principles in QI education into on-the-ground advances.

## Methods

### Setting and participants

This curriculum was designed for psychiatry residents in the Dalhousie University Psychiatry Residency Program based in Halifax, Nova Scotia. Resident education on QI is mandatory per national training standards, and thus this curriculum was designed to be delivered during the academic day teaching schedule.^3^ Prior to the development of this curriculum, residents received limited exposure to QI concepts by attending quality review boards within the department. The curriculum was specifically designed to be given to second-year residents. This is because, at this level of training, residents had some understanding of the health system necessary to identify quality gaps but were early enough in residency that they could pursue longitudinal QI projects during protected research time. While intended for more junior residents, this curriculum was also offered to fourth-year residents given that they had no prior exposure to QI education in residency. Both the second and fourth-year resident cohorts received the curriculum in the 2021-2022 academic year. Within these two cohorts, no residents were excluded from the curriculum or from participating in its evaluation.

### Consent and Ethical Review

Participation in this curriculum's evaluation was voluntary and was solicited via email before the curriculum's delivery by a collaborator (DF) external to the curriculum's institution. Residents were offered $15 to complete two, 15-min surveys before and after the curriculum. The identities of those who completed the evaluation component were blinded to the authors delivering the curriculum. With a focus on program development, this study was deemed exempt from formally requiring participant consent by the Dalhousie University Office of Research Services.

### Curriculum Design

Two authors (AN and DF) reviewed Brown et al's programme theory of QI education to identify *curricular mechanisms* used in effective QI curricula.^5^ This refers to the strategies used to implement the curriculum, such as giving learners protected time for QI training or providing mentorship in QI. As depicted in Table 1, we deliberately applied each mechanism to the local institutional context. Some of the mechanisms studied by Brown et al could not be implemented due to a lack of local resources or infrastructure. For example, some QI curricula include structured experiential QI projects for learners or access to system-level quality measures that can be analyzed. These mechanisms were excluded from Table 1 and are explored in greater detail in Brown et al's review.^5^

**Table 1.** Mechanisms to support curriculum implementation.

Mechanism	Current implementation and rationale
Clear expectations of learners	Learners are expected to produce a “project proposal” by the end of the workshop. The proposal includes clear expectations such as an aims statement, quality measures, and a description of at least one plan-study-do-act cycle.
Protected time	Quality improvement (QI) teaching time is protected for all residents during the weekly academic day.
Mentorship and feedback	Residents can receive just-in-time feedback on all components of their project charter during the workshop. A supervising staff psychiatrist with an interest in education and QI is available to field questions about quality gaps and change ideas.
Curriculum leadership	The curriculum is championed by the program director of the psychiatry residency program and chair of the residency curriculum committee.
Incentives for participating	Residents interested in pursuing further work in QI can implement the project proposal, which will fulfill their residency research requirements.
Content delivery strategies	The workshop is taught using just-in-time learning through a combination of didactics, group discussion, and five breakout spaces for small groups to work on project proposals.
Education resources	Residents have access to workshop slides and their project proposals following the workshop.
Screening projects for appropriateness	Residents are taught how to select a “good” quality gap using decision matrices during the workshop. Workshop leaders elicit multiple quality gaps from trainees and help trainees choose a gap that has the potential for high clinical impact with a low workload burden. There is no formal preworkshop screening for projects.
Working in teams	Residents can work in pairs. This allows residents to receive feedback from a peer while being involved in every stage of developing a QI project proposal.

The content of the curriculum was drawn from the author expertise and national QI training objectives.^3^ Previously published QI curricula were referenced to ensure that no significant conceptual areas were missed.^7–9^ Overall, we designed a single-day 2.5-h curriculum combining didactic seminars and QI simulation (Supplement 1). Trainees had five separate 10- to 15-min didactic presentations with breakout sessions in between. During these presentations, they learned the definition of QI, the dimensions of quality, the components of a QI project based on the Model for Improvement,^10^ and the differences between QI and clinical research or quality assurance. In each didactic session, the specific relevance of QI to psychiatry was highlighted. For example, residents learned about the dimensions of quality by discussing specific quality problems they encounter during routine psychiatric practice. In breakout sessions, trainees worked in groups of 2 to 3 to apply newly gained knowledge to develop their own psychiatry QI project charter (Supplement 2). This proposal included the identification of a clinical quality gap, an analysis of its root causes, and a proposal for how it may be addressed using the Model for Improvement. Just-in-time feedback during each breakout session from the authors (AN and MB) helped attendees improve their proposals or consider new perspectives. At the end of the session, trainees reflected as a group, shared what they learned from the experience, and determined future directions for the implementation of the draft proposals.

### Evaluation

This curriculum was evaluated using a one-group pre/postprogram design. First, participant knowledge of QI was assessed before and after the intervention using a modified variation of the QI Knowledge Application Tool Revised (QIKAT-R). The QIKAT-R presents learners with a clinical stem of a quality gap in healthcare and asks them to generate an aim statement, measures to assess changes, and a change idea.^11^ Responses are given a maximum of three points for each of these components, for a maximum score of nine. This tool has been found to effectively discriminate between excellent, fair, and poor responses. Reardon et al developed two clinical stems relevant to psychiatry residents that can be used with the QIKAT-R.^7^ Each learner response was blinded, graded by two authors (AN and DF), and converted into a numerical score out of nine. Evaluators reconciled score discrepancies for each question through discussion.

Learners were then assessed before and after the intervention on their beliefs toward QI using the “Beliefs and Attitudes” subscale of the “*Beliefs, Attitudes, Skills, and Confidence in QI (BASiC-QI)*.”^12^ Responses were scored on a Likert scale ranging from 1 (*strongly disagree*) to 7 (*strongly agree*).

### Statistical Analysis

Aggregate pre/postscore changes for both the QIKAT-R and BASiC-QI were presented using descriptive statistics. Paired *t*-tests were used to test for significant effects with a predetermined *p-*value of < .05.

## Results

The curriculum was delivered on September 1, 2021, to 5 PGY 4 residents and on December 1, 2021, to 7 PGY 2 residents. Of these individuals, 11 out of 12 (92%) participated in the curriculum evaluation. Most participants had previously encountered QI, though only 3 (27%) had prior involvement in its development or implementation (Table 2).

**Table 2.** Prior participant experiences with quality improvement (QI).

Prior QI experience	Number of attendees (percent)
I have been involved in the development and/or implementation of a QI project.	2 (18%)
I have participated in a QI project without being directly involved in its development or implementation.	1 (9%)
I have heard/seen QI projects being developed and/or implemented in my workplace without having participated in the initiative in any capacity.	4 (36%)
None of the above apply to me.	4 (36%)

Residents proposed six unique project charters. Aims of these projects included efforts to:

1. Increase the uptake of psychiatry consult service order recommendations by inpatient teams;
2. Decrease inappropriate use of urine drug screens in the emergency department;
3. Improve the timeliness of medication and investigation orders on an inpatient unit;
4. Decrease medication interaction errors in an inpatient psychiatry unit;
5. Decrease the prevalence of constipation in an inpatient psychiatry unit;
6. Decrease the frequency of incomplete paperwork during the admission of psychiatric patients from the emergency department.

Participants completed two QIKAT-R scenarios before and after the curriculum. Participants improved on average by 2.55 out of 9 points per scenario (Table 3).

**Table 3.** Summary of outcomes.

QIKAT-R questions 1 and 2			
*Preworkshop average score (standard deviation)*	*Postworkshop average score (standard deviation)*	*Gain in score*	*p-value*
Average QIKAT-R scores for questions 1 and 2			
4.45 (2.15)	7.00 (1.07)	2.55	<.01
BASiC-QI Beliefs and Attitudes subscale summary			
47.27 (9.87)	52.82 (5.86)	5.55	<.01

QIKAT-R, Quality Improvement Knowledge Application Tool Revised; BASiC-QI, Beliefs, Attitudes, Skills, and Confidence in Quality Improvement.

Finally, participants completed the BASiC-QI subscale for belief and attitudes. Aggregate scores for the pre/postgroups demonstrated a significant difference, with an average improvement of 5.55 points after the intervention.

## Discussion

By participating in this curriculum, residents acquired greater QI knowledge and developed more positive beliefs toward QI. Through curriculum creation and evaluation, this study demonstrates an example of how theoretical principles in QI education can be applied to develop an effective curriculum, reducing the theory-practice gap. In the following discussion, findings will be contextualized by the available literature.

Knowledge outcomes of this curriculum compare favorably with other published psychiatry QI curricula. For example, a 10-month long, mixed didactic and experiential QI educational intervention for third-year psychiatry residents led to average QIKAT-R scores increases of 5.6 to 6.8 per scenario.^13^ Of note, this study also used clinical scenarios that were created specifically for psychiatry trainees.^7^ Specialty-specific scenarios may allow residents to more deeply analyze quality gaps that are relevant to their own clinical experiences. The developers of the QIKAT-R identified that “excellent” scores fell in the range of 6.7 to 8.5 on standardized scenarios.^11^ Outside of the psychiatry literature, an otolaryngology program delivered a 1-year long didactic QI curriculum based on online Institute for Healthcare Improvement modules, leading to a mean QIKAT-R score improvement of 4.64 to 7.38 per scenario.^14^ Similarly, an Emergency Medicine program implementing a 3-year, modular QI curriculum found that mean QIKAT-R scores increased from 4.3 to 6.1.^15^ In the present analysis, participant scores improved from 4.45 to 7.00 points, suggesting that they obtained a similar improvement in knowledge to previously published curricula.

Beliefs and attitudes toward QI are key outcomes of QI education to predict future interest in completing QI projects, though they are inconsistently measured in the QI literature.^11^ Participants in this curriculum gained 5.55 points on the BASiC-QI subscale. There is no standard interpretation of this magnitude of change in the literature. However, one study of a 4-month, mixed didactic and experiential QI curriculum for medical students led to an increase of 4.8 points on this subscale,^12^ comparable to the outcomes of the present curricula despite the intervention being a single half-day. Some curricula have utilized other scales for beliefs and attitudes such as the QI Confidence Instrument or Safety Attitudes Questionnaire, though no single scale is prominently used in the QI literature.^16^ Further standardization of tools to measure beliefs and attitudes may support educators in comparing the effectiveness of QI curricula.

To our knowledge, this is the first article reporting the development process of a QI educational program based on a QI programme theory. By reviewing effective curricular mechanisms identified in the literature, we were able to rapidly choose and implement QI tools realistic to our institutional context. This process may be especially relevant for educators looking to improve existing QI curricula by identifying mechanisms they may be able to use to strengthen in their existing curriculum. In this study's curriculum, all mechanisms that could realistically be implemented into a single-day curricula identified in Brown et al's review were utilized. Next steps may include examining opportunities to implement more resource-intensive mechanisms, such as training faculty mentors in QI, designating a “QI champion” to lead the curriculum, and implementing experiential QI projects.^5^

This program evaluation has several limitations to consider, the most significant of which is its smaller sample size, which is limited by the size of the psychiatry program itself. Due to the one-group quasi-experimental design of the evaluation and the small sample size, it is not possible to determine whether trainees attending this curriculum obtained strong improvements in their QIKAT and BASiC-QI scores through chance or other factors separate from the curriculum and its use of theoretical learning principles. Implementing the curriculum in multiple institutions across a greater time period would foster confidence in how this curriculum impacts participant knowledge, beliefs, and attitudes. Moreover, it is unclear whether improvements in attitude or knowledge about QI will persist over time. Evaluation of this would require follow-up assessment of participants—ideally 1 year after the intervention to be more comparable to other longitudinal curricula in the literature. Finally, the evaluation used a nonrandomized design, and therefore it is not possible to determine definitively which factors in the curriculum were most important.

## Conclusions

This study demonstrates a worked example of how theoretical principles in QI education can be used to rapidly develop QI curricula, achieving comparable outcomes to other curricula published in the literature. This approach can be used by educators in any medical specialty to develop and improve their own QI curricula. However, further work is necessary to longitudinally evaluate whether resident knowledge and interest in QI translate into increased QI involvement following residency.

## Supplemental Material

sj-pptx-1-mde-10.1177_23821205241228200 - Supplemental material for From Theory to Practice: Development and Evaluation of a Quality Improvement Curriculum for Psychiatry Residents Click here for additional data file. Supplemental material, sj-pptx-1-mde-10.1177_23821205241228200 for From Theory to Practice: Development and Evaluation of a Quality Improvement Curriculum for Psychiatry Residents by Aditya Nidumolu, David Freedman and Mark Bosma in Journal of Medical Education and Curricular Development

sj-docx-2-mde-10.1177_23821205241228200 - Supplemental material for From Theory to Practice: Development and Evaluation of a Quality Improvement Curriculum for Psychiatry Residents Click here for additional data file. Supplemental material, sj-docx-2-mde-10.1177_23821205241228200 for From Theory to Practice: Development and Evaluation of a Quality Improvement Curriculum for Psychiatry Residents by Aditya Nidumolu, David Freedman and Mark Bosma in Journal of Medical Education and Curricular Development

sj-docx-3-mde-10.1177_23821205241228200 - Supplemental material for From Theory to Practice: Development and Evaluation of a Quality Improvement Curriculum for Psychiatry Residents Click here for additional data file. Supplemental material, sj-docx-3-mde-10.1177_23821205241228200 for From Theory to Practice: Development and Evaluation of a Quality Improvement Curriculum for Psychiatry Residents by Aditya Nidumolu, David Freedman and Mark Bosma in Journal of Medical Education and Curricular Development

sj-docx-4-mde-10.1177_23821205241228200 - Supplemental material for From Theory to Practice: Development and Evaluation of a Quality Improvement Curriculum for Psychiatry Residents Click here for additional data file. Supplemental material, sj-docx-4-mde-10.1177_23821205241228200 for From Theory to Practice: Development and Evaluation of a Quality Improvement Curriculum for Psychiatry Residents by Aditya Nidumolu, David Freedman and Mark Bosma in Journal of Medical Education and Curricular Development
