# Supplementary material for: From Theory to Practice: Development and Evaluation of a Quality Improvement Curriculum for Psychiatry Residents
Source: J Med Educ Curric Dev. 2024 Jan 30;11:23821205241228200. doi: 10.1177/23821205241228200 (PMC10832440; doi:10.1177/23821205241228200)
Supplement: sj-pptx-1-mde-10.1177_23821205241228200 - Supplemental material for From Theory to Practice: Development and Evaluation of a Quality Improvement Curriculum for Psychiatry Residents [file sj-pptx-1-mde-10.1177_23821205241228200.pptx]

## Slide 1
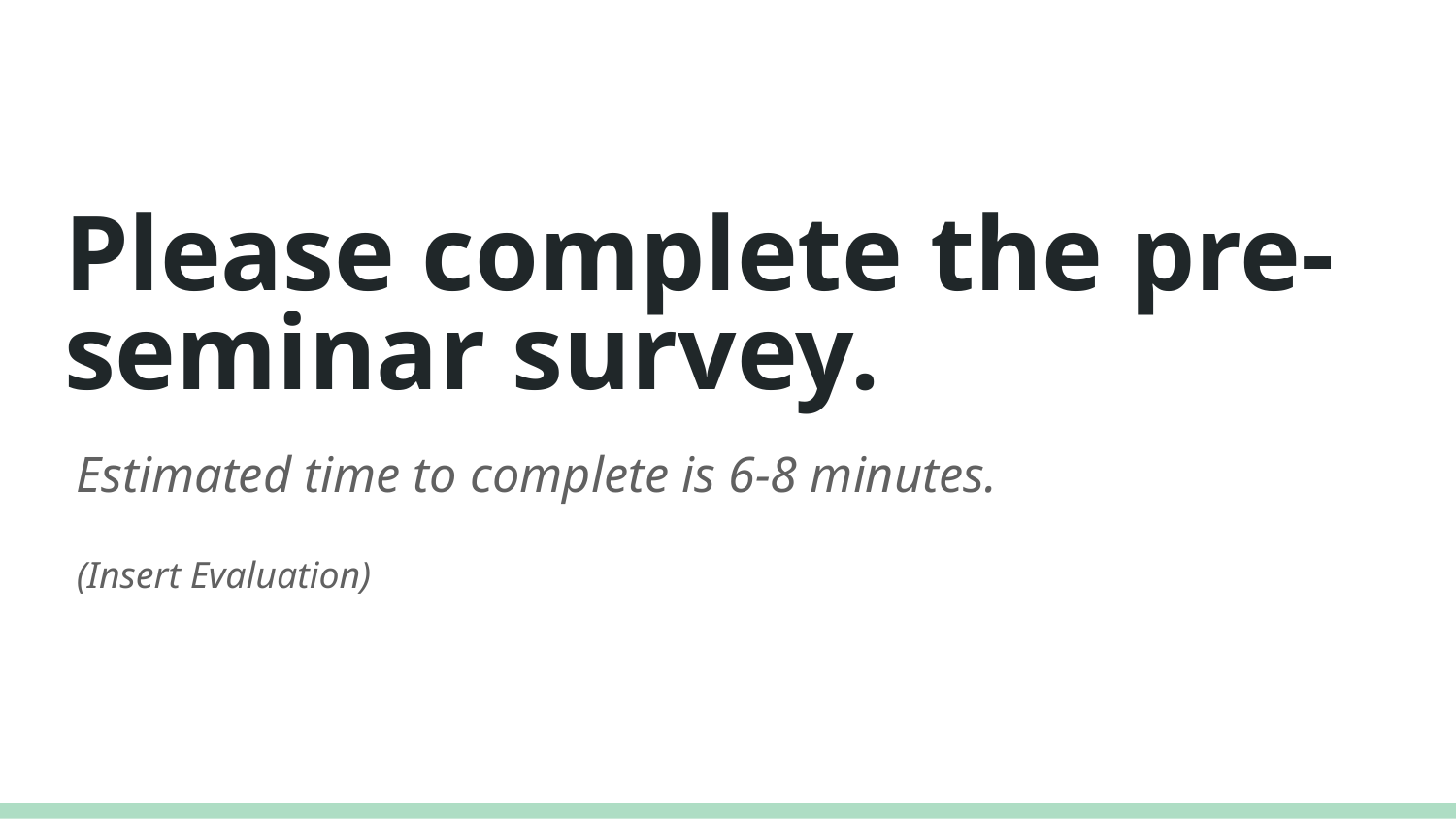

# Please complete the pre-seminar survey.
Estimated time to complete is 6-8 minutes.
(Insert Evaluation)

## Slide 2
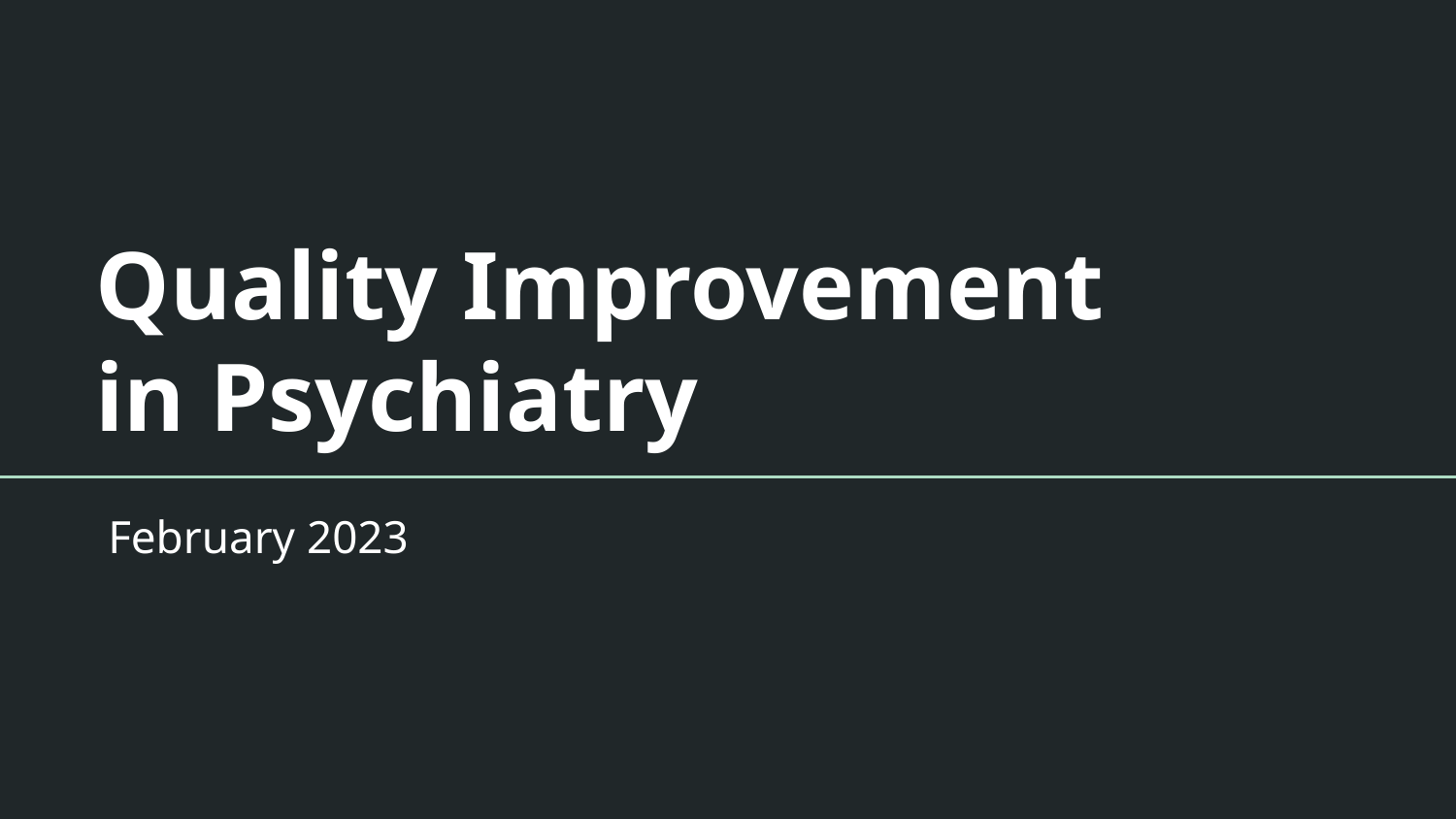

# Quality Improvement in Psychiatry
February 2023

## Slide 3
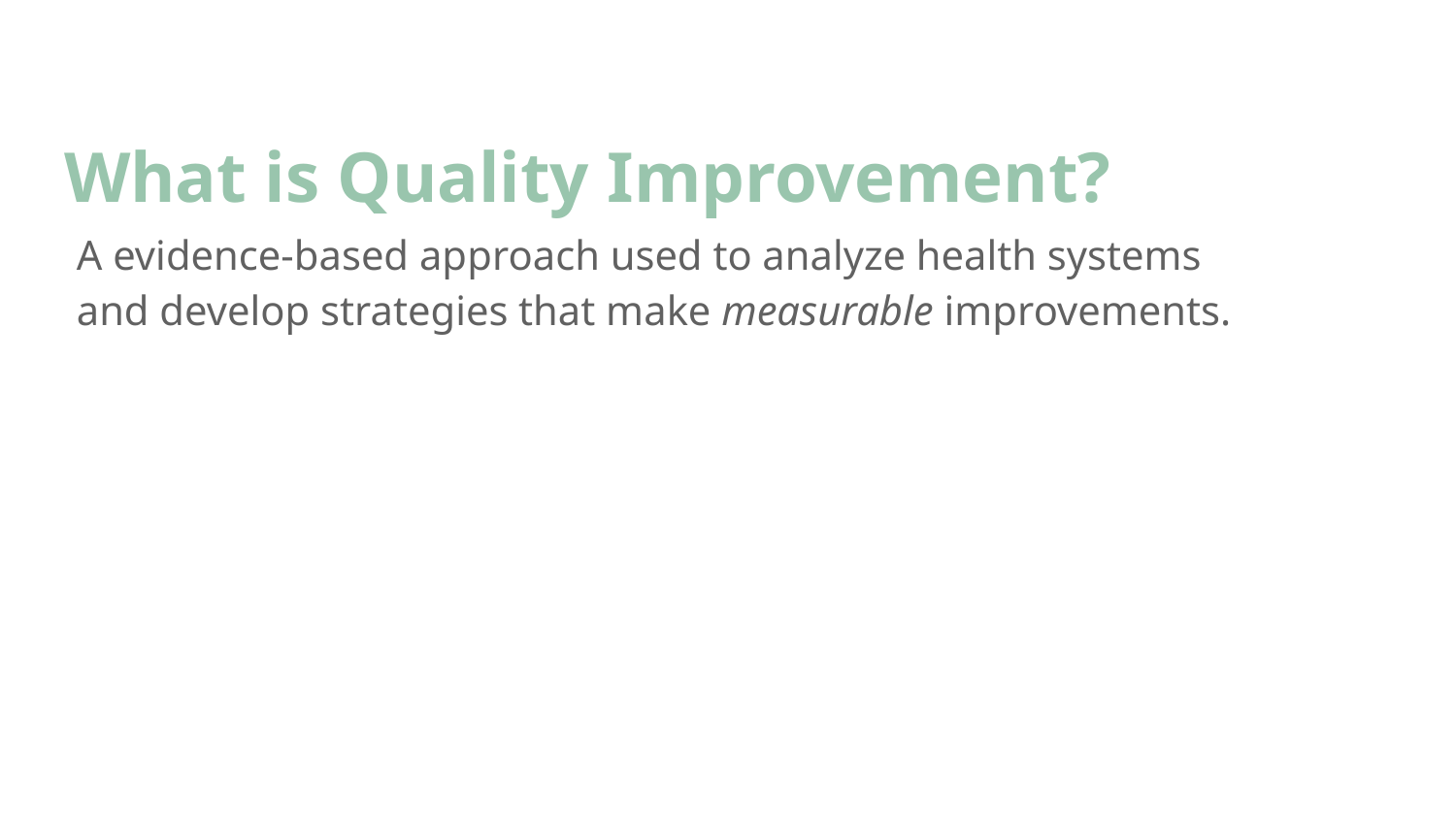

What is Quality Improvement?
A evidence-based approach used to analyze health systems and develop strategies that make measurable improvements.

## Slide 4
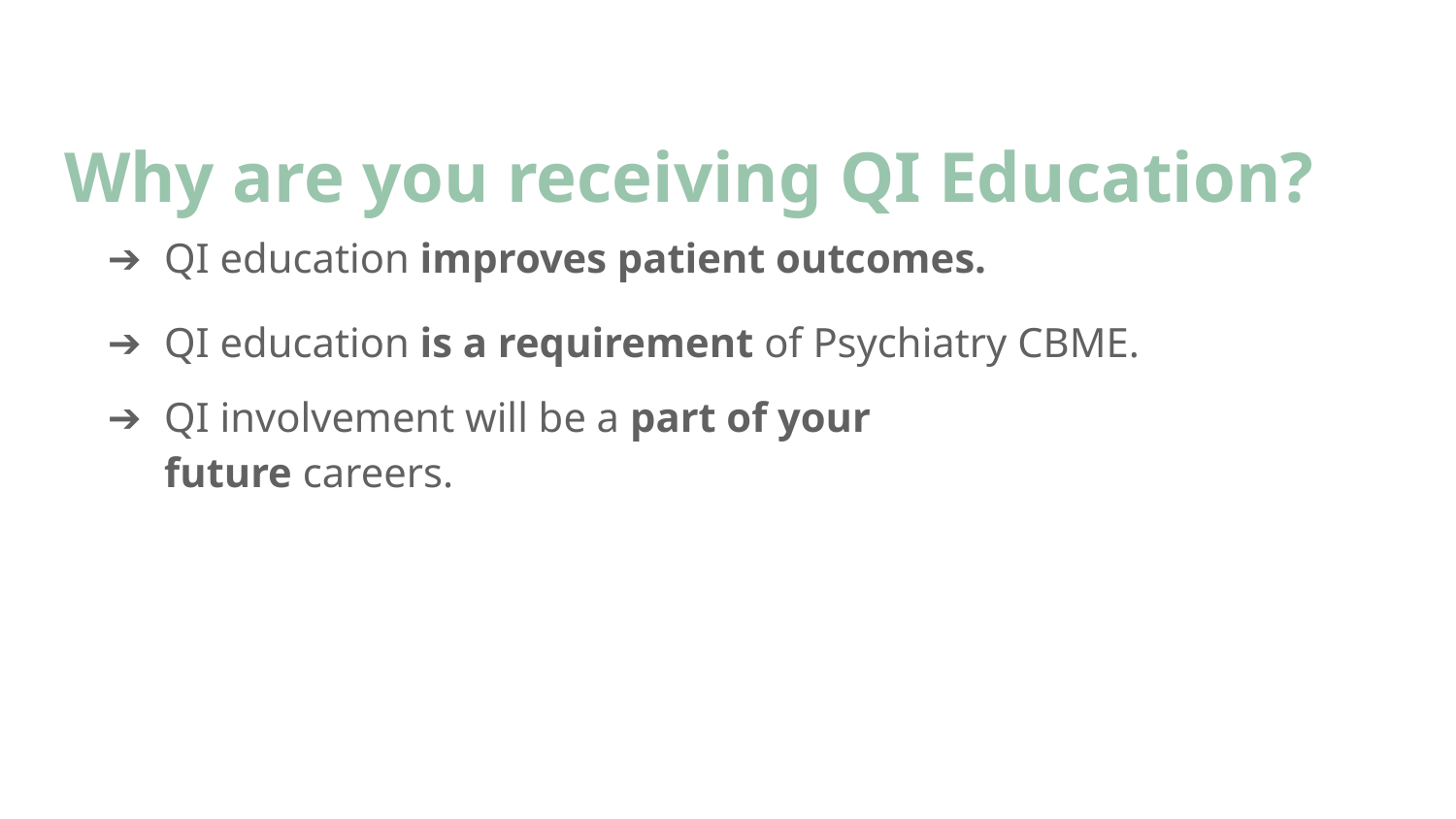

# Why are you receiving QI Education?
QI education improves patient outcomes.
QI education is a requirement of Psychiatry CBME.
QI involvement will be a part of your
future careers.

## Slide 5
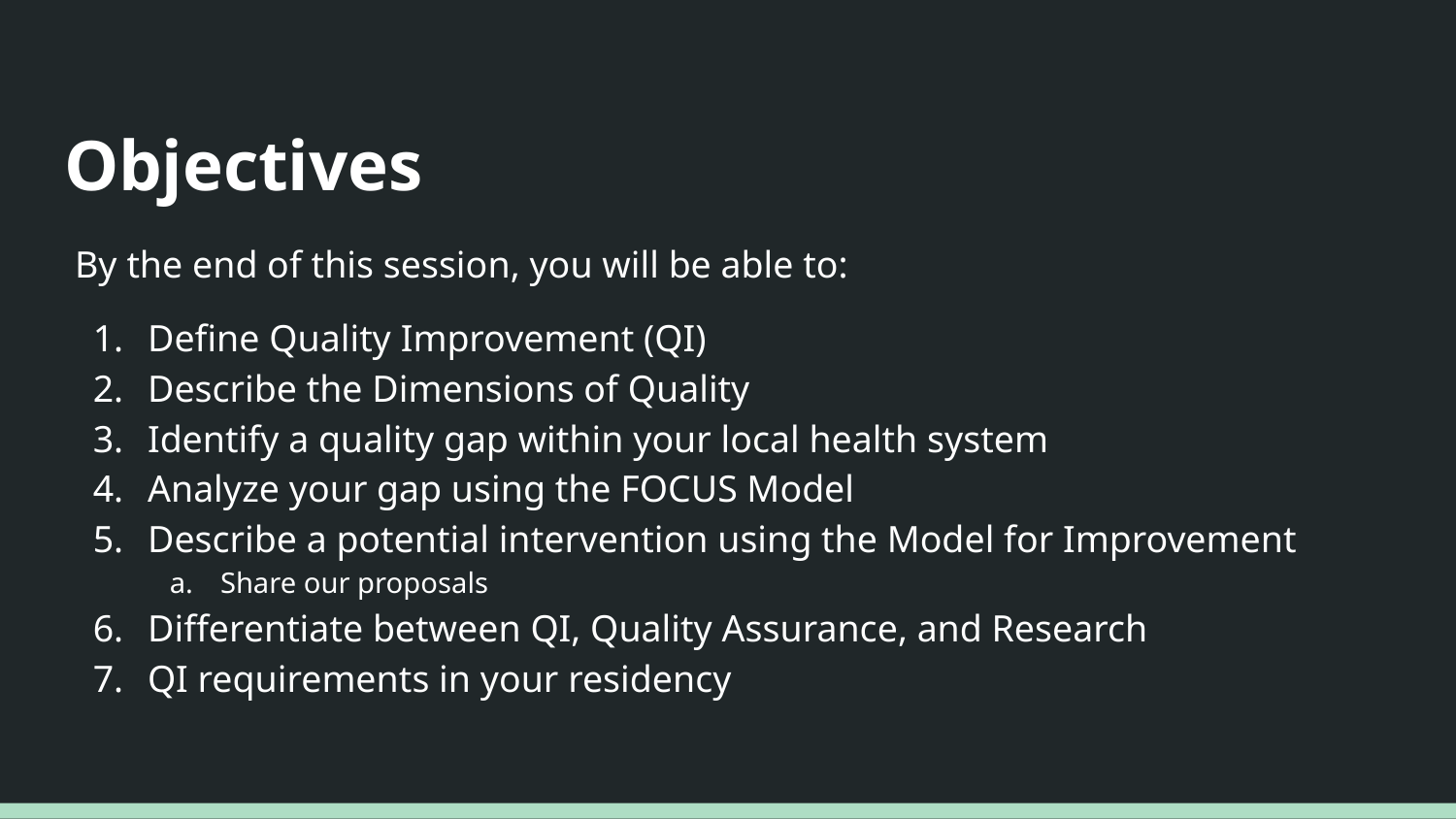

# Objectives
By the end of this session, you will be able to:
Define Quality Improvement (QI)
Describe the Dimensions of Quality
Identify a quality gap within your local health system
Analyze your gap using the FOCUS Model
Describe a potential intervention using the Model for Improvement
Share our proposals
Differentiate between QI, Quality Assurance, and Research
QI requirements in your residency

## Slide 6
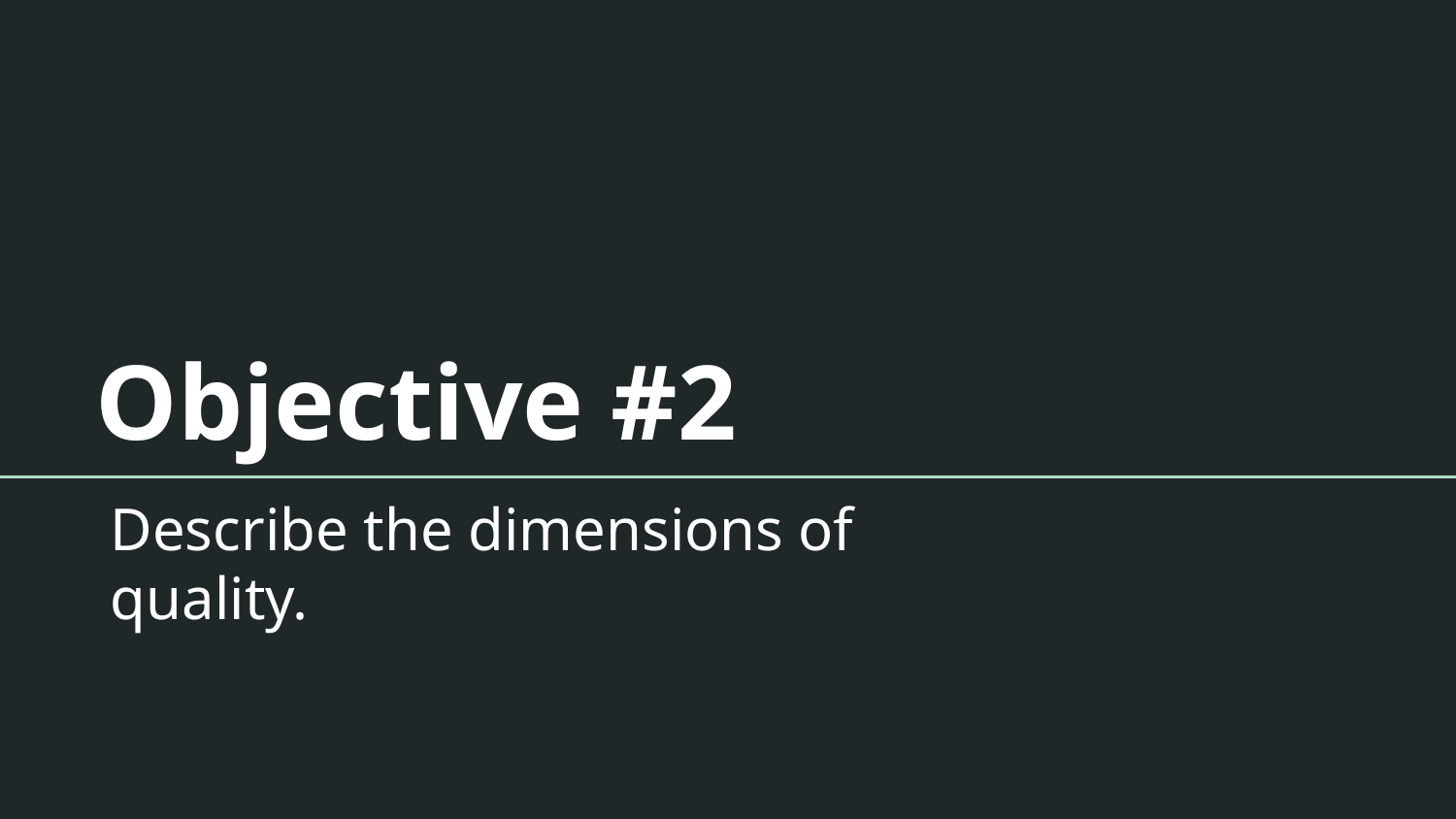

# Objective #2
Describe the dimensions of quality.

## Slide 7
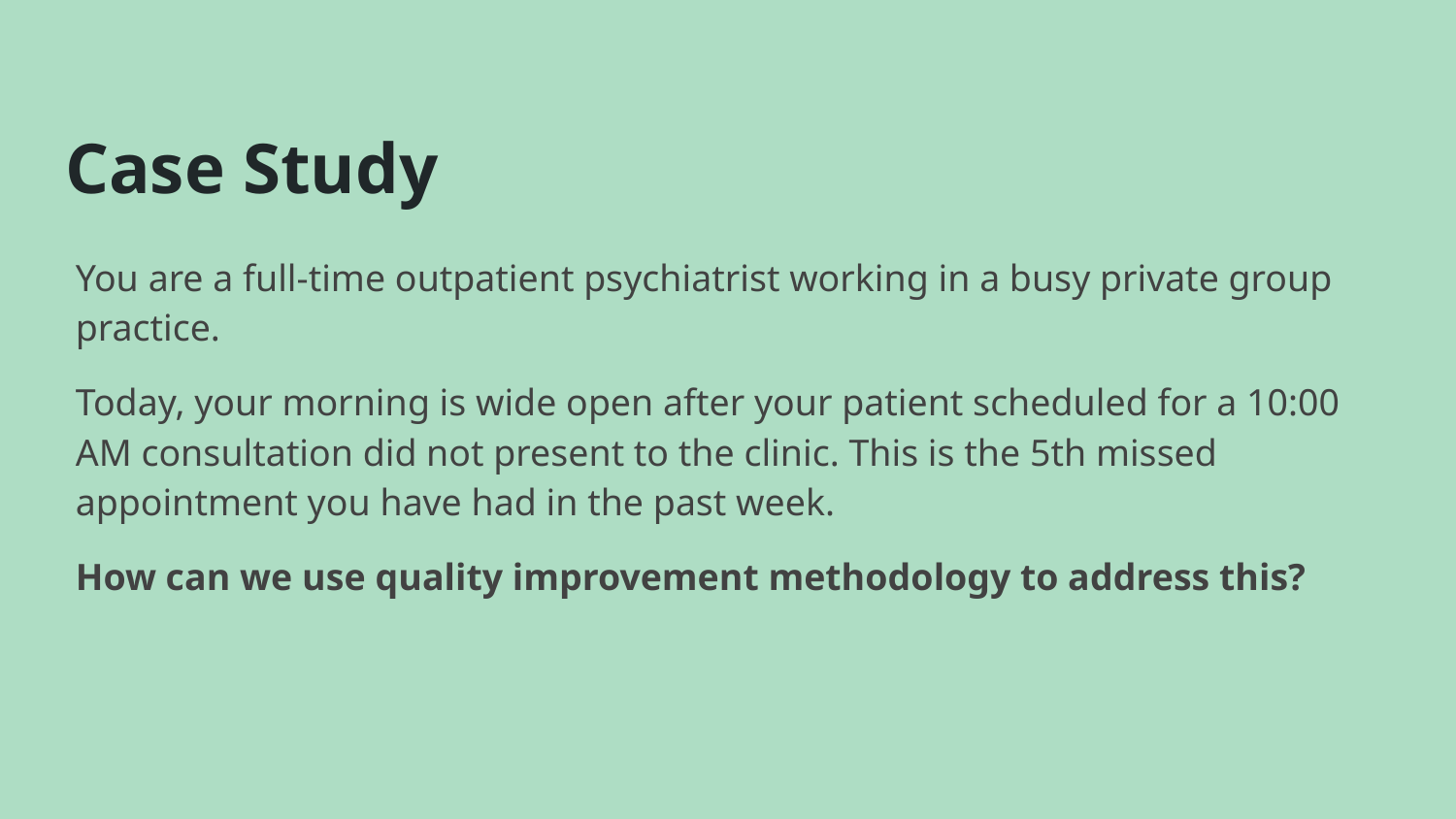

# Case Study
You are a full-time outpatient psychiatrist working in a busy private group practice.
Today, your morning is wide open after your patient scheduled for a 10:00 AM consultation did not present to the clinic. This is the 5th missed appointment you have had in the past week.
How can we use quality improvement methodology to address this?

## Slide 8
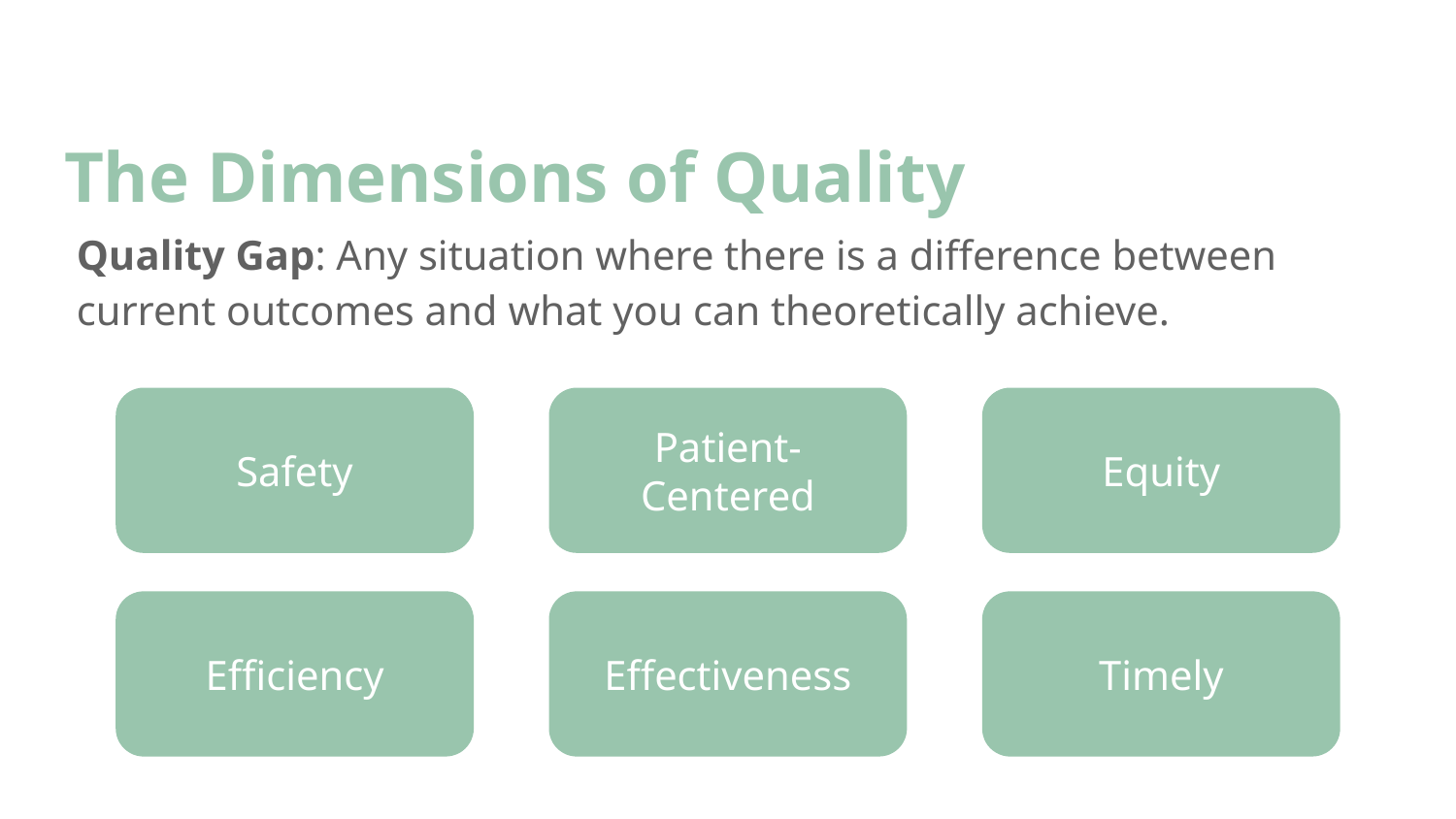

# The Dimensions of Quality
Quality Gap: Any situation where there is a difference between current outcomes and what you can theoretically achieve.
Safety
Patient-Centered
Equity
Efficiency
Effectiveness
Timely

## Slide 9
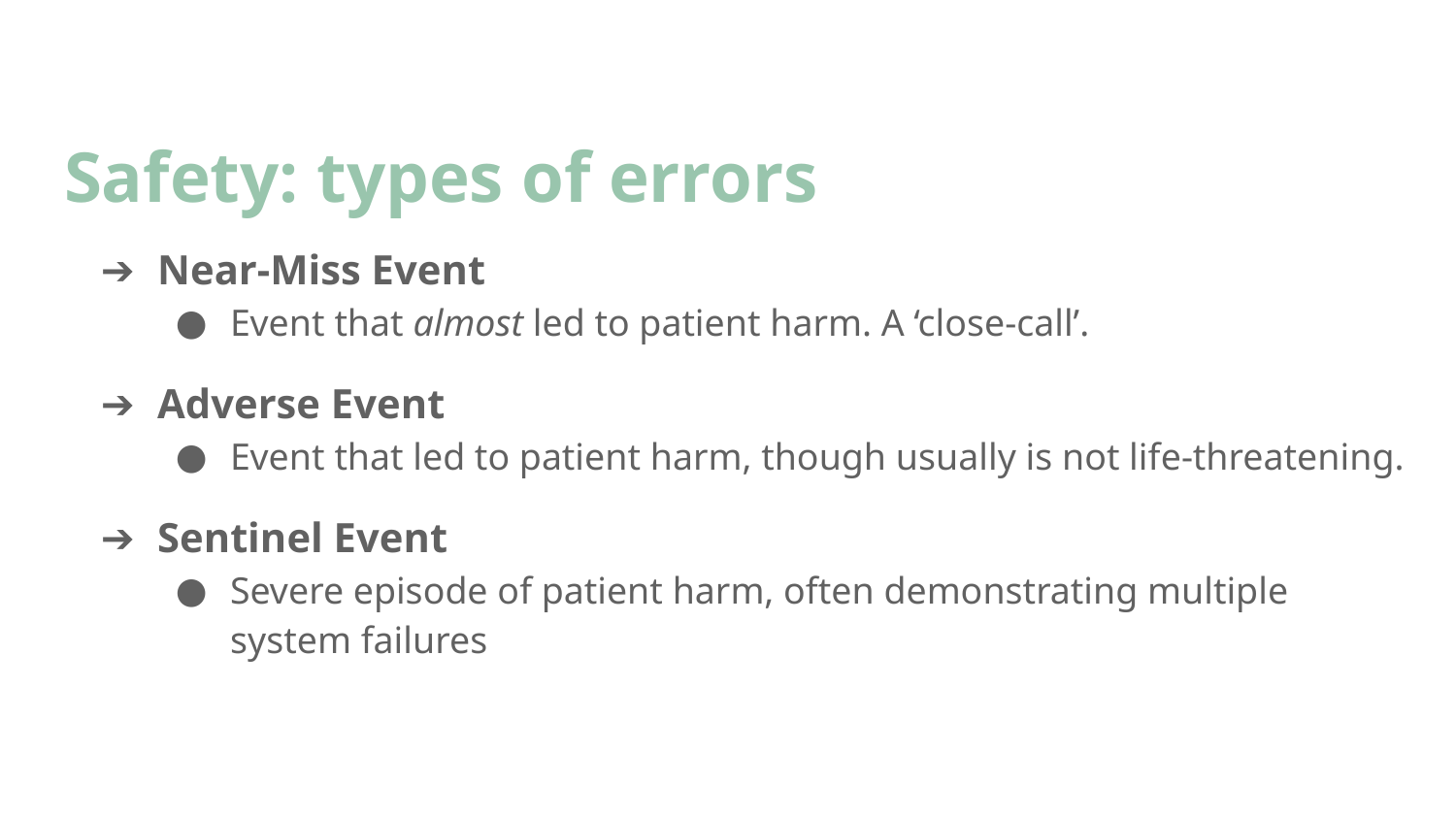

# Safety: types of errors
Near-Miss Event
Event that almost led to patient harm. A ‘close-call’.
Adverse Event
Event that led to patient harm, though usually is not life-threatening.
Sentinel Event
Severe episode of patient harm, often demonstrating multiple system failures

## Slide 10
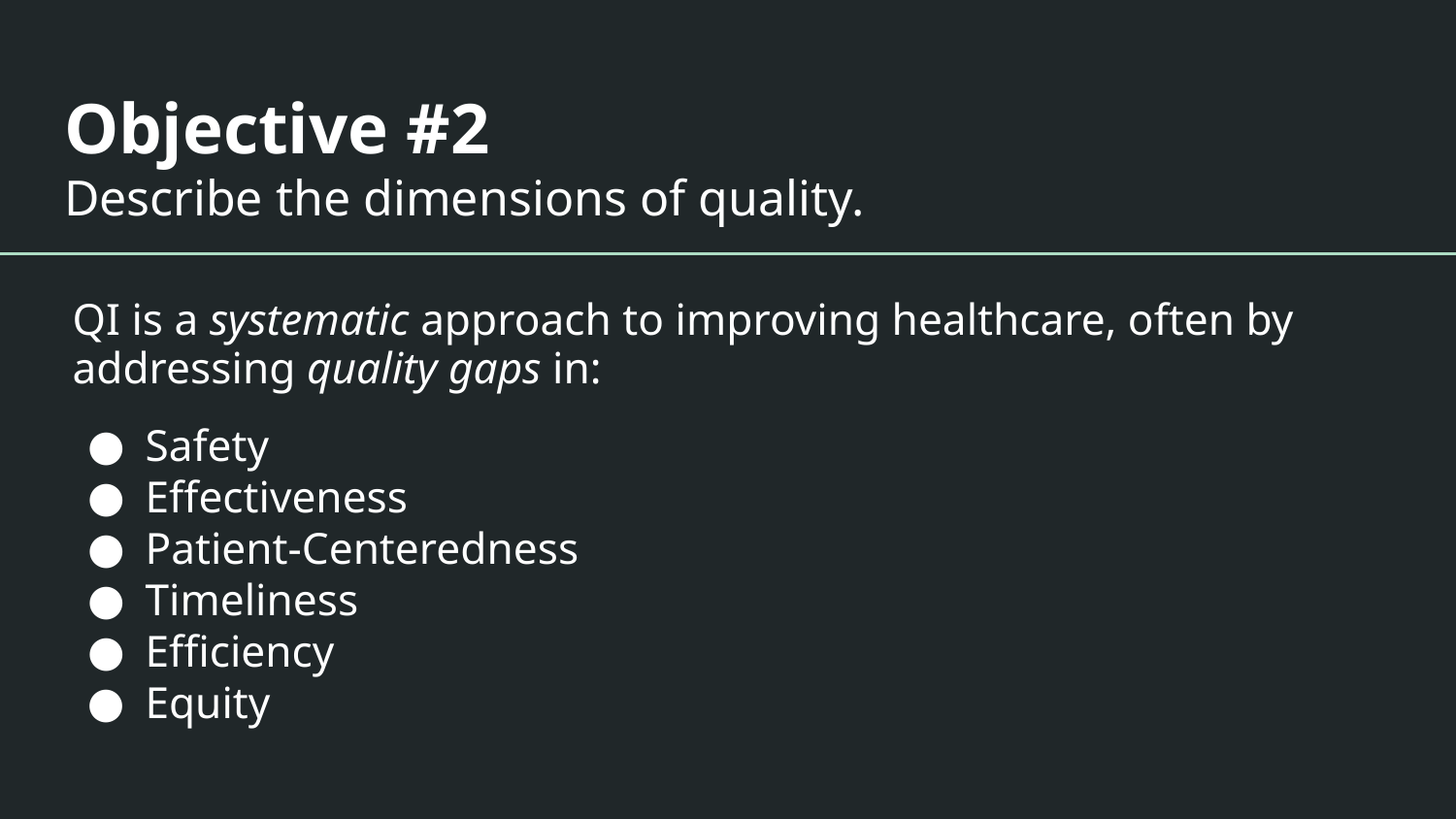

Objective #2
Describe the dimensions of quality.
QI is a systematic approach to improving healthcare, often by addressing quality gaps in:
Safety
Effectiveness
Patient-Centeredness
Timeliness
Efficiency
Equity

## Slide 11
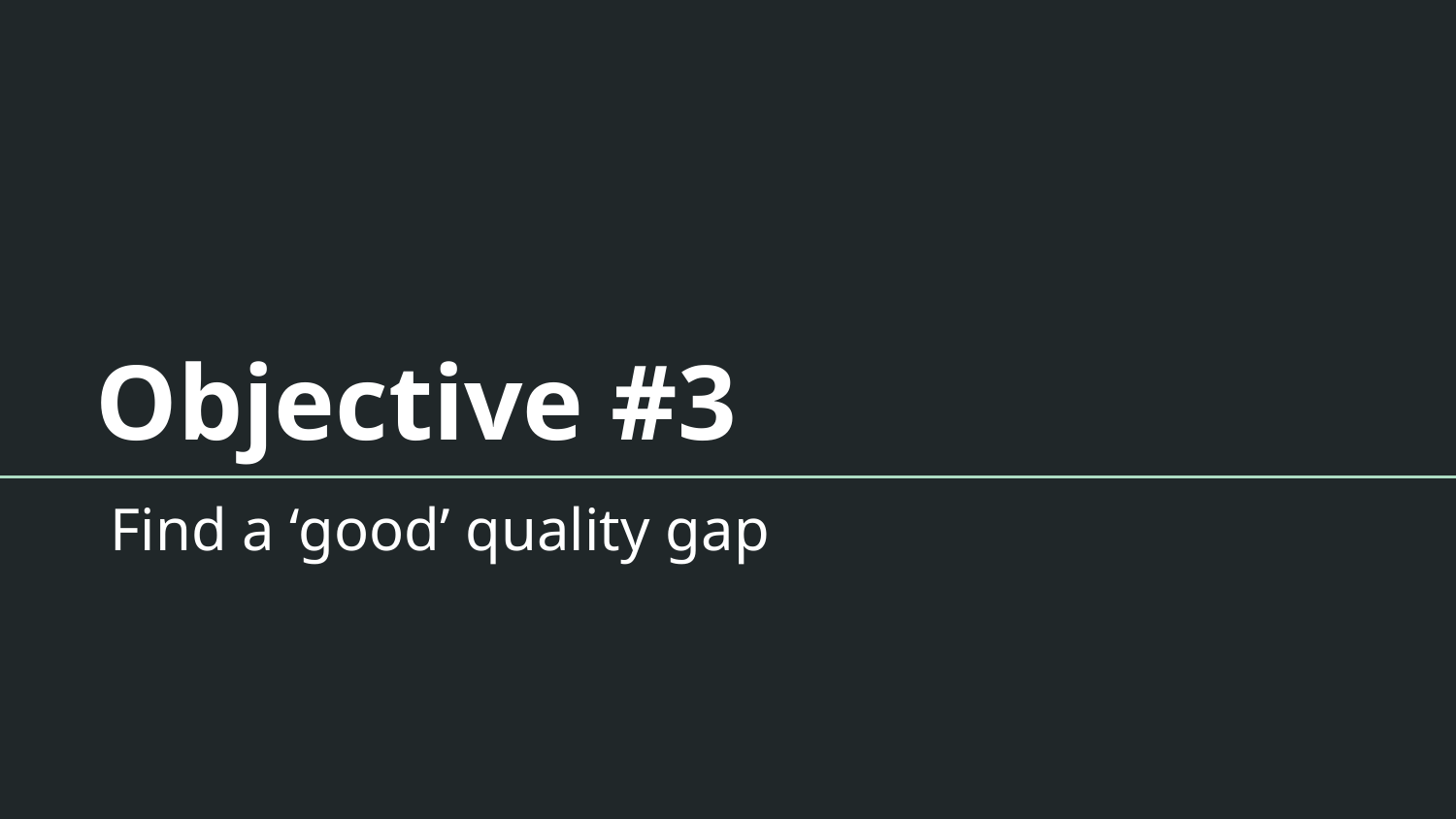

# Objective #3
Find a ‘good’ quality gap

## Slide 12
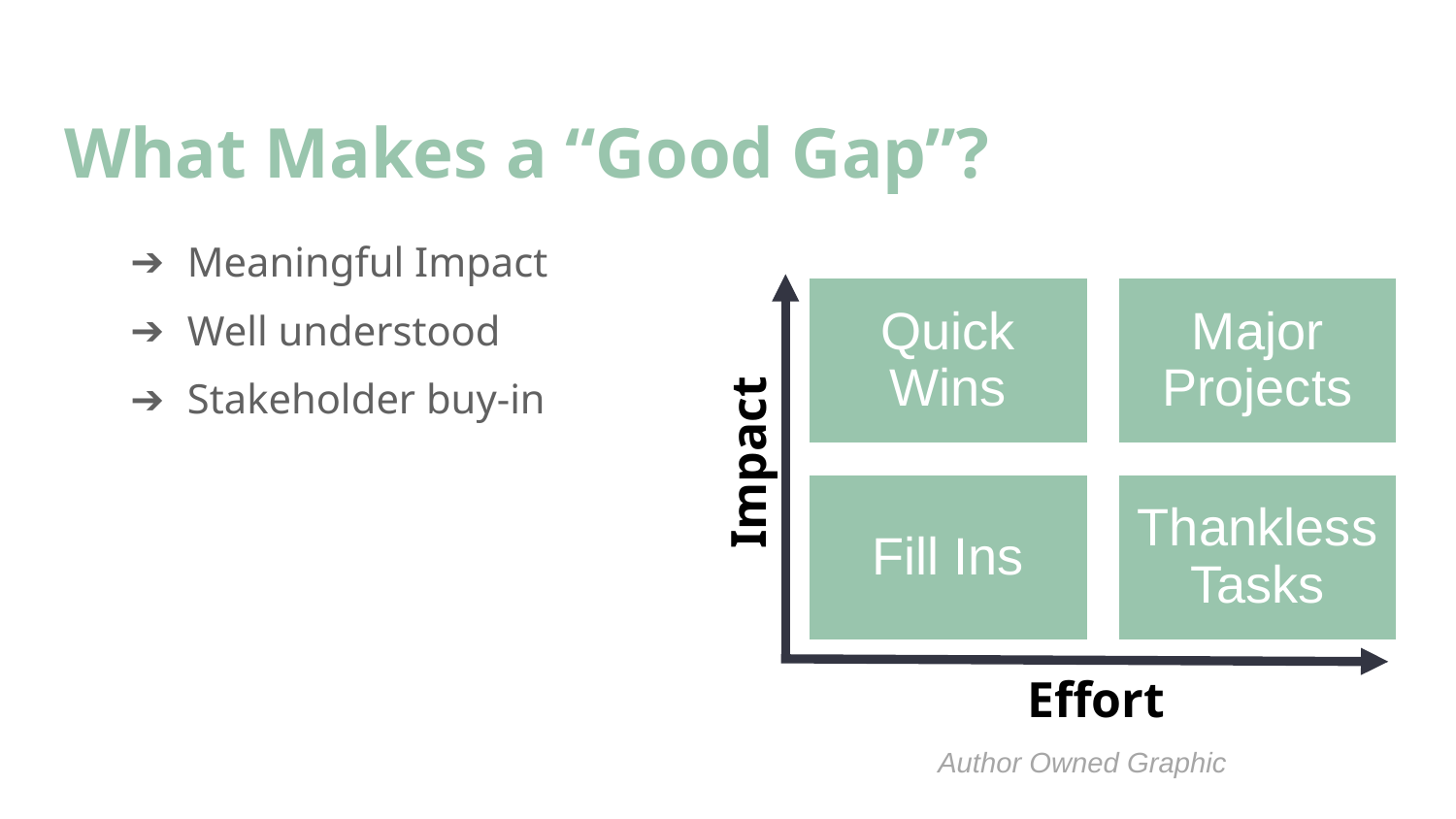

# What Makes a “Good Gap”?
Meaningful Impact
Well understood
Stakeholder buy-in
Impact
Effort
Author Owned Graphic

## Slide 13
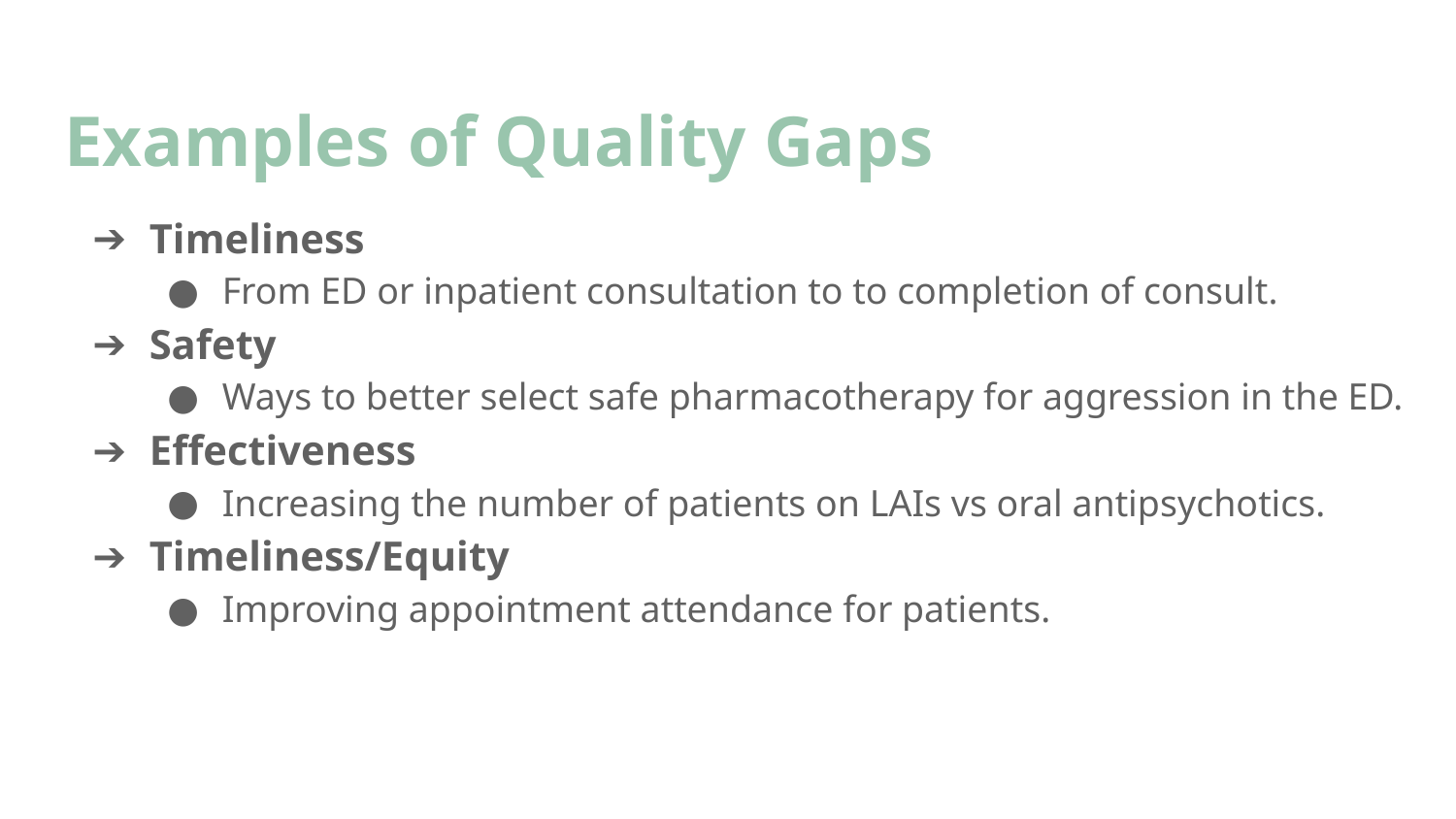

# Examples of Quality Gaps
Timeliness
From ED or inpatient consultation to to completion of consult.
Safety
Ways to better select safe pharmacotherapy for aggression in the ED.
Effectiveness
Increasing the number of patients on LAIs vs oral antipsychotics.
Timeliness/Equity
Improving appointment attendance for patients.

## Slide 14
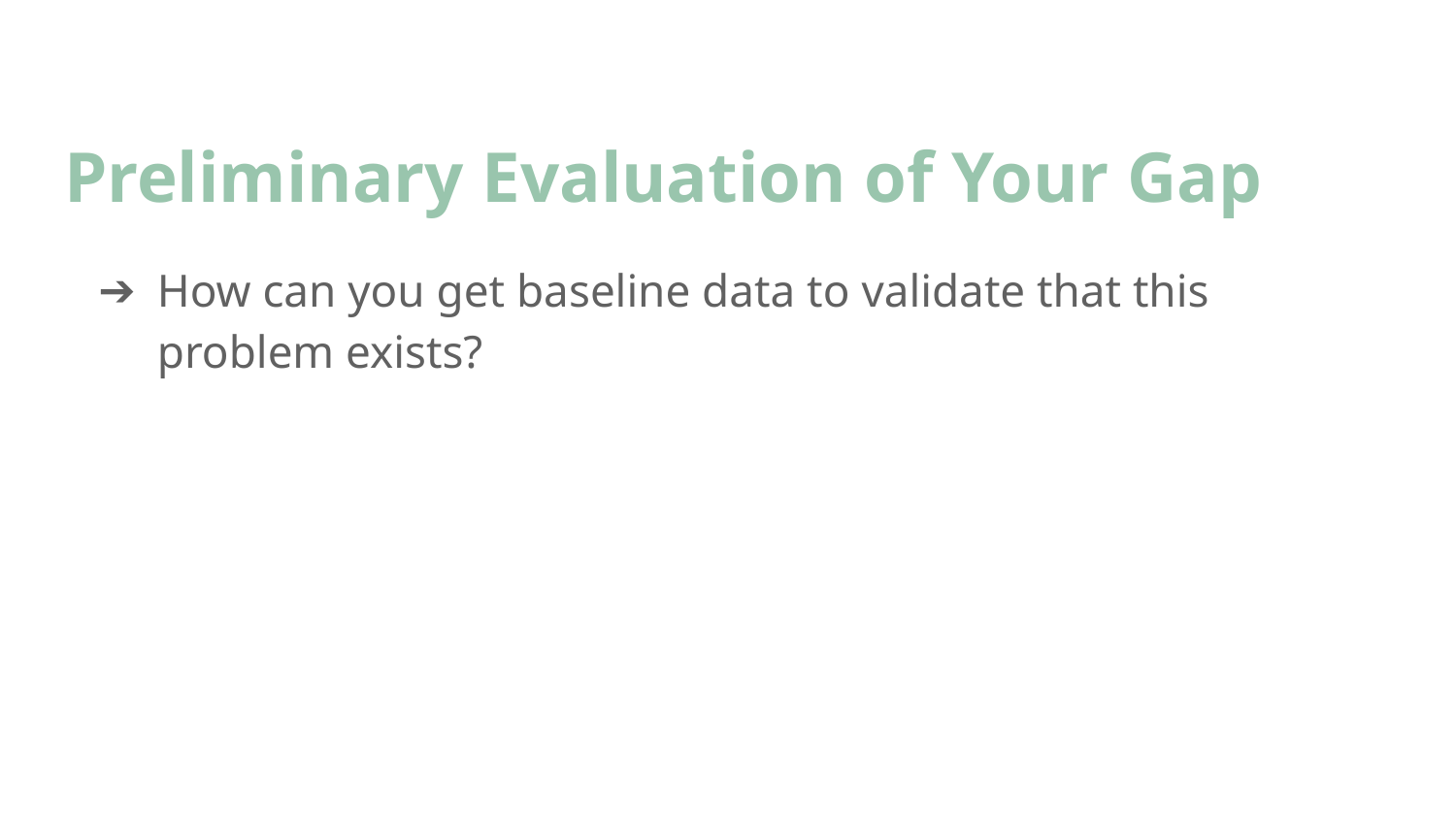

# Preliminary Evaluation of Your Gap
How can you get baseline data to validate that this
problem exists?

## Slide 15
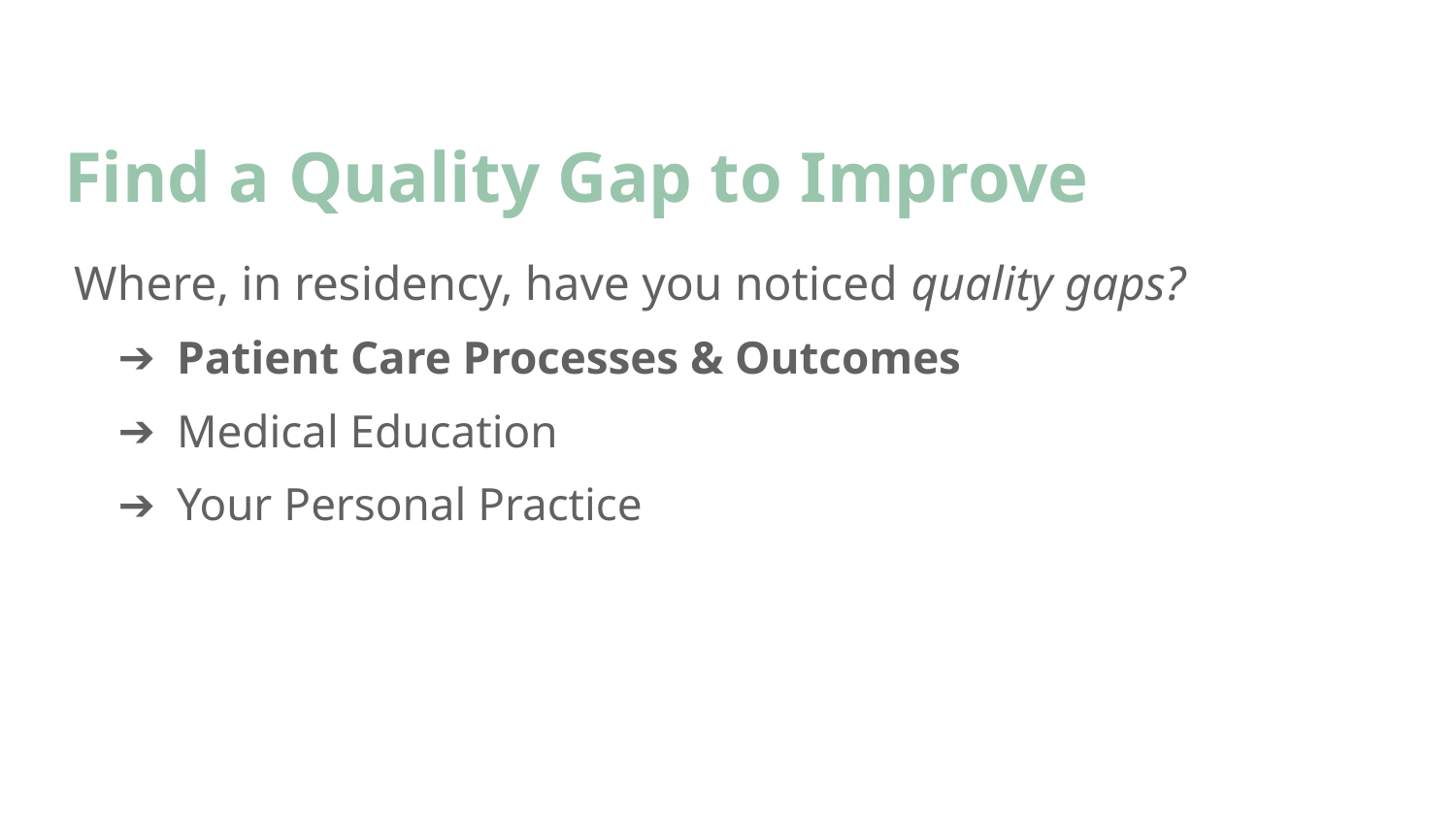

# Find a Quality Gap to Improve
Where, in residency, have you noticed quality gaps?
Patient Care Processes & Outcomes
Medical Education
Your Personal Practice

## Slide 16
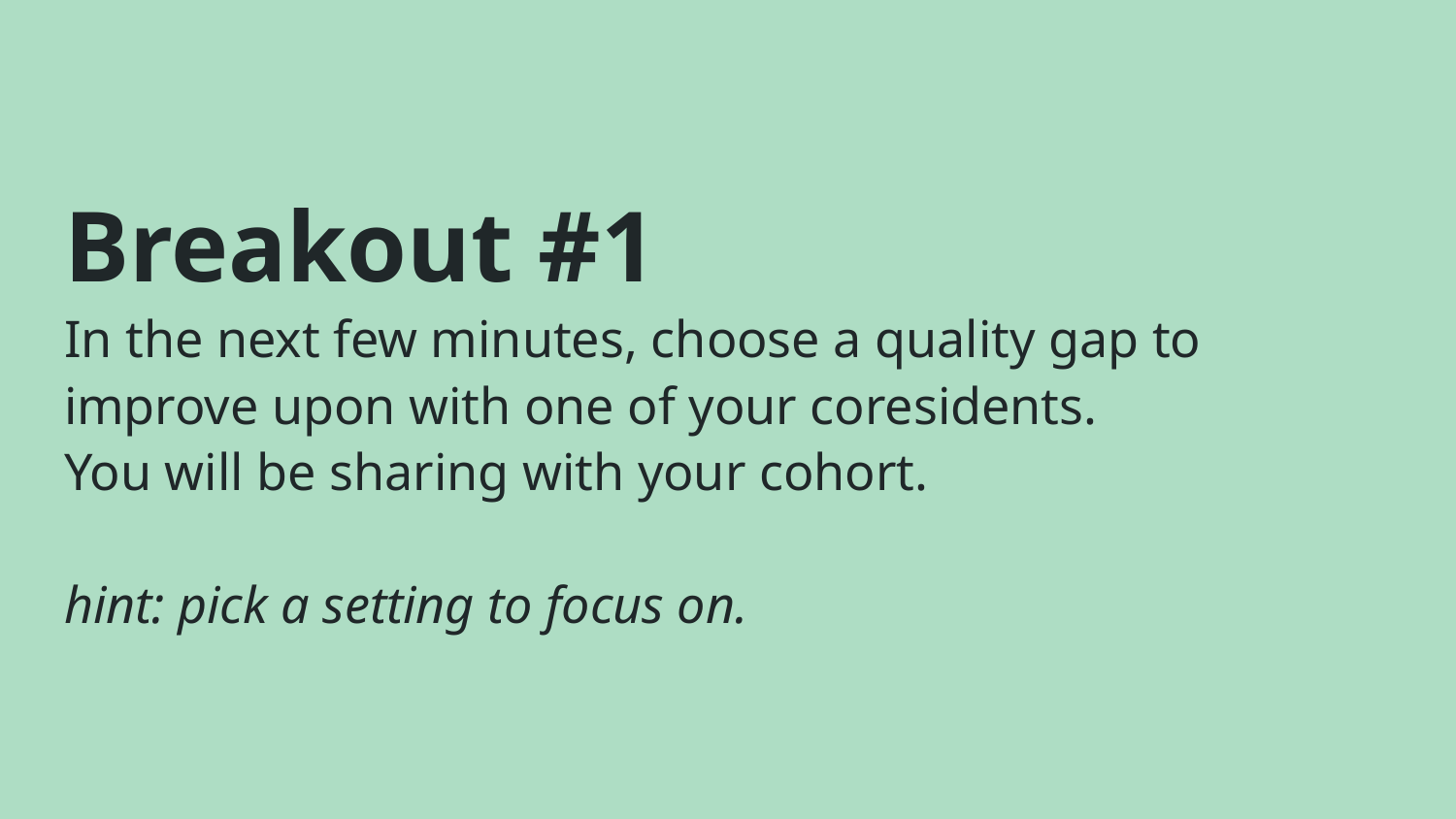

# Breakout #1
In the next few minutes, choose a quality gap to improve upon with one of your coresidents.
You will be sharing with your cohort.
hint: pick a setting to focus on.

## Slide 17
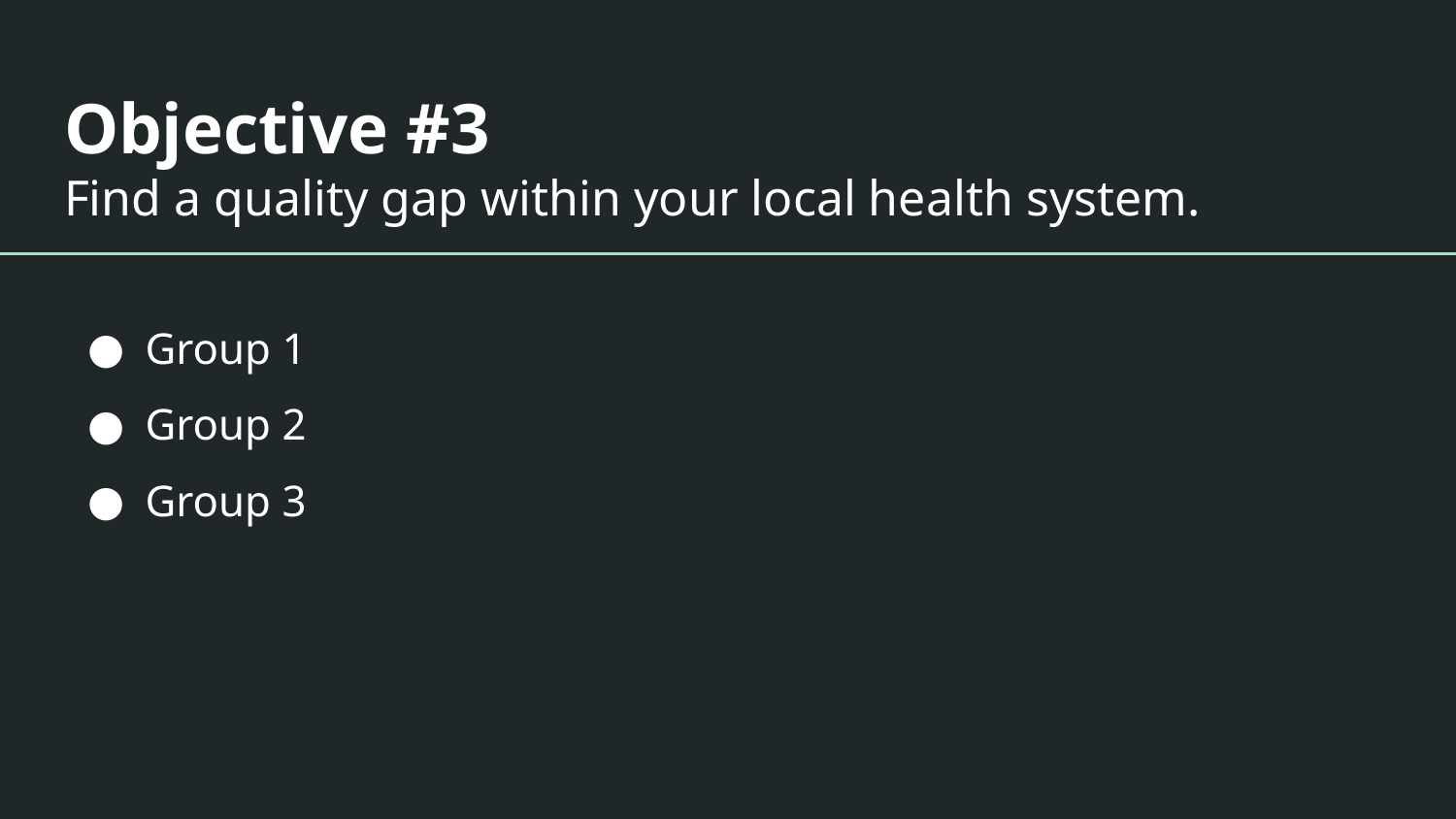

Objective #3
Find a quality gap within your local health system.
Group 1
Group 2
Group 3

## Slide 18
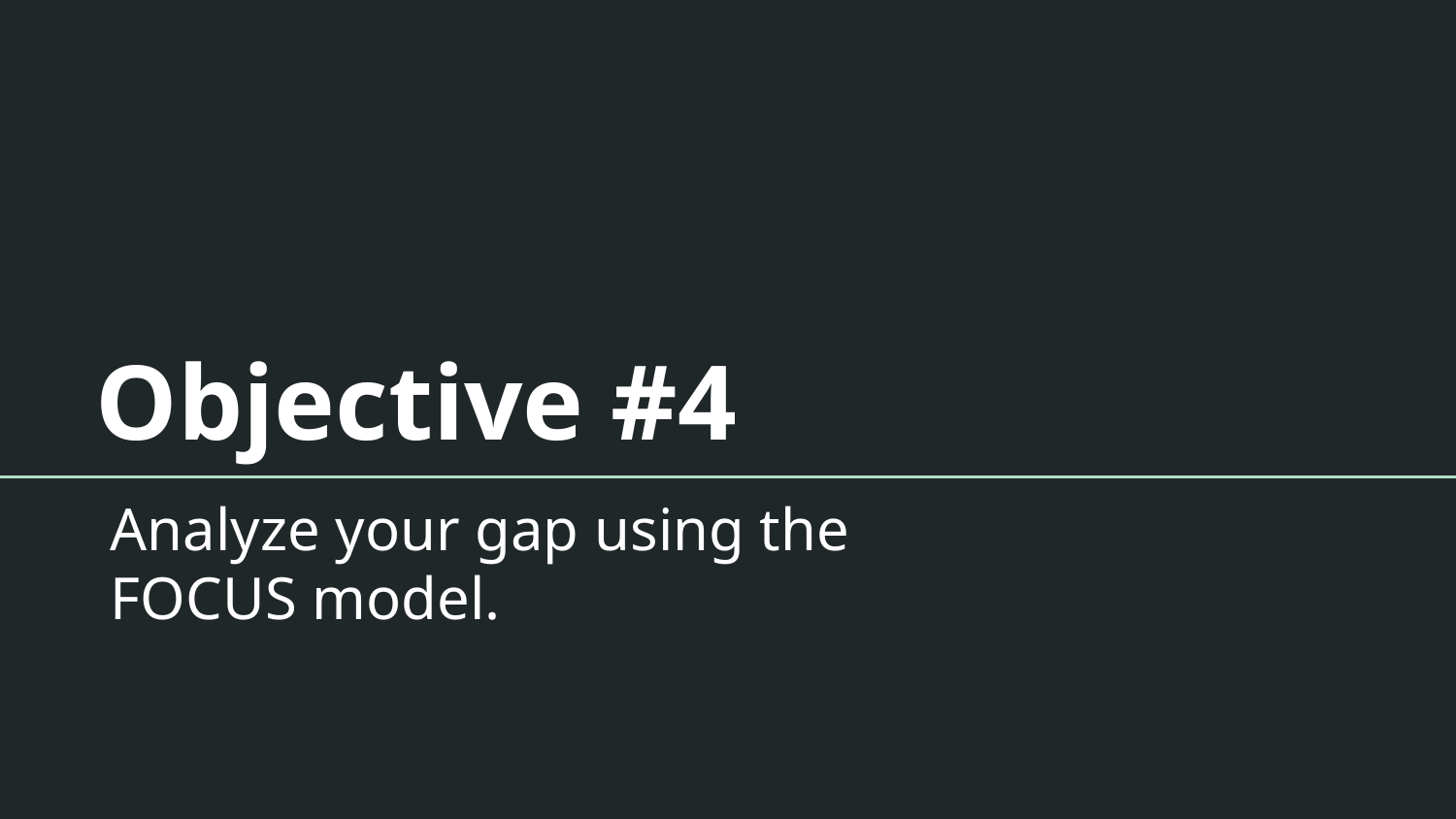

# Objective #4
Analyze your gap using the
FOCUS model.

## Slide 19
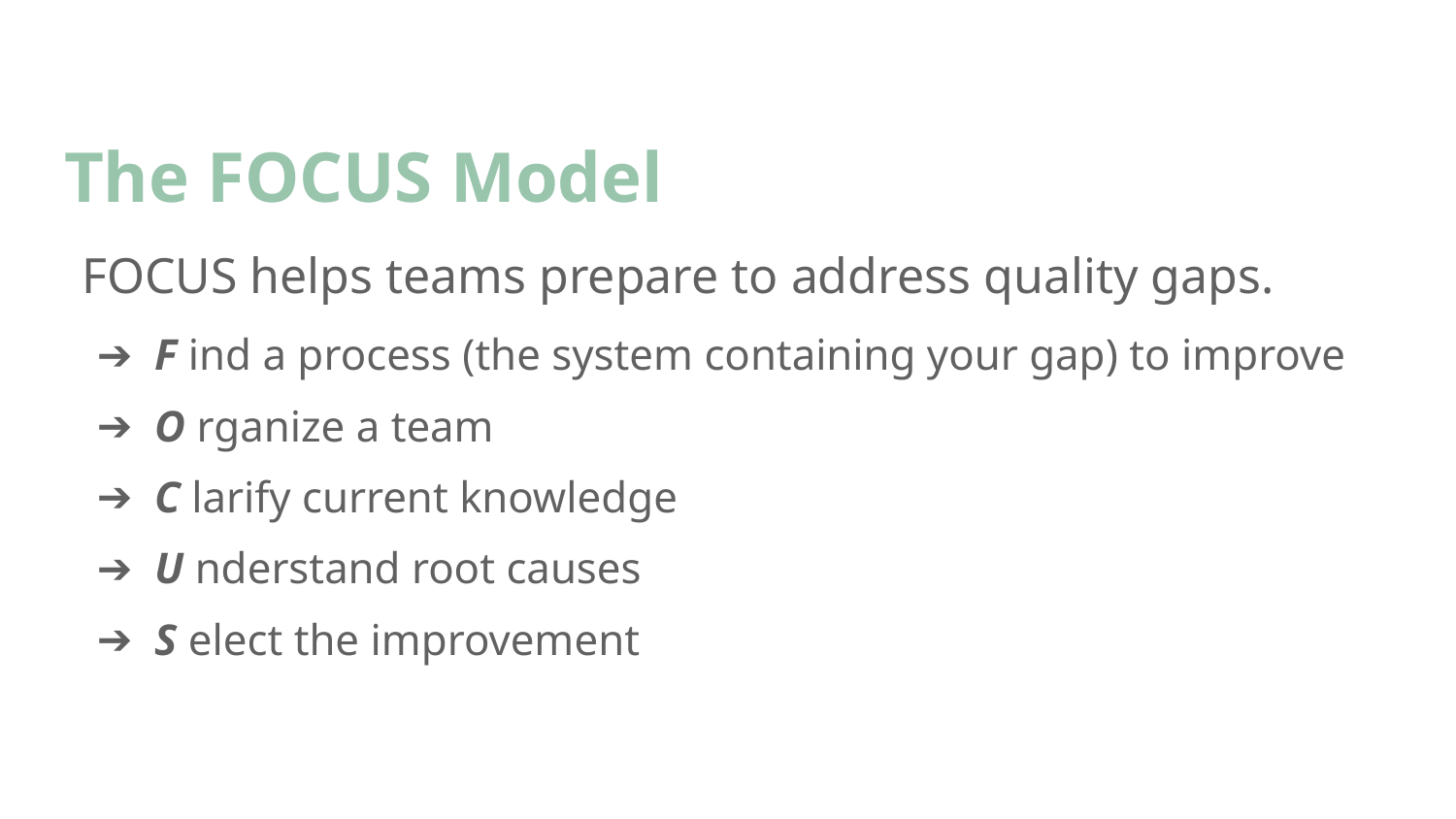

# The FOCUS Model
FOCUS helps teams prepare to address quality gaps.
F ind a process (the system containing your gap) to improve
O rganize a team
C larify current knowledge
U nderstand root causes
S elect the improvement

## Slide 20
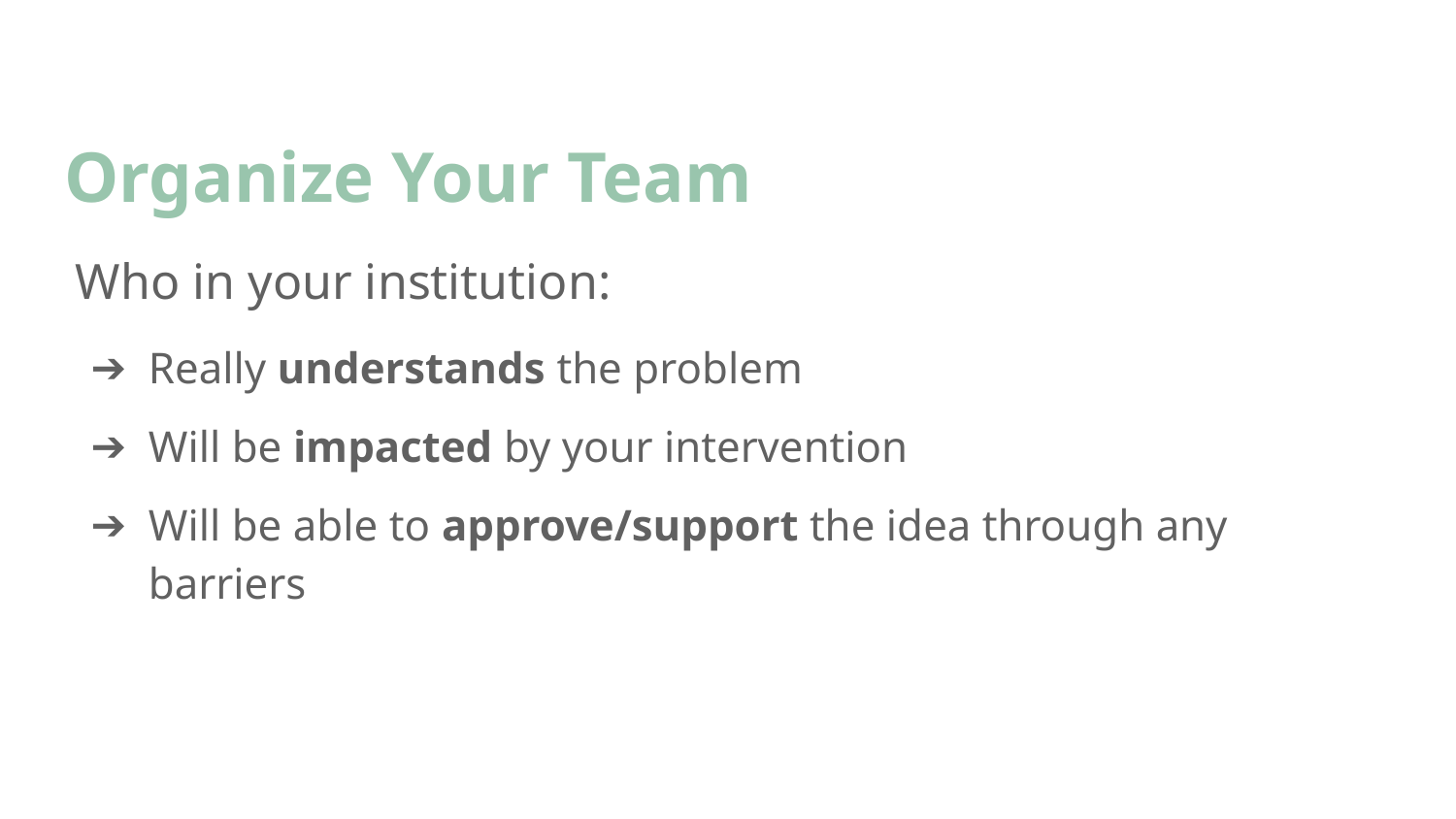

# Organize Your Team
Who in your institution:
Really understands the problem
Will be impacted by your intervention
Will be able to approve/support the idea through any barriers

## Slide 21
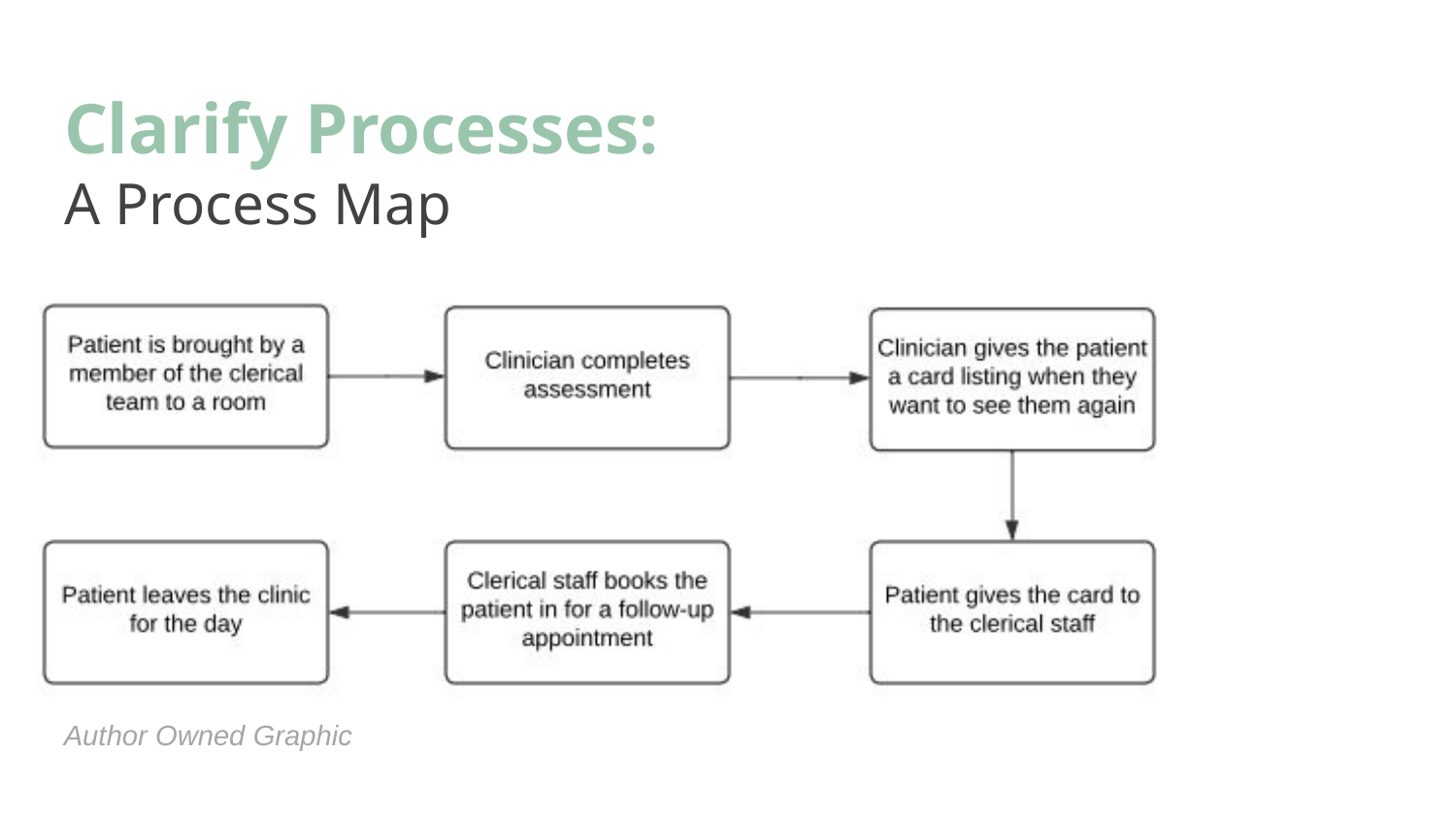

# Clarify Processes: A Process Map
Author Owned Graphic

## Slide 22
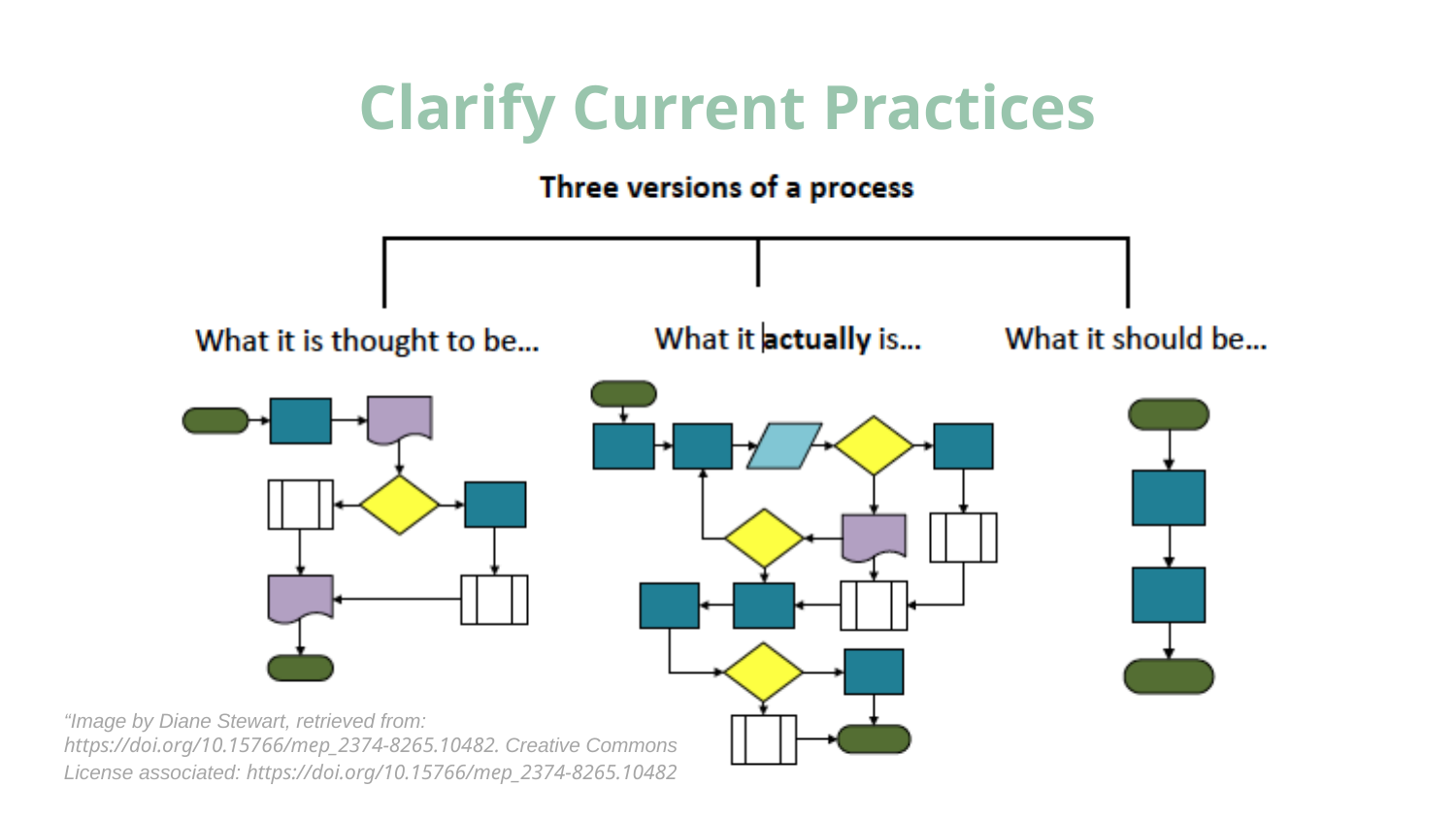

# Clarify Current Practices
“Image by Diane Stewart, retrieved from: https://doi.org/10.15766/mep_2374-8265.10482. Creative Commons License associated: https://doi.org/10.15766/mep_2374-8265.10482

## Slide 23
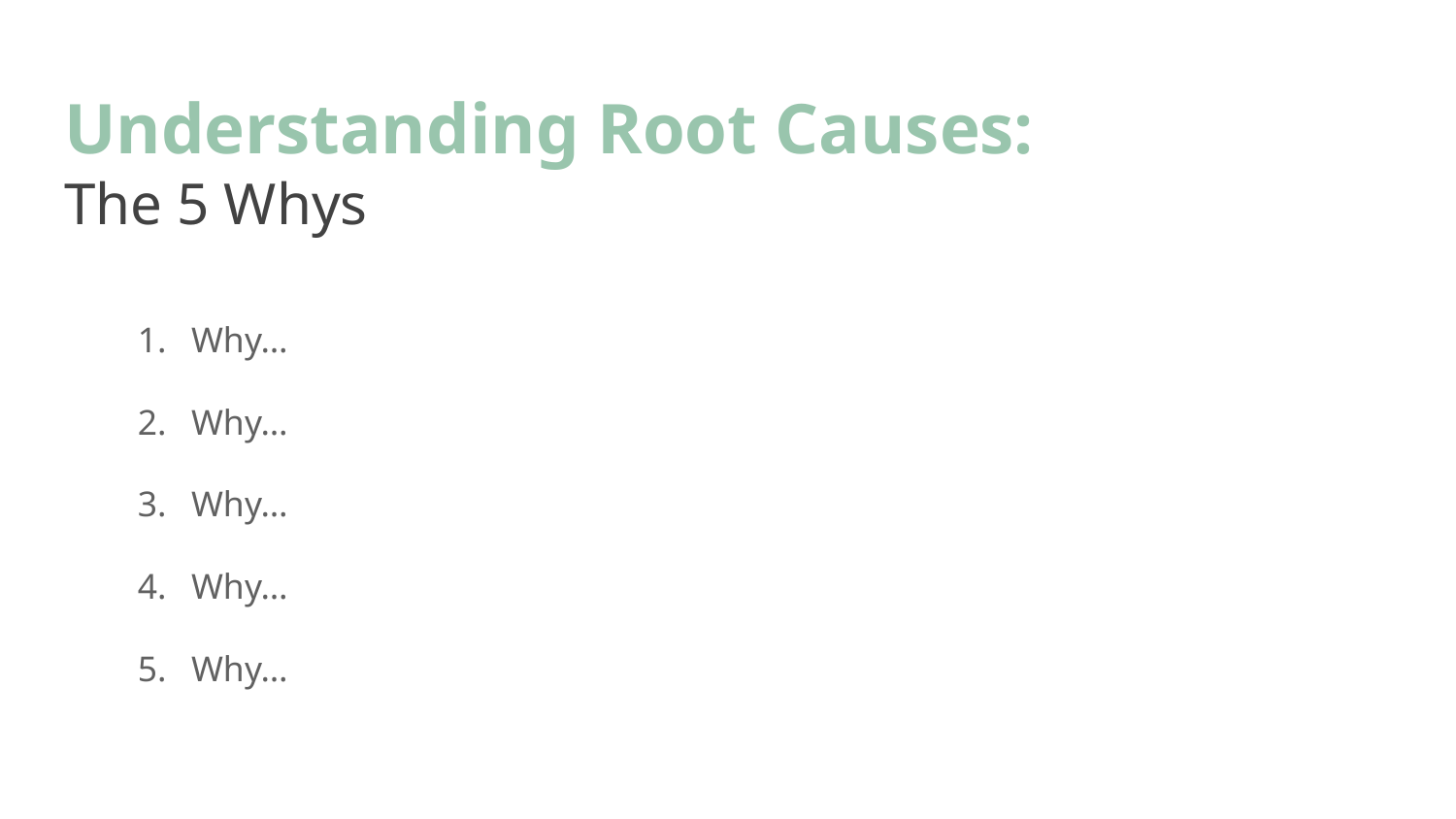

# Understanding Root Causes: The 5 Whys
Why…
Why…
Why…
Why…
Why…

## Slide 24
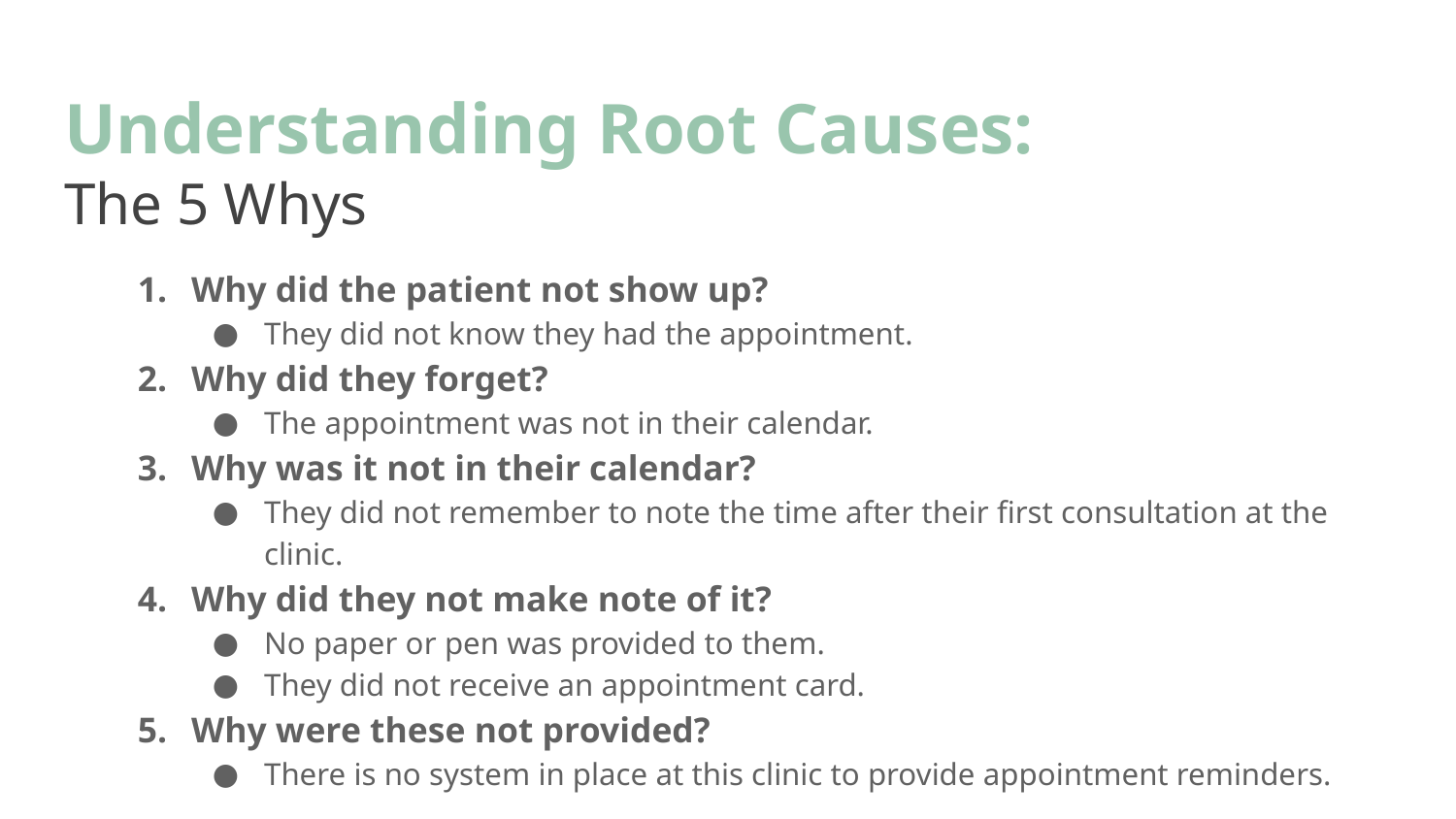

# Understanding Root Causes: The 5 Whys
Why did the patient not show up?
They did not know they had the appointment.
Why did they forget?
The appointment was not in their calendar.
Why was it not in their calendar?
They did not remember to note the time after their first consultation at the clinic.
Why did they not make note of it?
No paper or pen was provided to them.
They did not receive an appointment card.
Why were these not provided?
There is no system in place at this clinic to provide appointment reminders.

## Slide 25
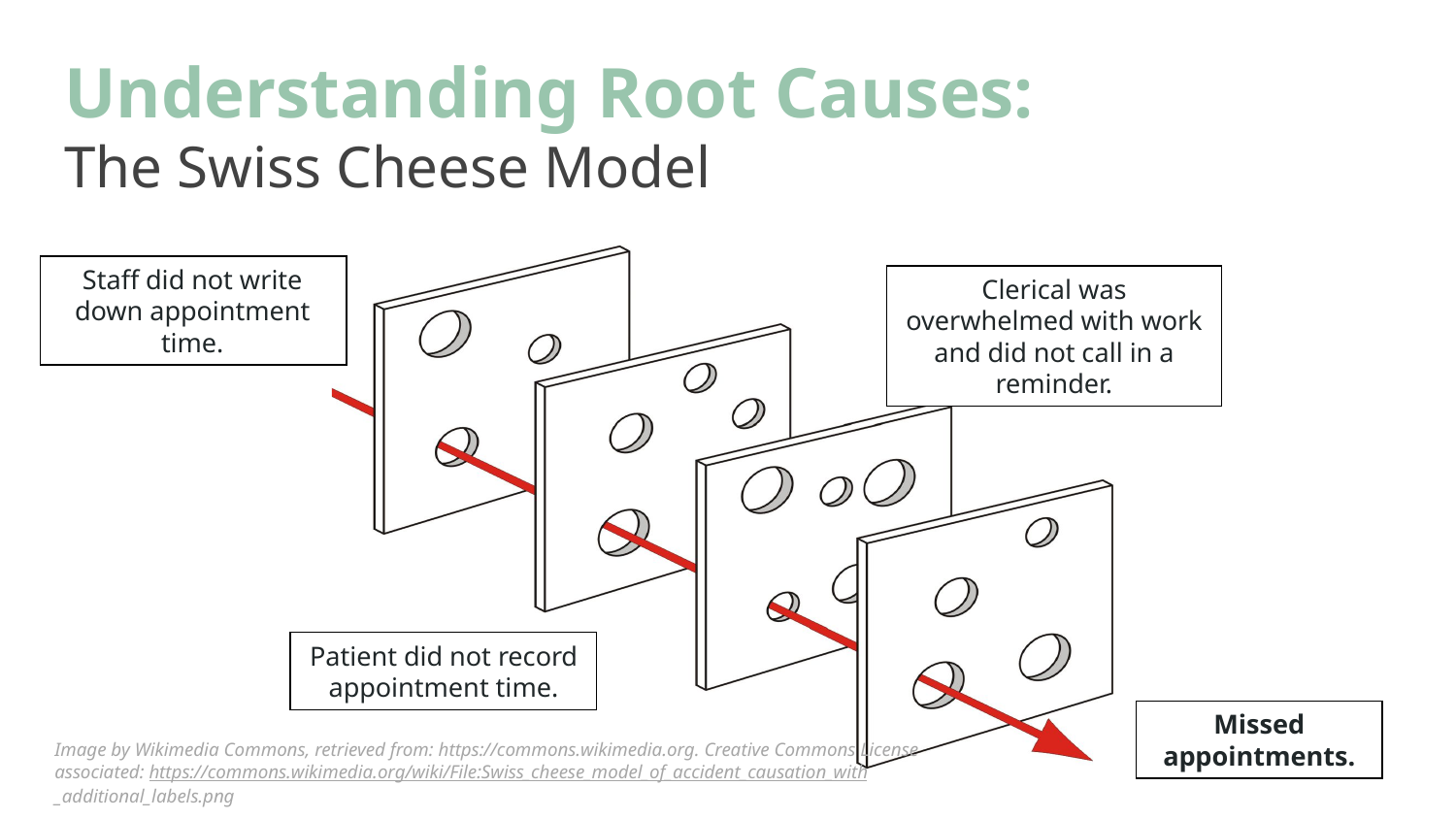

# Understanding Root Causes: The Swiss Cheese Model
Staff did not write down appointment time.
Clerical was overwhelmed with work and did not call in a reminder.
Patient did not record appointment time.
Missed appointments.
Image by Wikimedia Commons, retrieved from: https://commons.wikimedia.org. Creative Commons License associated: https://commons.wikimedia.org/wiki/File:Swiss_cheese_model_of_accident_causation_with _additional_labels.png

## Slide 26
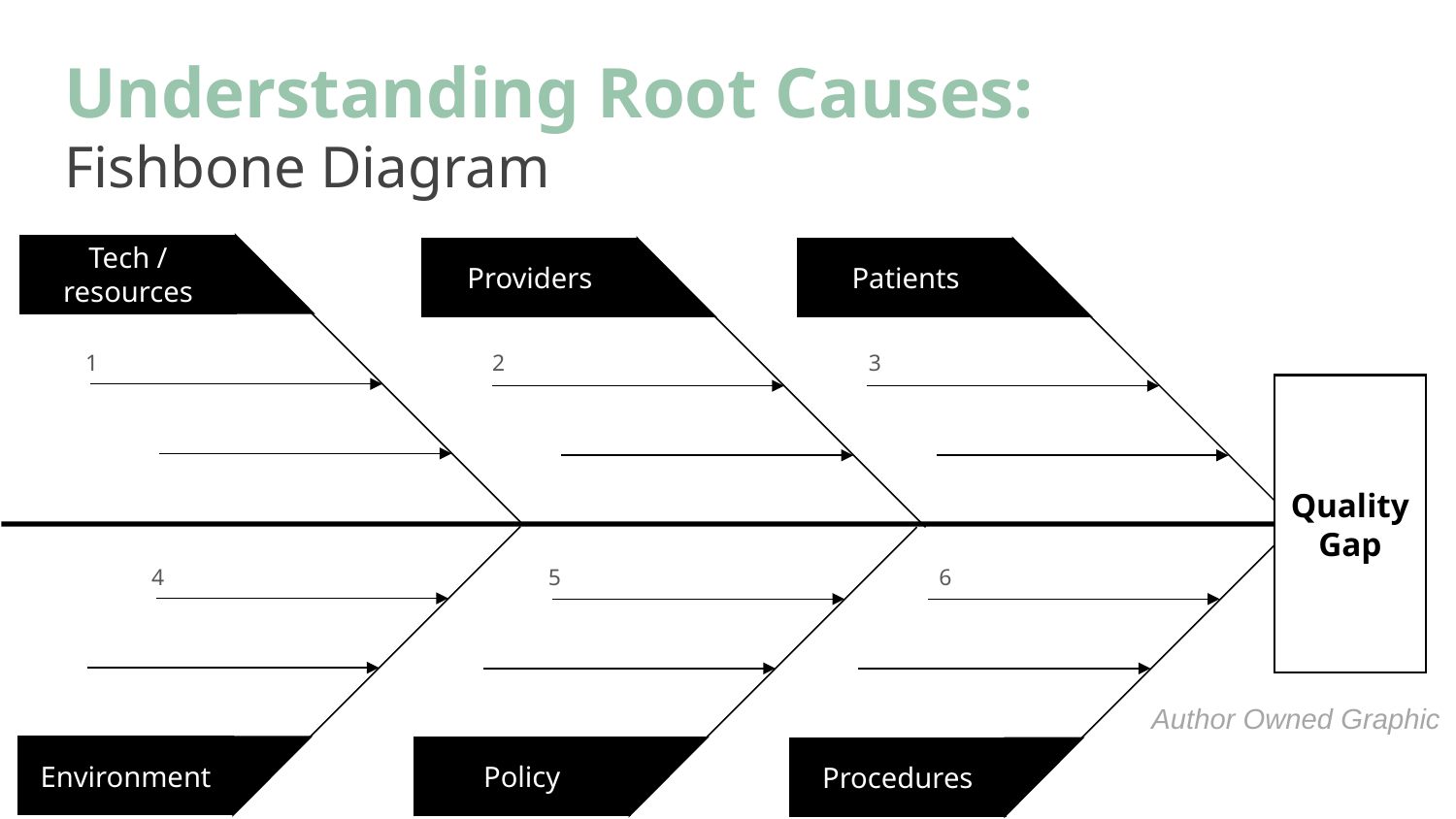

# Understanding Root Causes: Fishbone Diagram
Tech / resources
Patients
Providers
2
3
1
Quality Gap
4
5
6
Author Owned Graphic
Environment
Policy
Procedures

## Slide 27
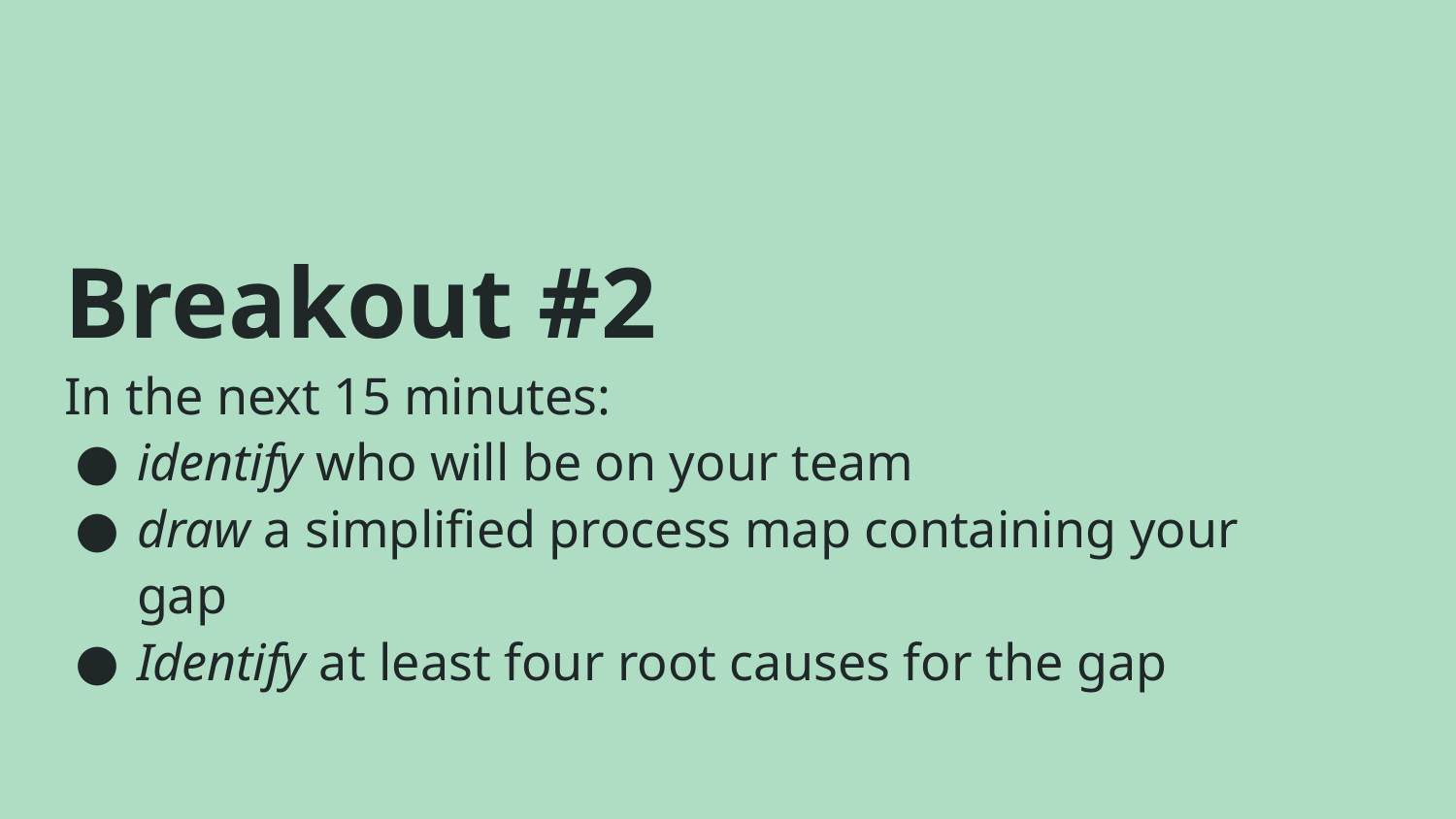

# Breakout #2
In the next 15 minutes:
identify who will be on your team
draw a simplified process map containing your gap
Identify at least four root causes for the gap

## Slide 28
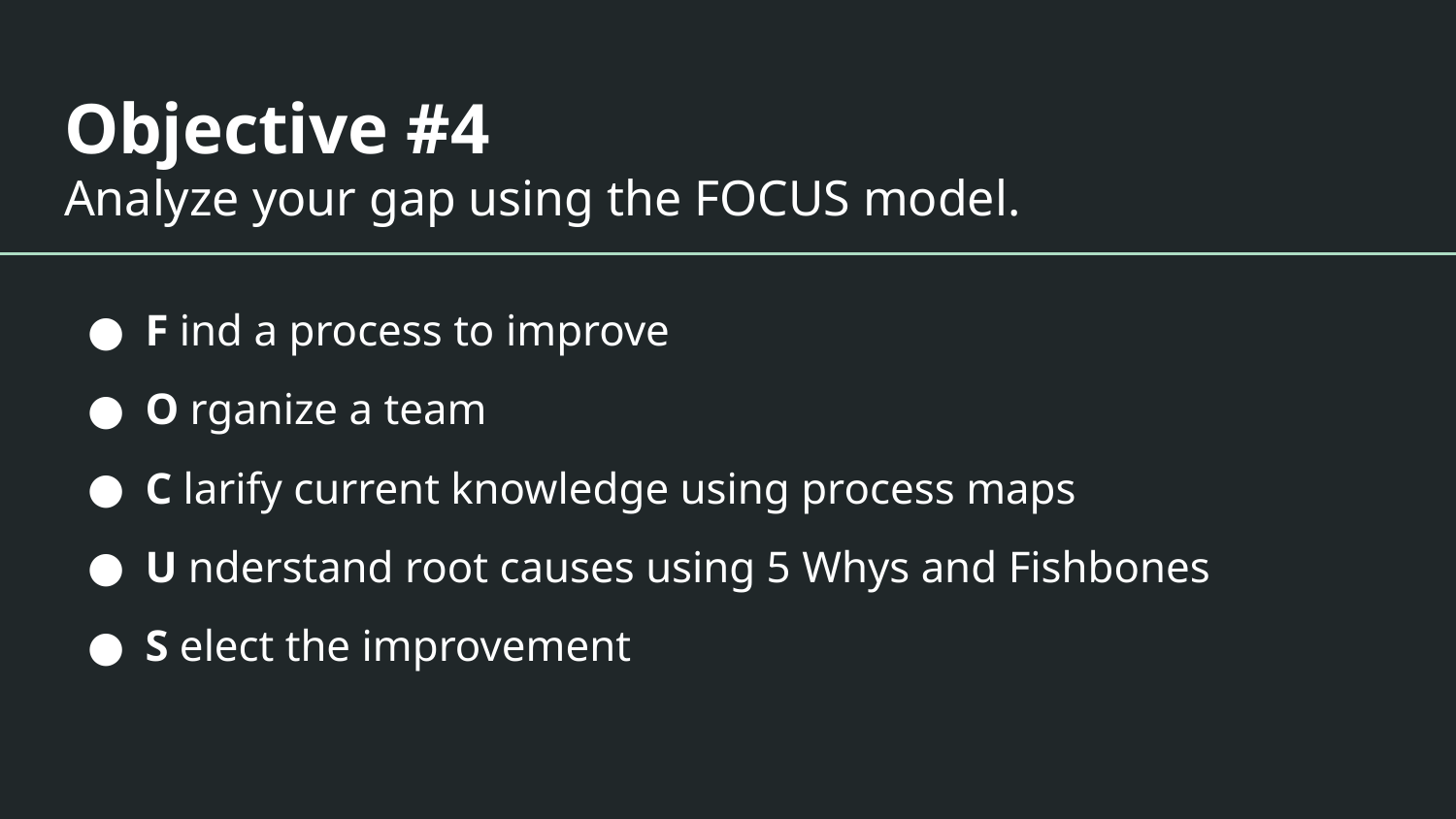

Objective #4
Analyze your gap using the FOCUS model.
F ind a process to improve
O rganize a team
C larify current knowledge using process maps
U nderstand root causes using 5 Whys and Fishbones
S elect the improvement

## Slide 29
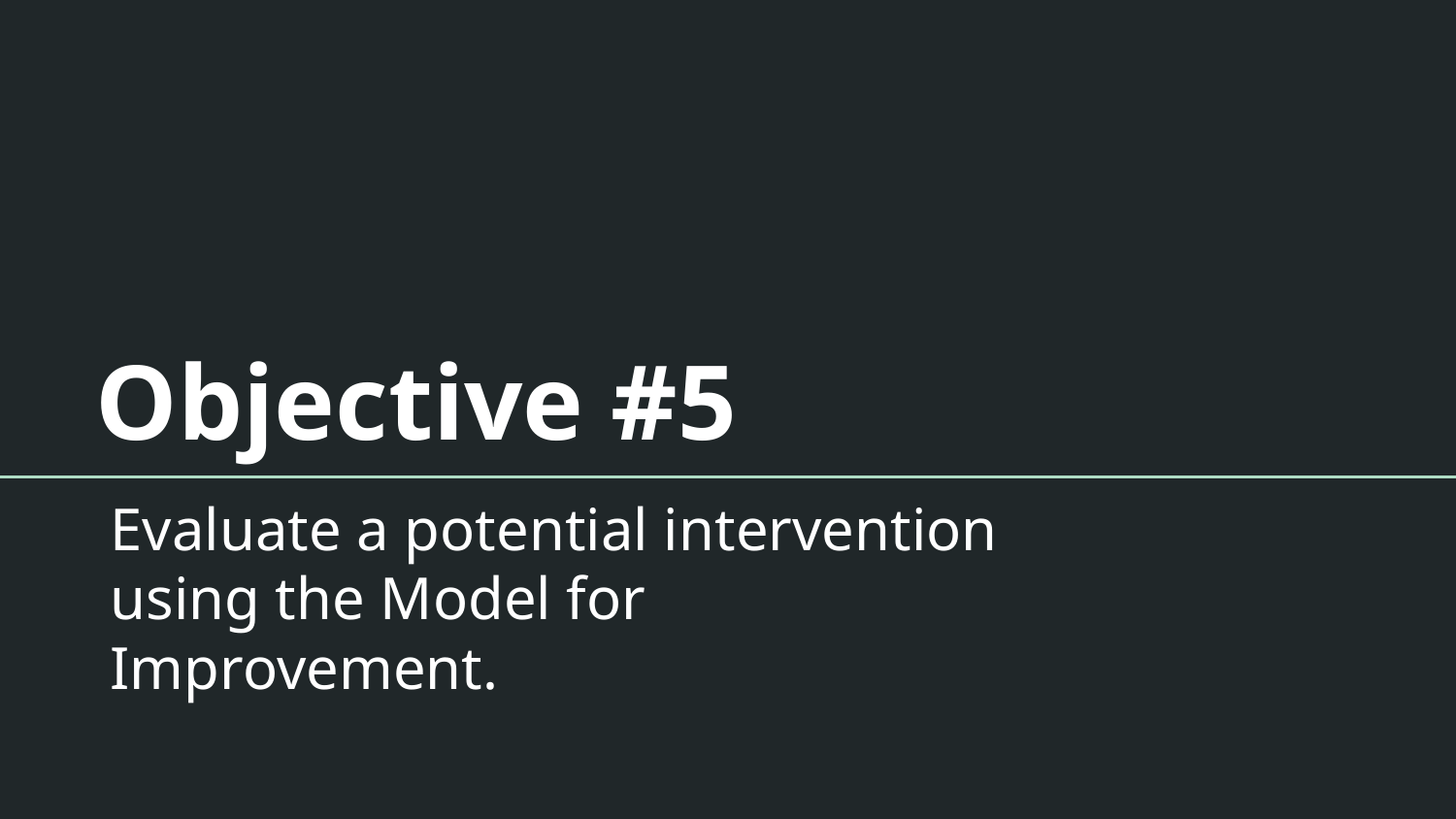

# Objective #5
Evaluate a potential intervention
using the Model for Improvement.

## Slide 30
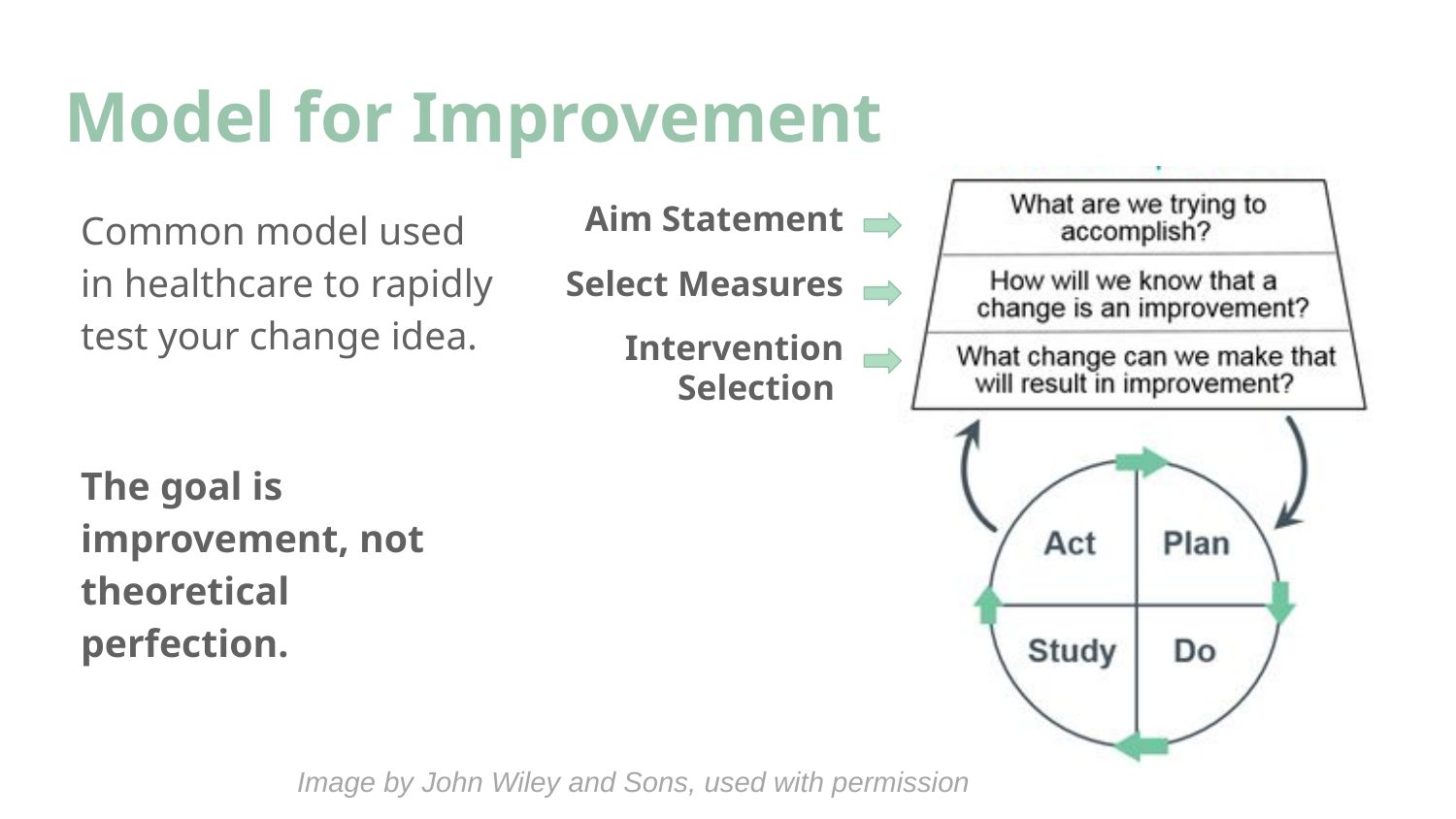

# Model for Improvement
Common model used in healthcare to rapidly test your change idea.
The goal is improvement, not theoretical perfection.
Aim Statement
Select Measures
Intervention Selection
Image by John Wiley and Sons, used with permission

## Slide 31
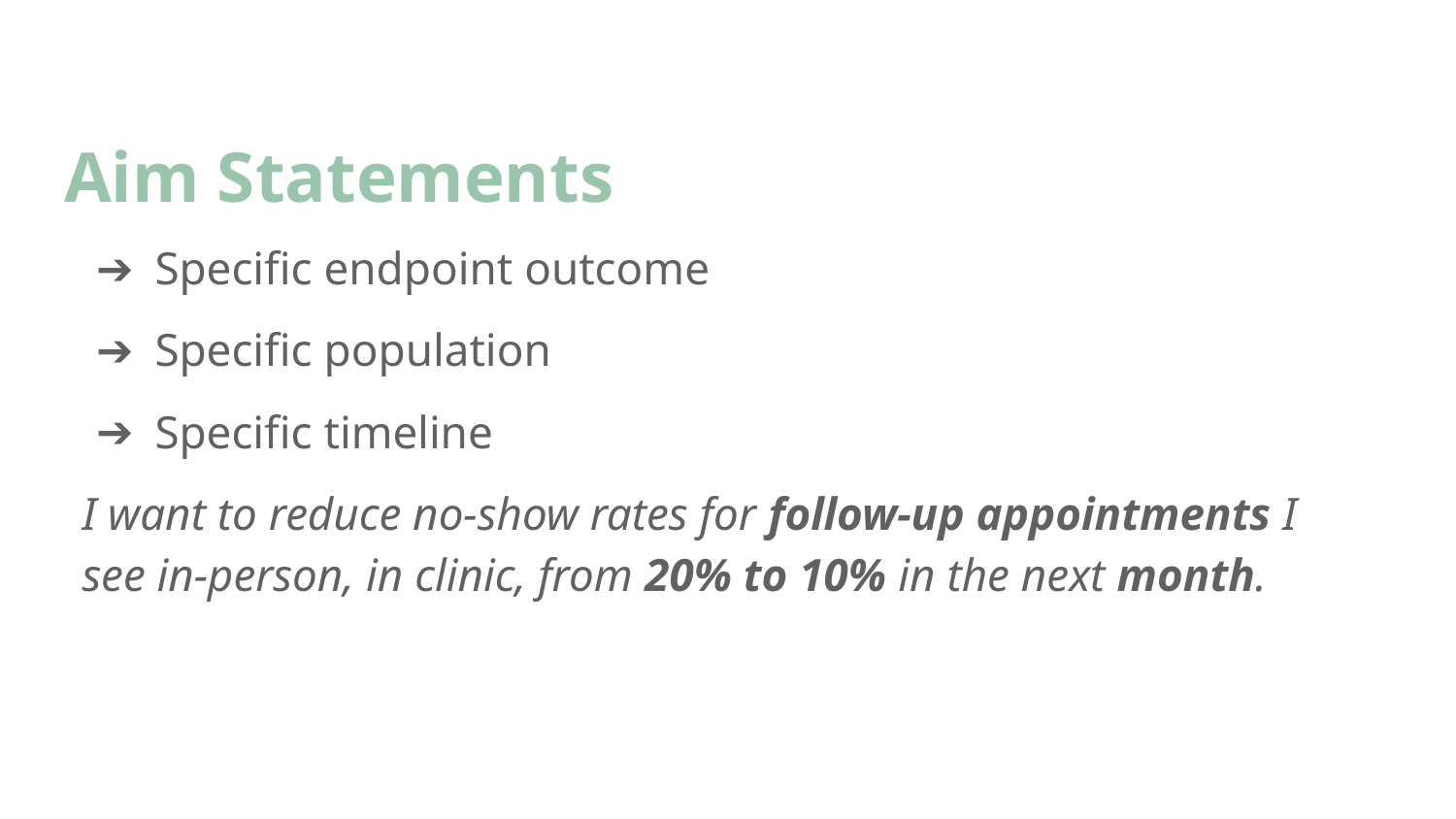

# Aim Statements
Specific endpoint outcome
Specific population
Specific timeline
I want to reduce no-show rates for follow-up appointments I see in-person, in clinic, from 20% to 10% in the next month.

## Slide 32
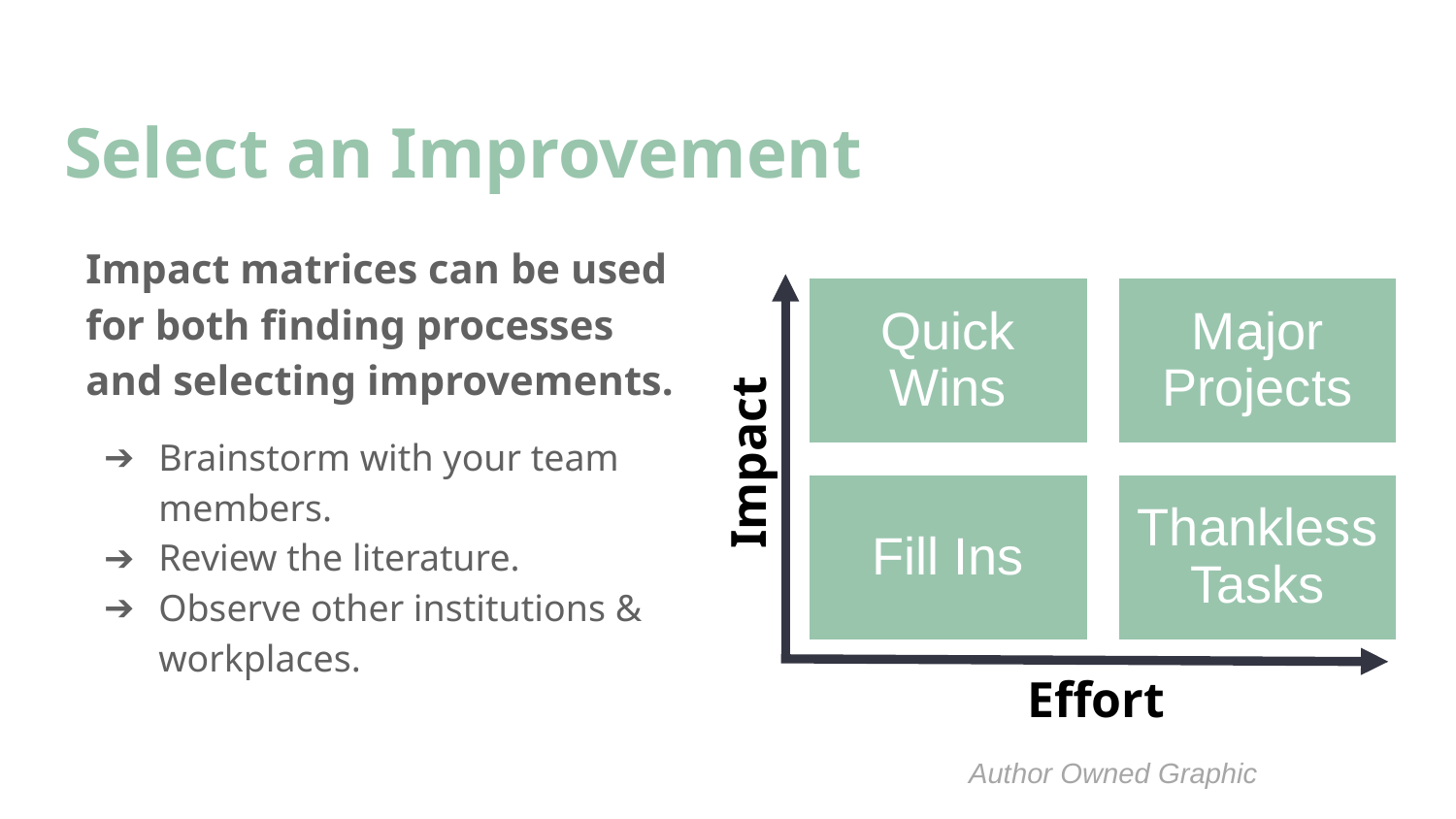

# Select an Improvement
Impact matrices can be used for both finding processes and selecting improvements.
Brainstorm with your team members.
Review the literature.
Observe other institutions & workplaces.
Impact
Effort
Author Owned Graphic

## Slide 33
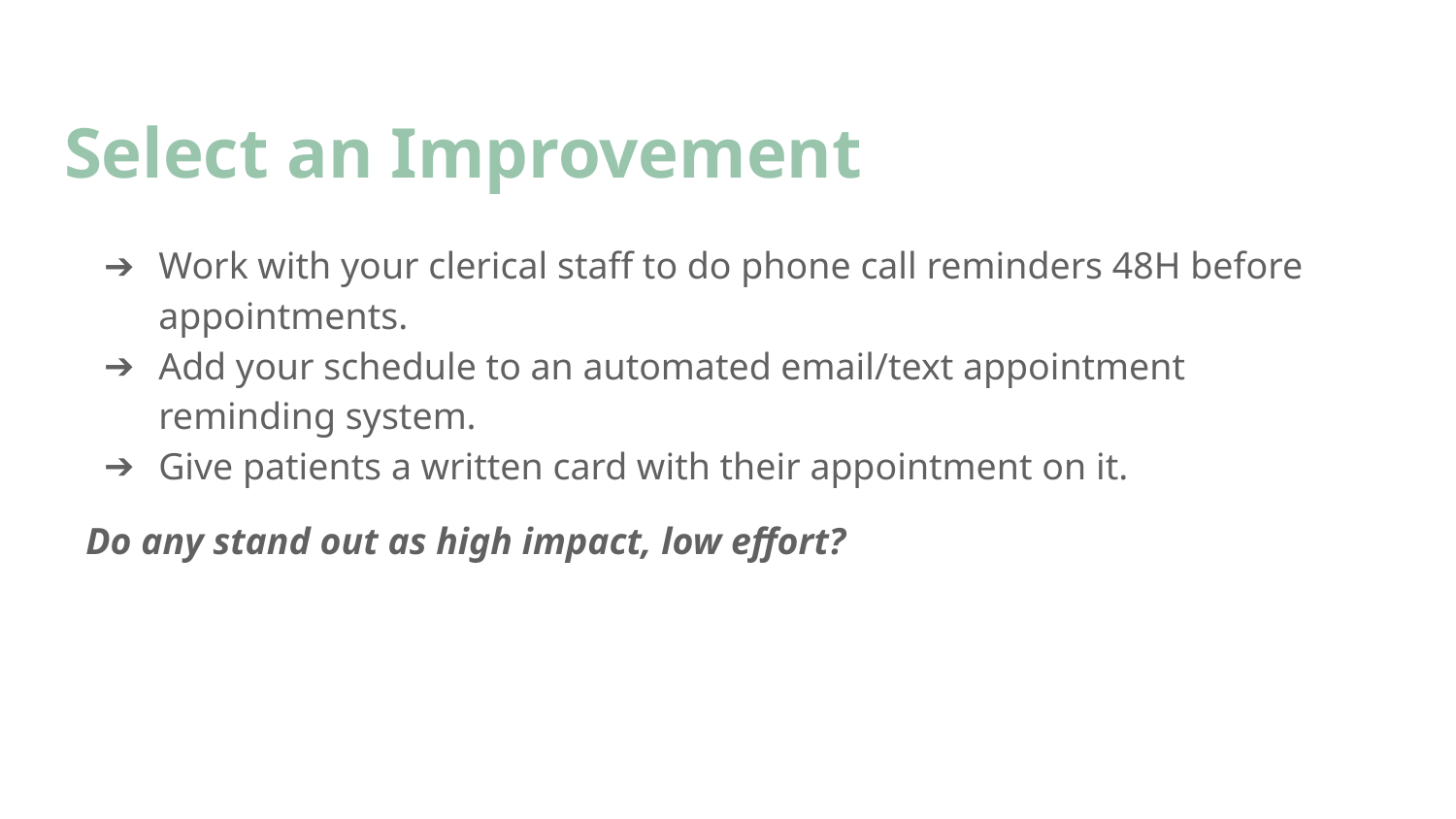

# Select an Improvement
Work with your clerical staff to do phone call reminders 48H before appointments.
Add your schedule to an automated email/text appointment reminding system.
Give patients a written card with their appointment on it.
Do any stand out as high impact, low effort?

## Slide 34
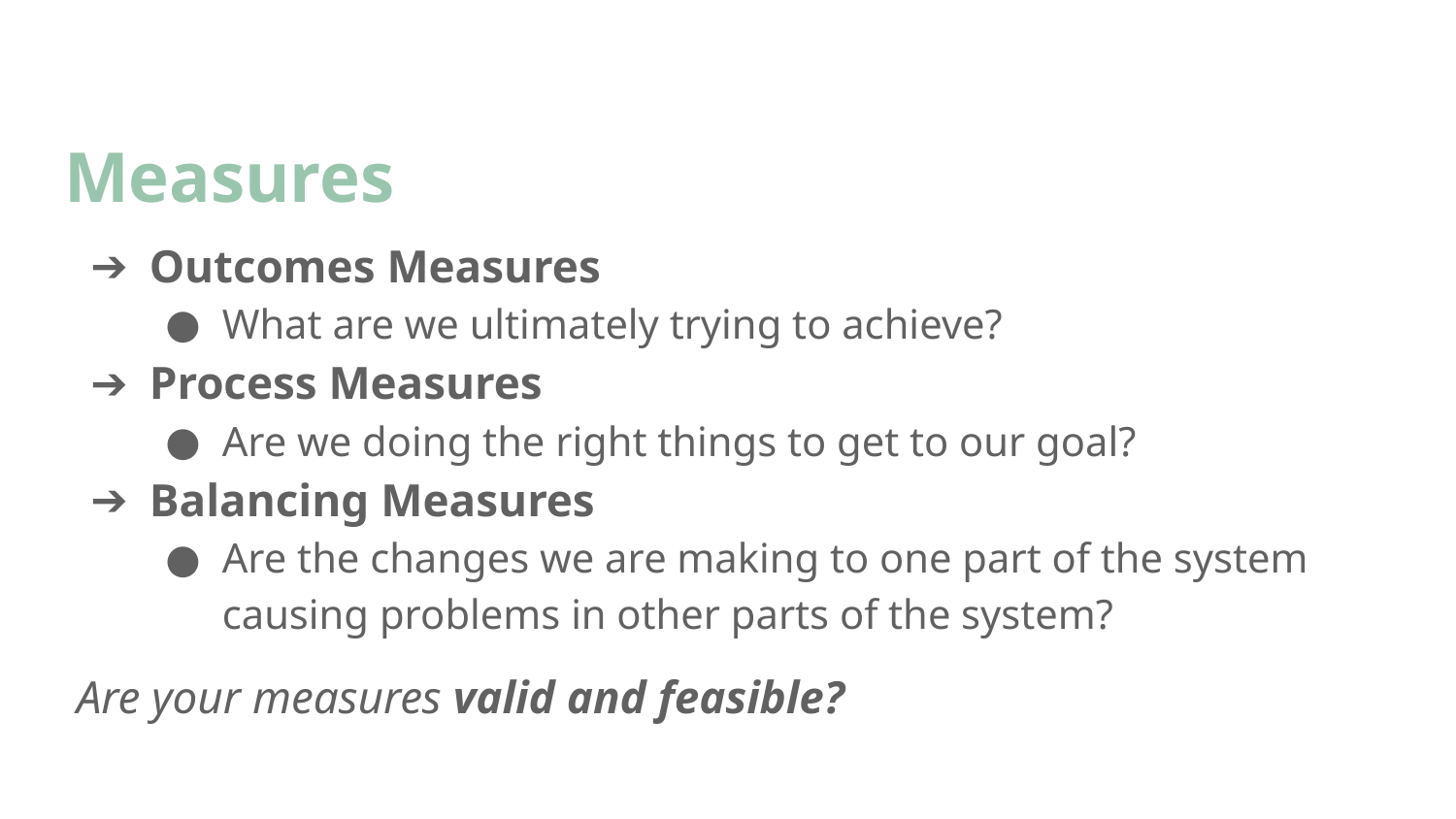

# Measures
Outcomes Measures
What are we ultimately trying to achieve?
Process Measures
Are we doing the right things to get to our goal?
Balancing Measures
Are the changes we are making to one part of the system causing problems in other parts of the system?
Are your measures valid and feasible?

## Slide 35
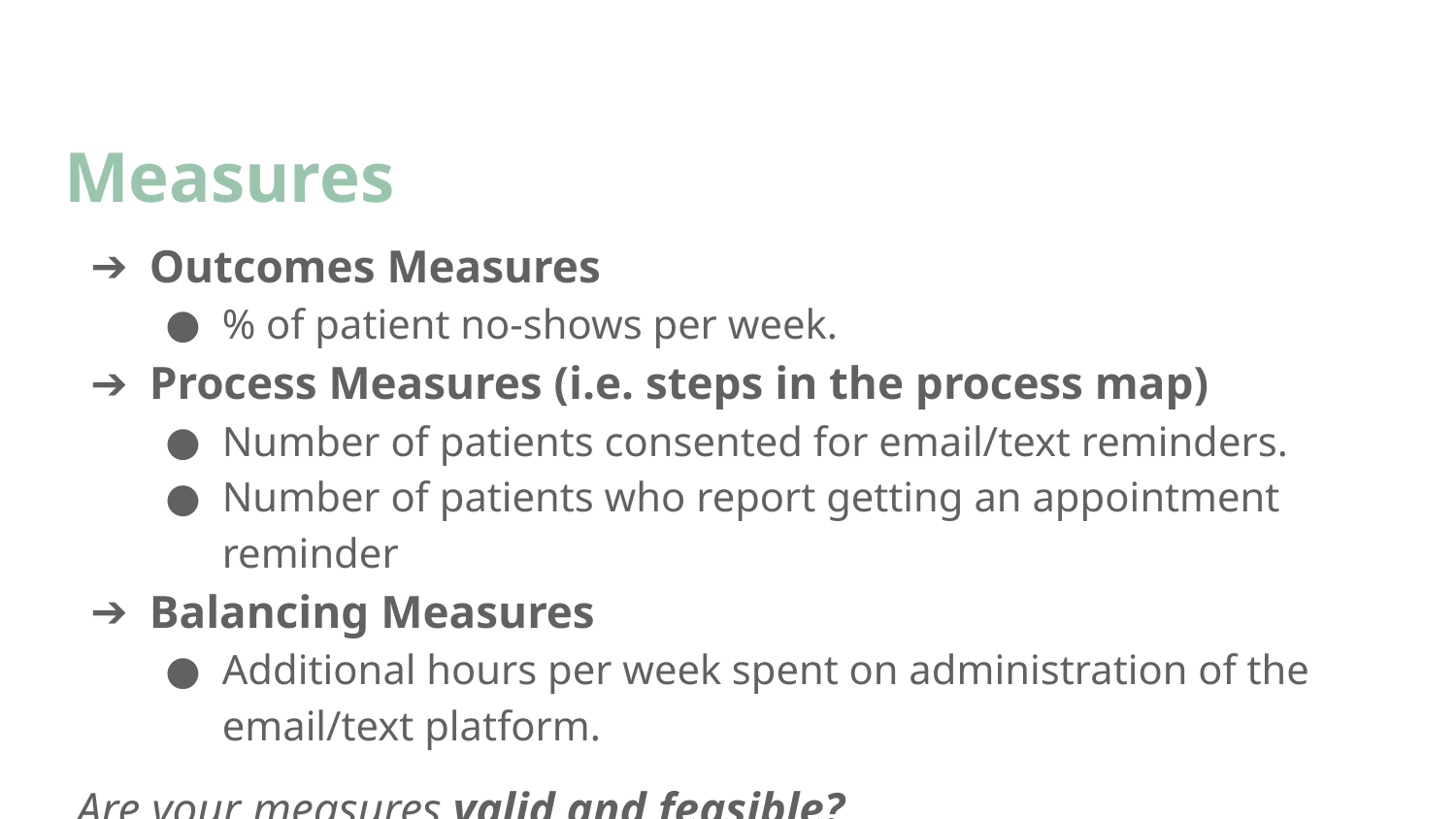

# Measures
Outcomes Measures
% of patient no-shows per week.
Process Measures (i.e. steps in the process map)
Number of patients consented for email/text reminders.
Number of patients who report getting an appointment reminder
Balancing Measures
Additional hours per week spent on administration of the email/text platform.
Are your measures valid and feasible?

## Slide 36
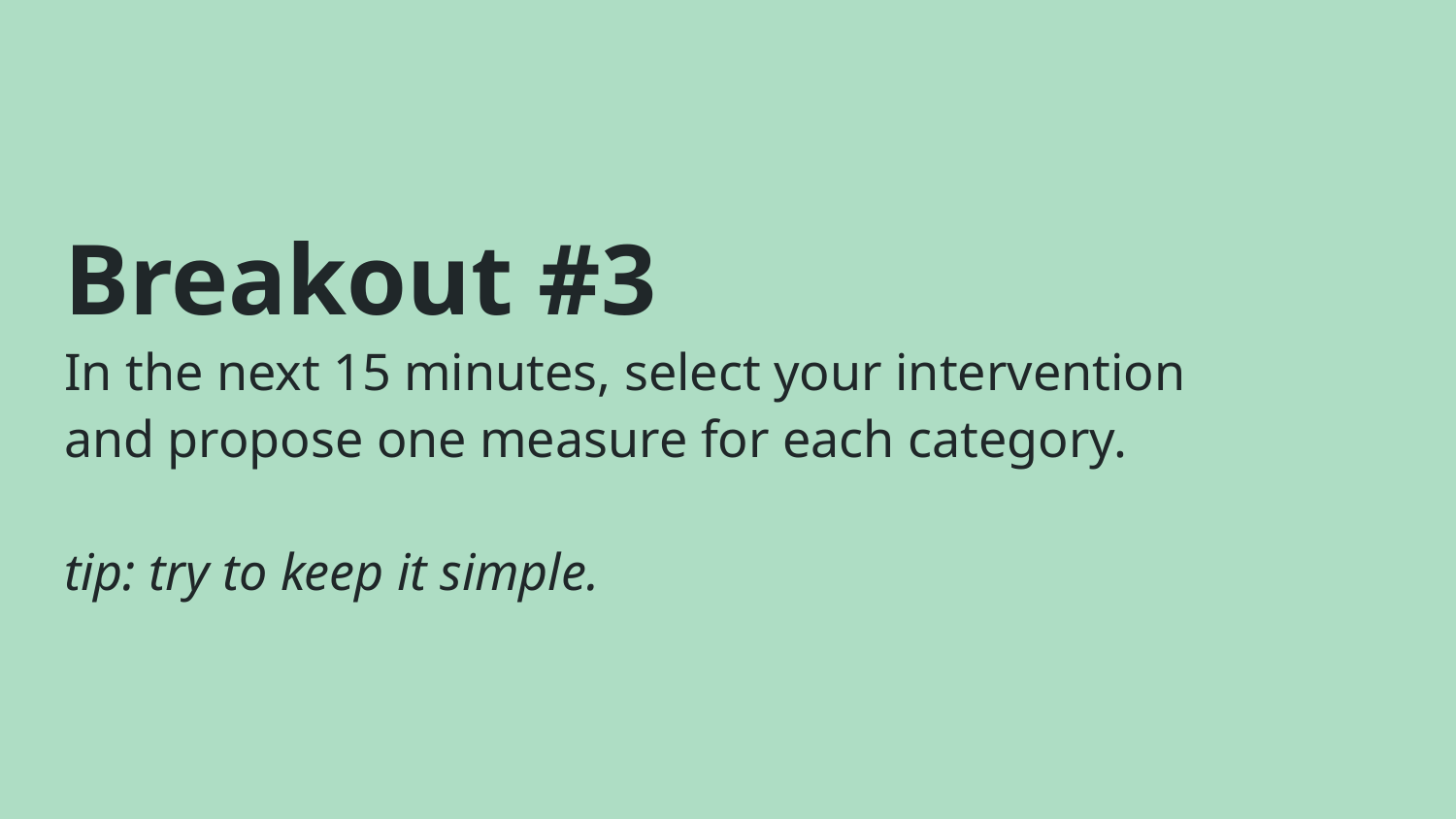

# Breakout #3
In the next 15 minutes, select your intervention and propose one measure for each category.
tip: try to keep it simple.

## Slide 37
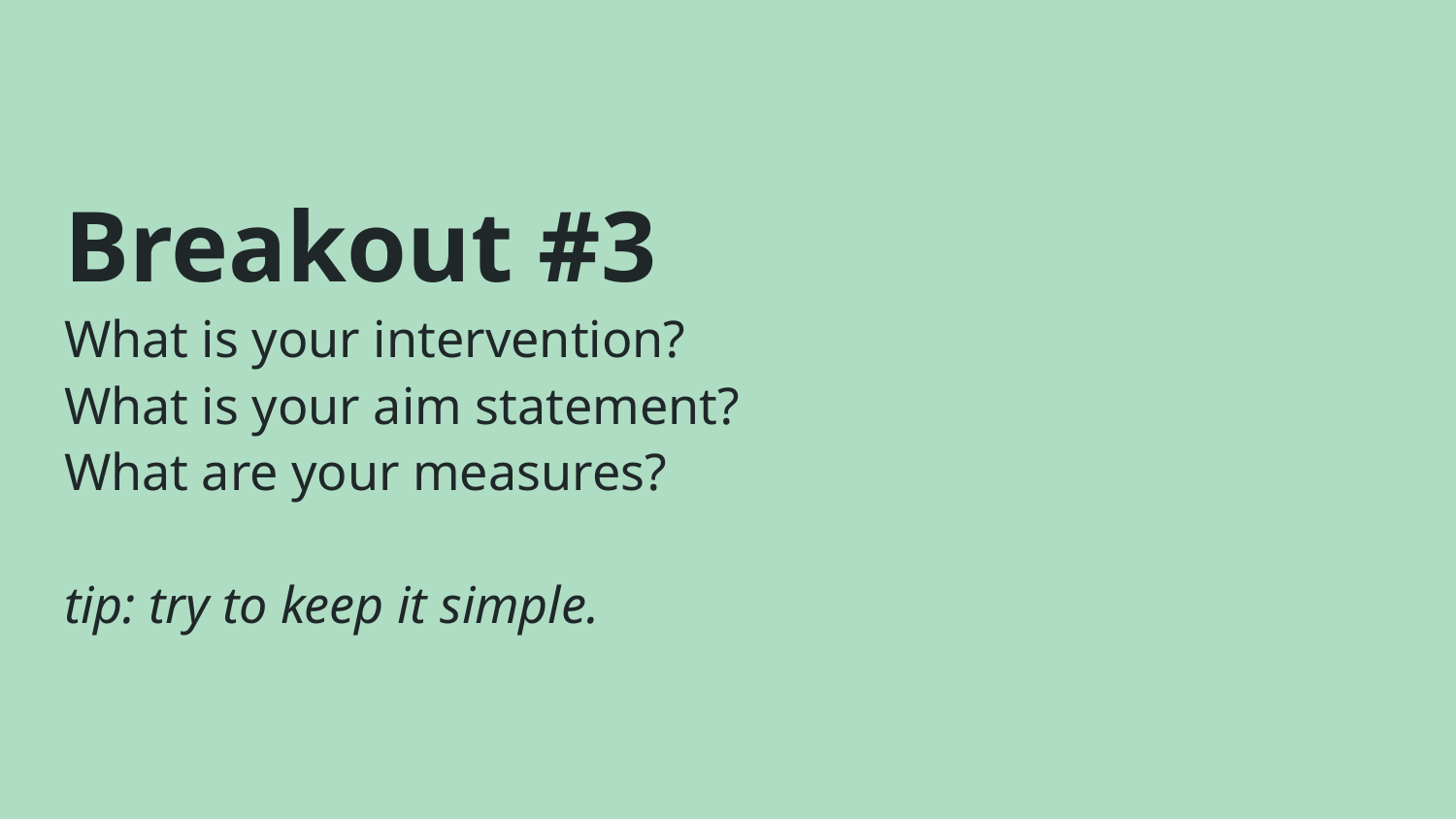

# Breakout #3
What is your intervention?
What is your aim statement?
What are your measures?
tip: try to keep it simple.

## Slide 38
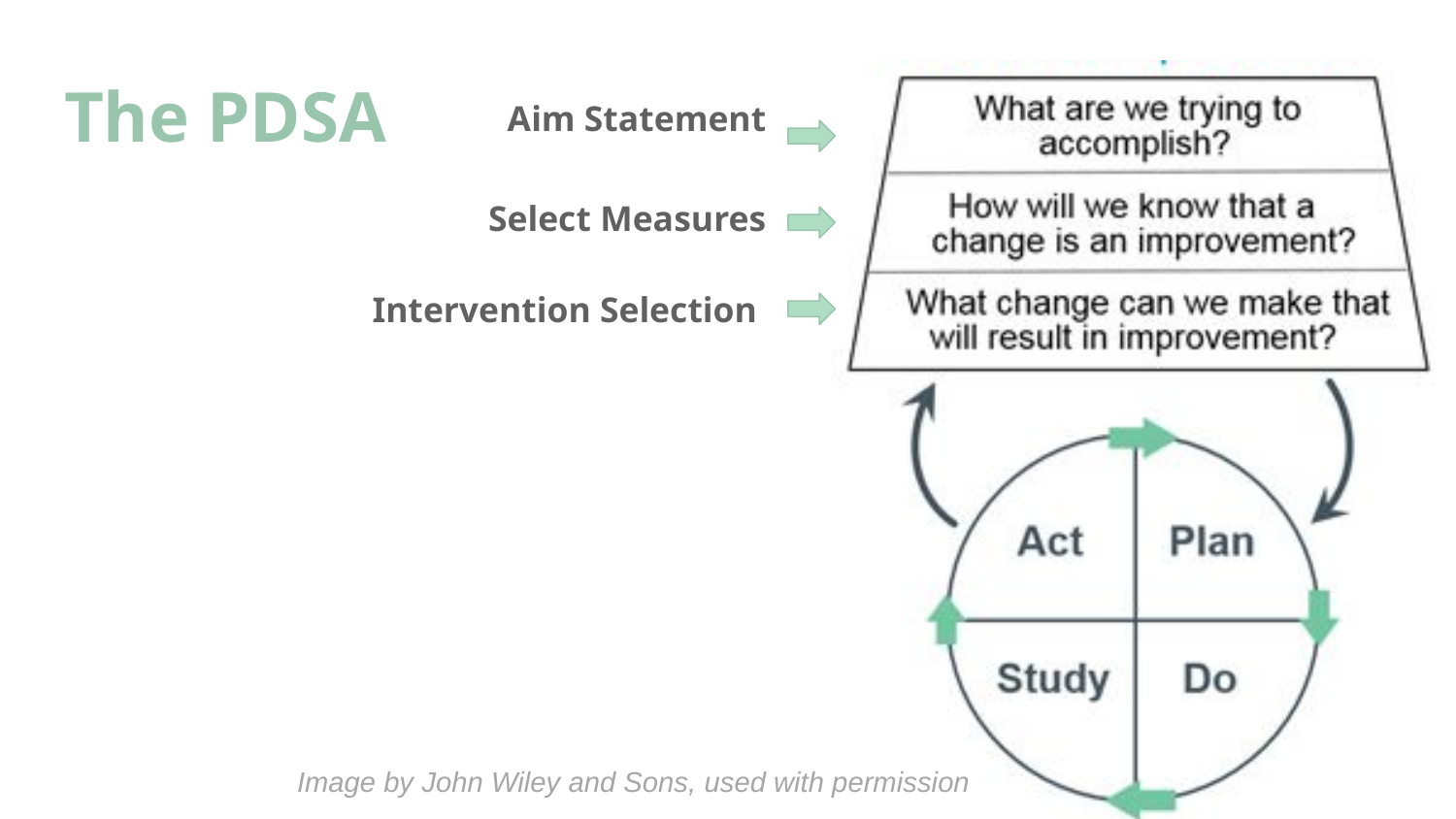

# The PDSA
Aim Statement
Select Measures
Intervention Selection
Image by John Wiley and Sons, used with permission

## Slide 39
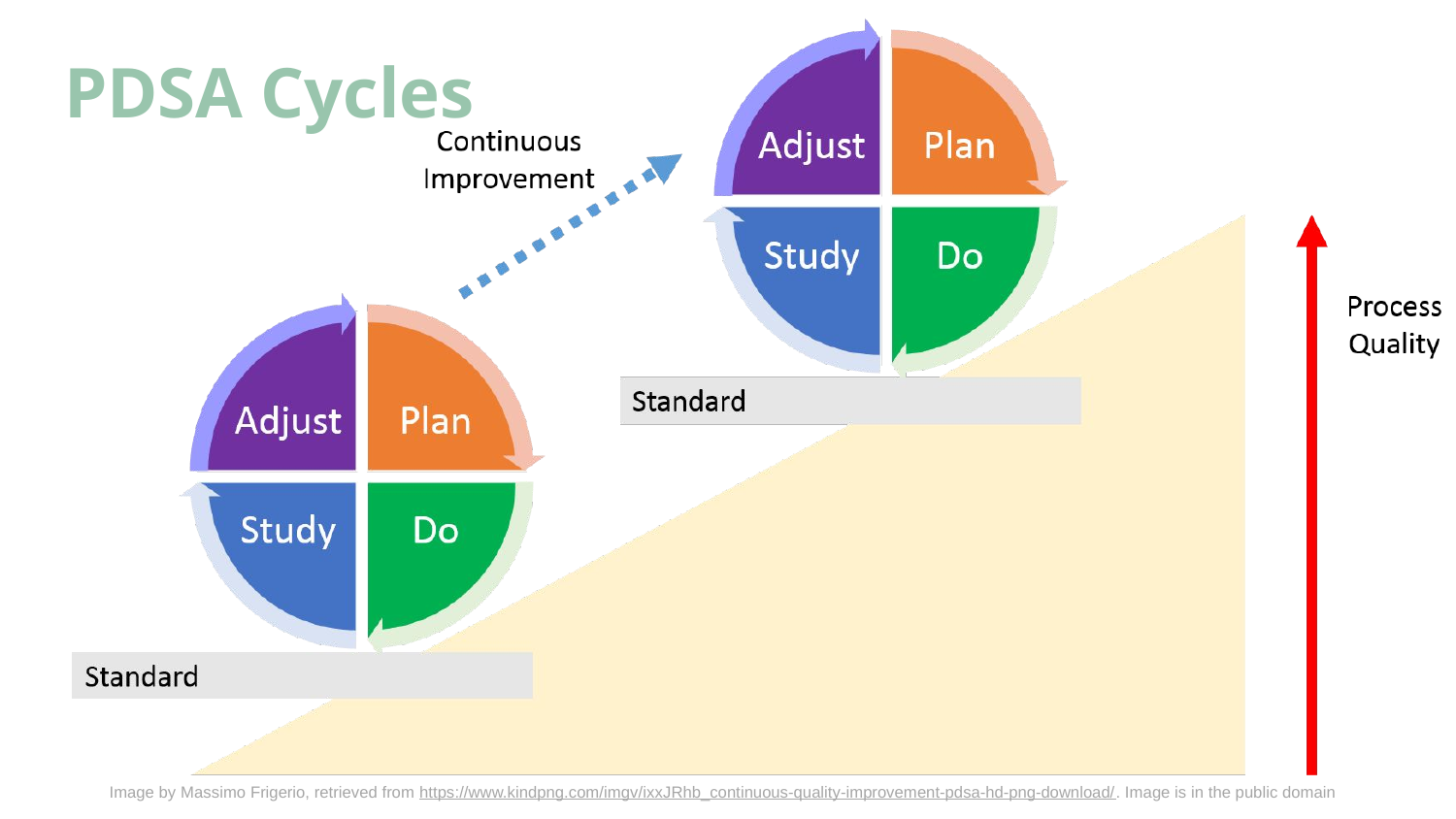

# PDSA Cycles
Image by Massimo Frigerio, retrieved from https://www.kindpng.com/imgv/ixxJRhb_continuous-quality-improvement-pdsa-hd-png-download/. Image is in the public domain

## Slide 40
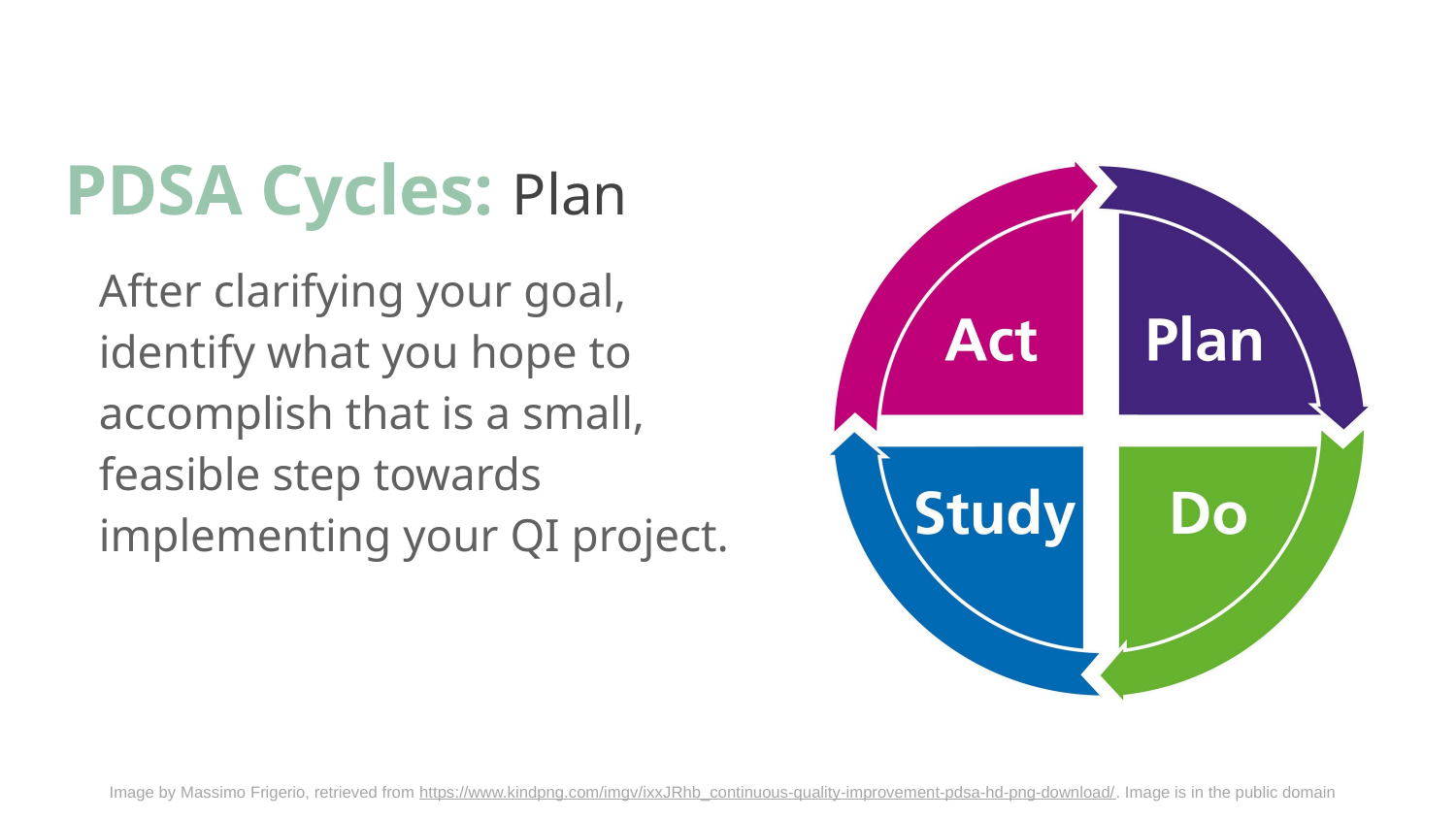

# PDSA Cycles: Plan
After clarifying your goal, identify what you hope to accomplish that is a small, feasible step towards implementing your QI project.
Image by Massimo Frigerio, retrieved from https://www.kindpng.com/imgv/ixxJRhb_continuous-quality-improvement-pdsa-hd-png-download/. Image is in the public domain

## Slide 41
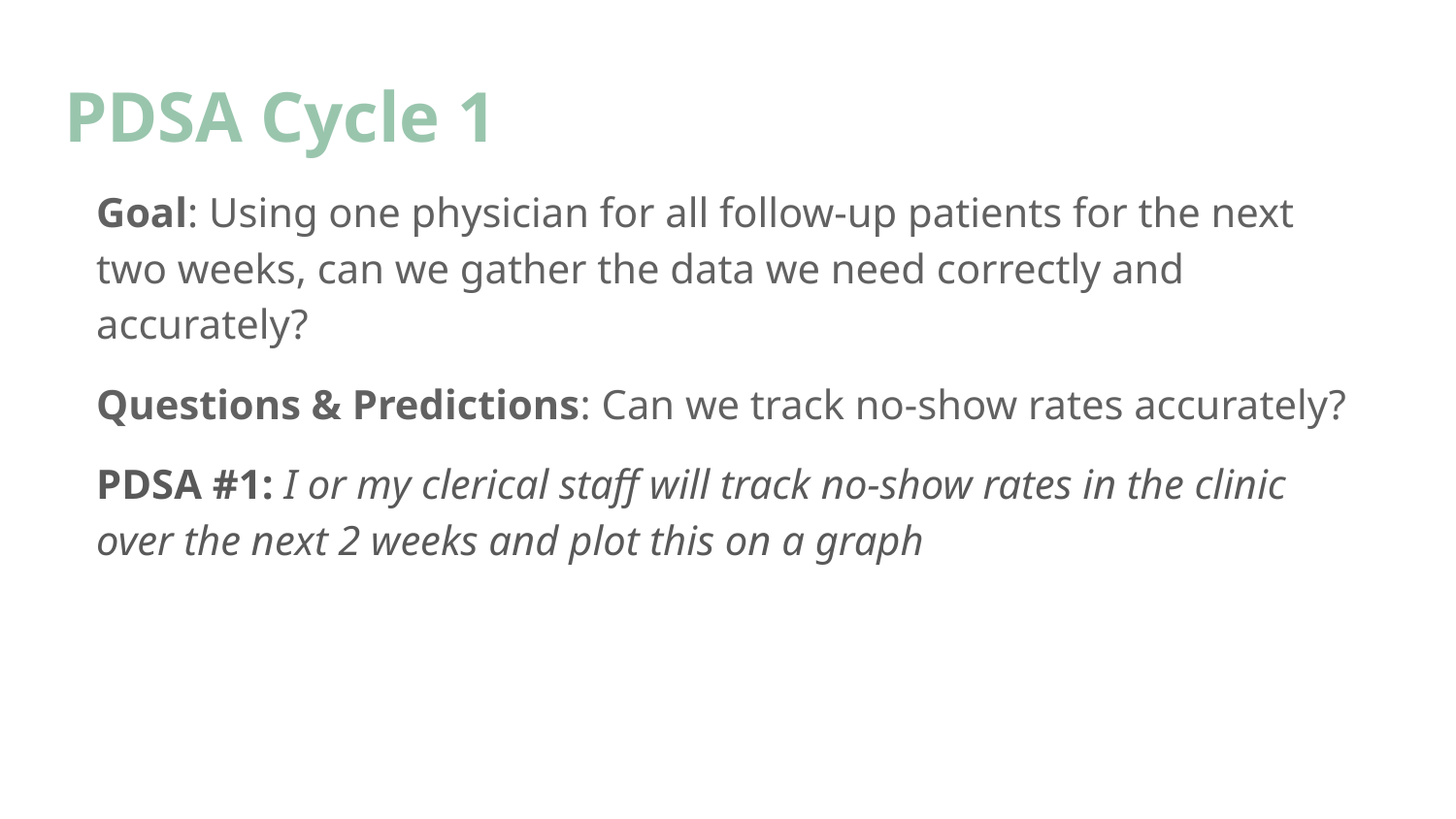

# PDSA Cycle 1
Goal: Using one physician for all follow-up patients for the next two weeks, can we gather the data we need correctly and accurately?
Questions & Predictions: Can we track no-show rates accurately?
PDSA #1: I or my clerical staff will track no-show rates in the clinic over the next 2 weeks and plot this on a graph

## Slide 42
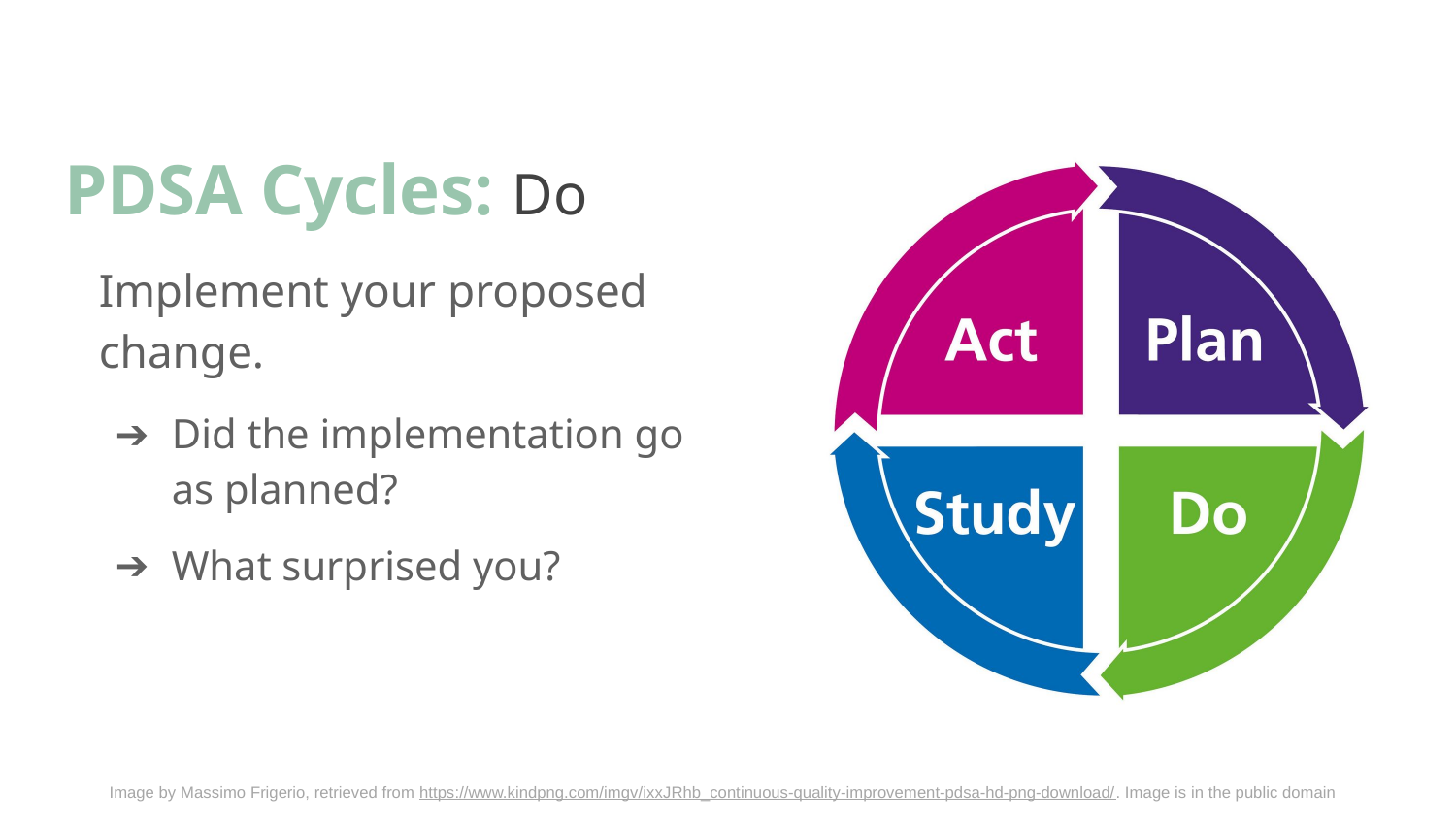

# PDSA Cycles: Do
Implement your proposed change.
Did the implementation go
as planned?
What surprised you?
Image by Massimo Frigerio, retrieved from https://www.kindpng.com/imgv/ixxJRhb_continuous-quality-improvement-pdsa-hd-png-download/. Image is in the public domain

## Slide 43
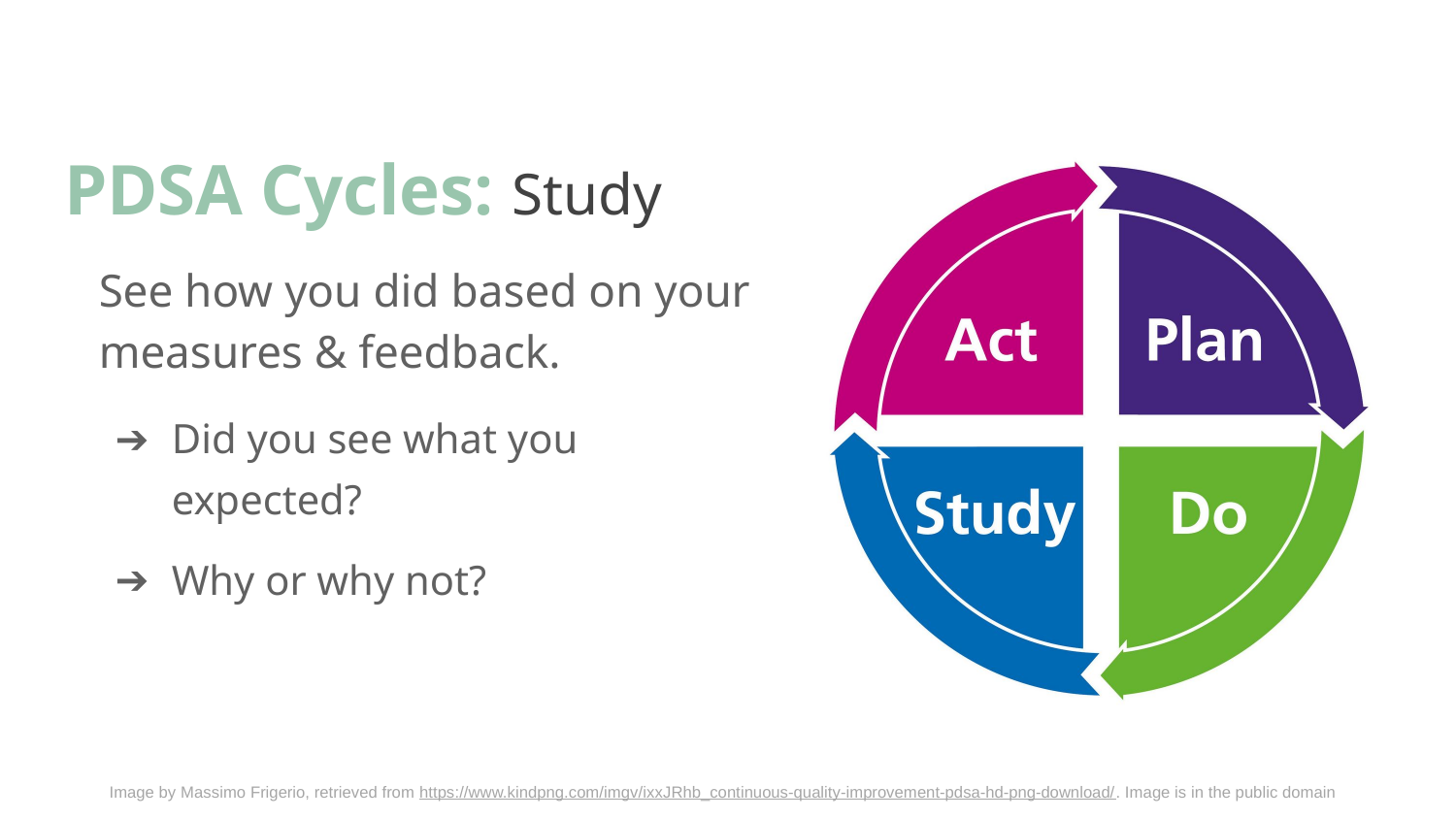

# PDSA Cycles: Study
See how you did based on your measures & feedback.
Did you see what you expected?
Why or why not?
Image by Massimo Frigerio, retrieved from https://www.kindpng.com/imgv/ixxJRhb_continuous-quality-improvement-pdsa-hd-png-download/. Image is in the public domain

## Slide 44
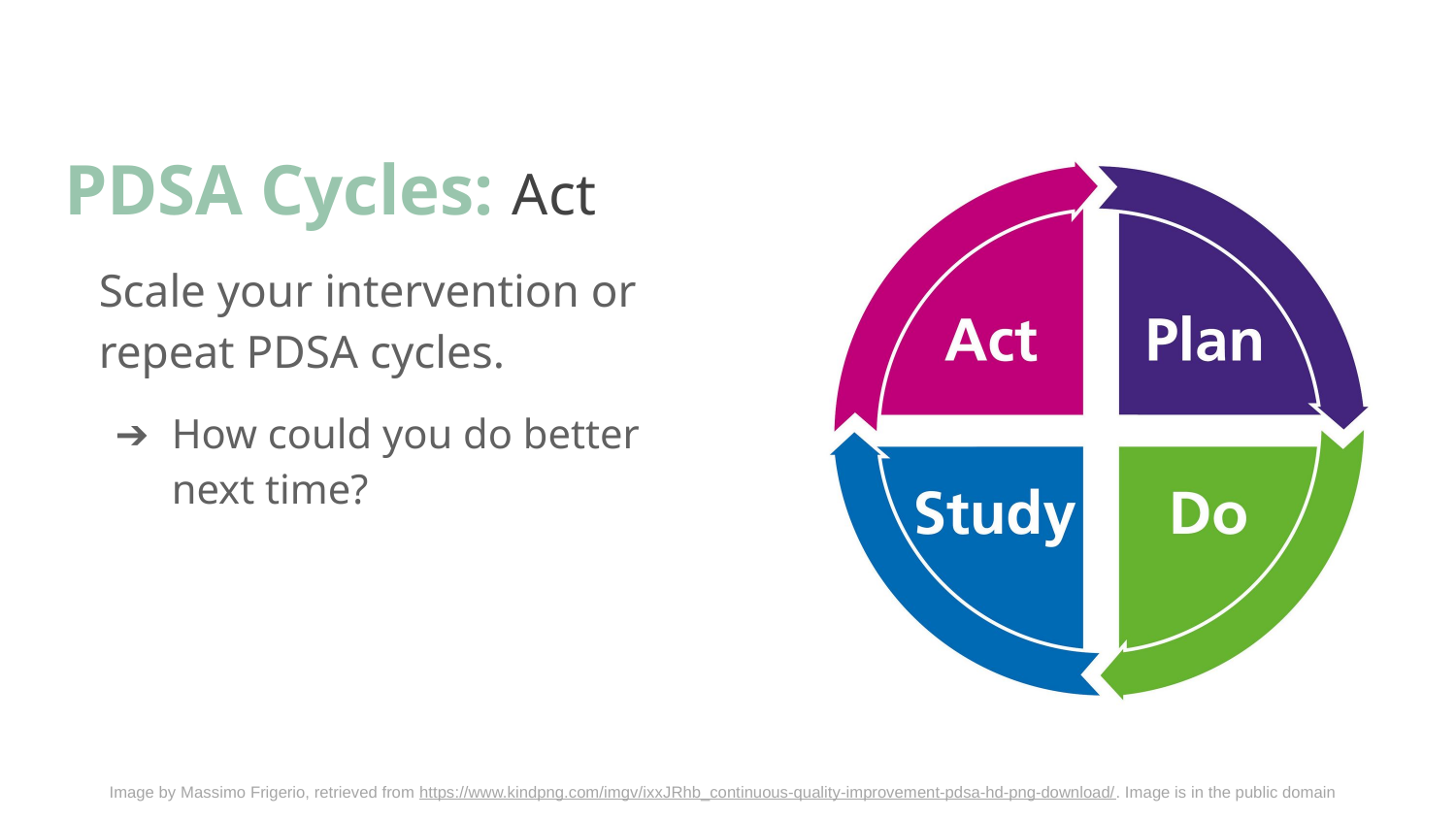

# PDSA Cycles: Act
Scale your intervention or repeat PDSA cycles.
How could you do better
next time?
Image by Massimo Frigerio, retrieved from https://www.kindpng.com/imgv/ixxJRhb_continuous-quality-improvement-pdsa-hd-png-download/. Image is in the public domain

## Slide 45
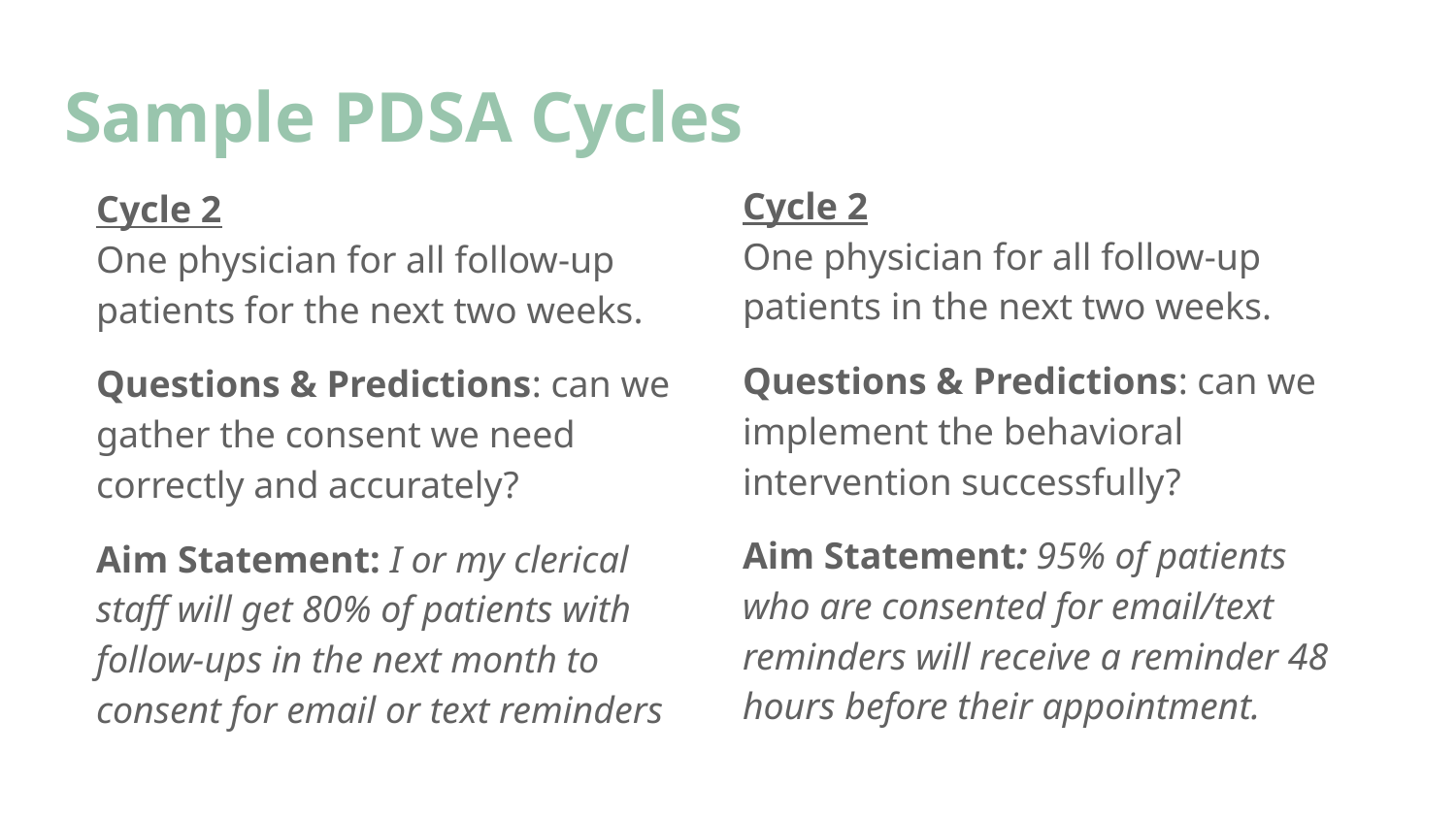

# Sample PDSA Cycles
Cycle 2
One physician for all follow-up patients in the next two weeks.
Questions & Predictions: can we implement the behavioral intervention successfully?
Aim Statement: 95% of patients who are consented for email/text reminders will receive a reminder 48 hours before their appointment.
Cycle 2
One physician for all follow-up patients for the next two weeks.
Questions & Predictions: can we gather the consent we need correctly and accurately?
Aim Statement: I or my clerical staff will get 80% of patients with follow-ups in the next month to consent for email or text reminders

## Slide 46
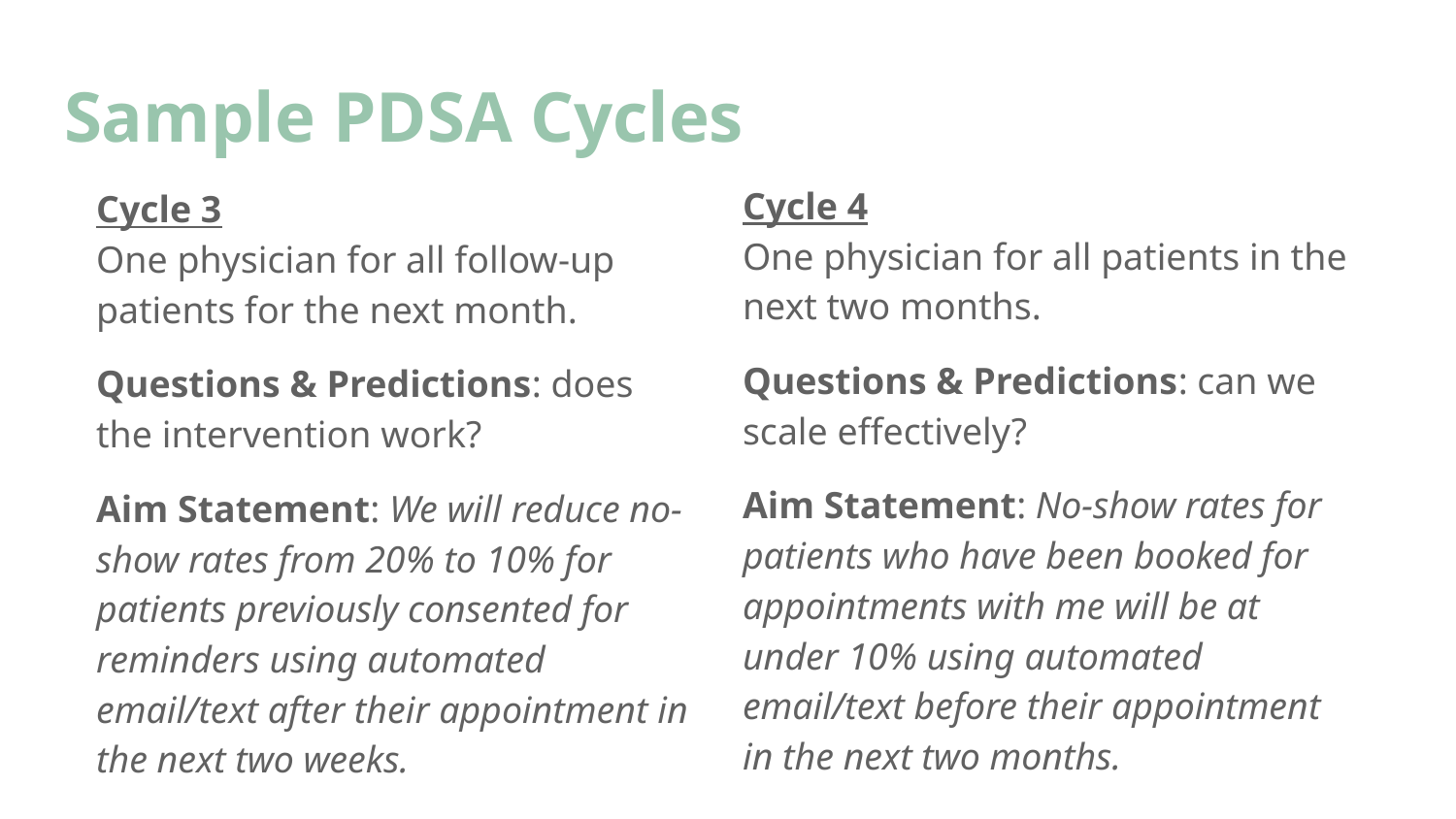

# Sample PDSA Cycles
Cycle 4
One physician for all patients in the next two months.
Questions & Predictions: can we scale effectively?
Aim Statement: No-show rates for patients who have been booked for appointments with me will be at under 10% using automated email/text before their appointment in the next two months.
Cycle 3
One physician for all follow-up patients for the next month.
Questions & Predictions: does the intervention work?
Aim Statement: We will reduce no-show rates from 20% to 10% for patients previously consented for reminders using automated email/text after their appointment in the next two weeks.

## Slide 47
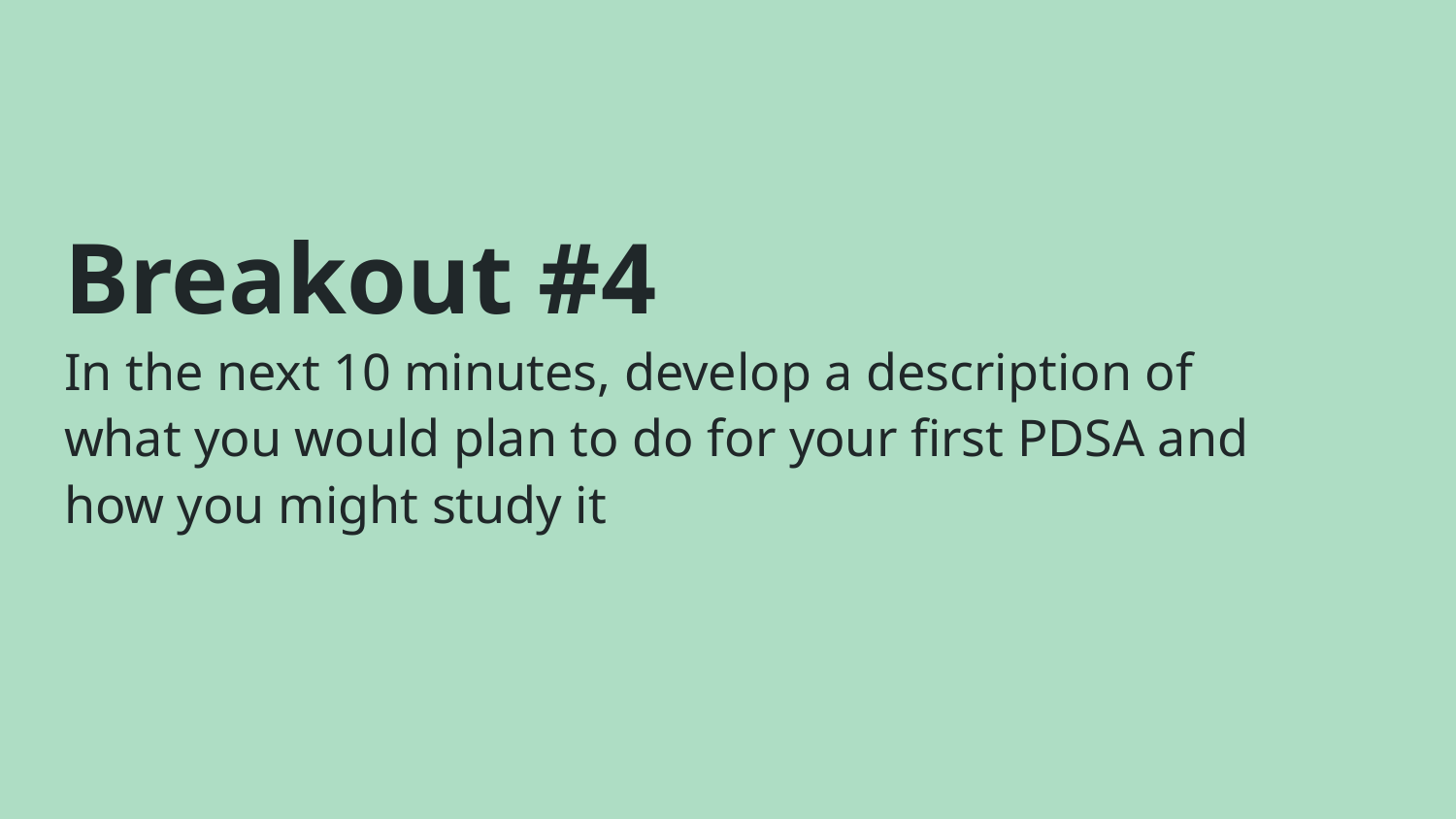

# Breakout #4
In the next 10 minutes, develop a description of what you would plan to do for your first PDSA and how you might study it

## Slide 48
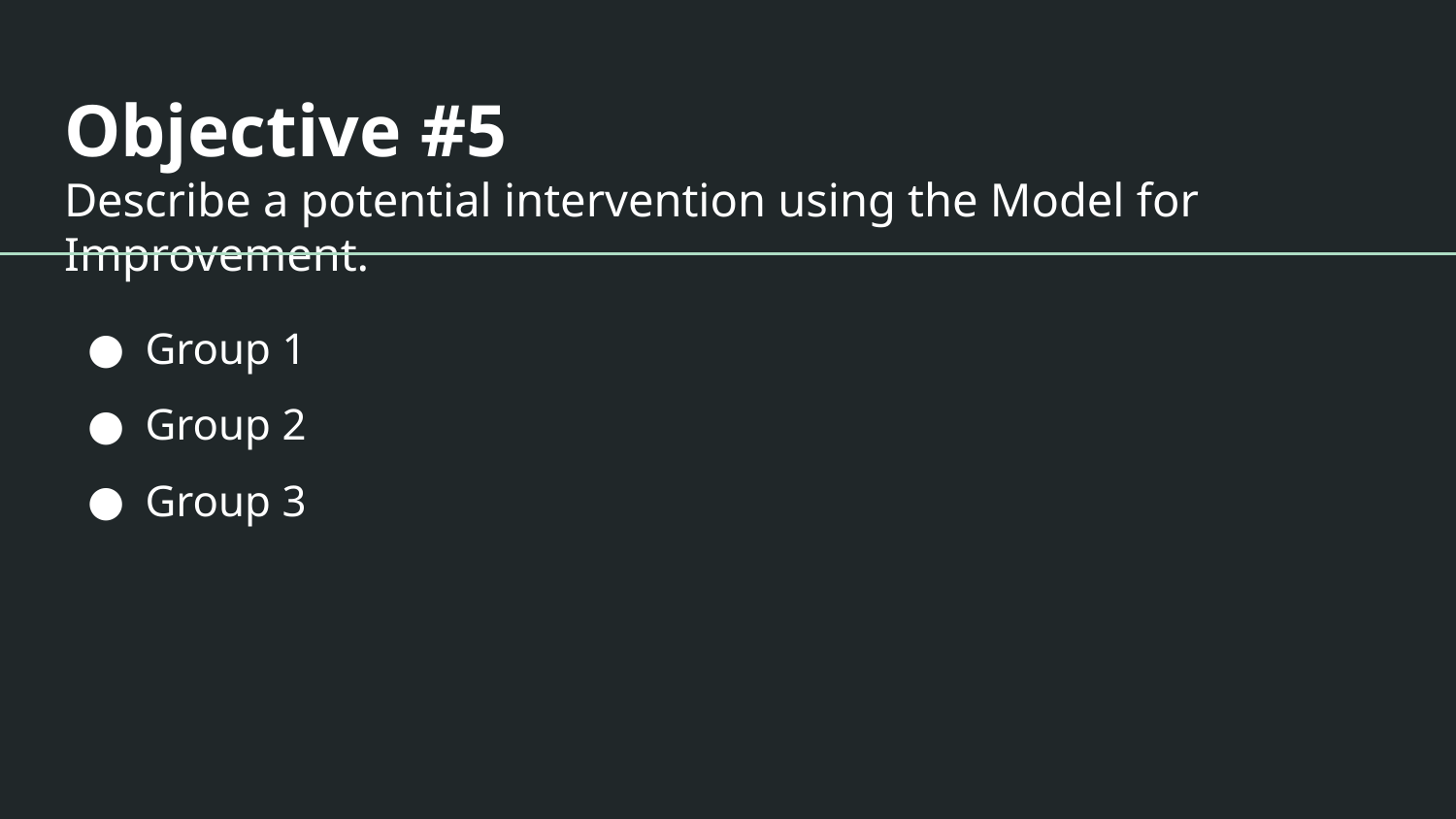

Objective #5
Describe a potential intervention using the Model for Improvement.
Group 1
Group 2
Group 3

## Slide 49
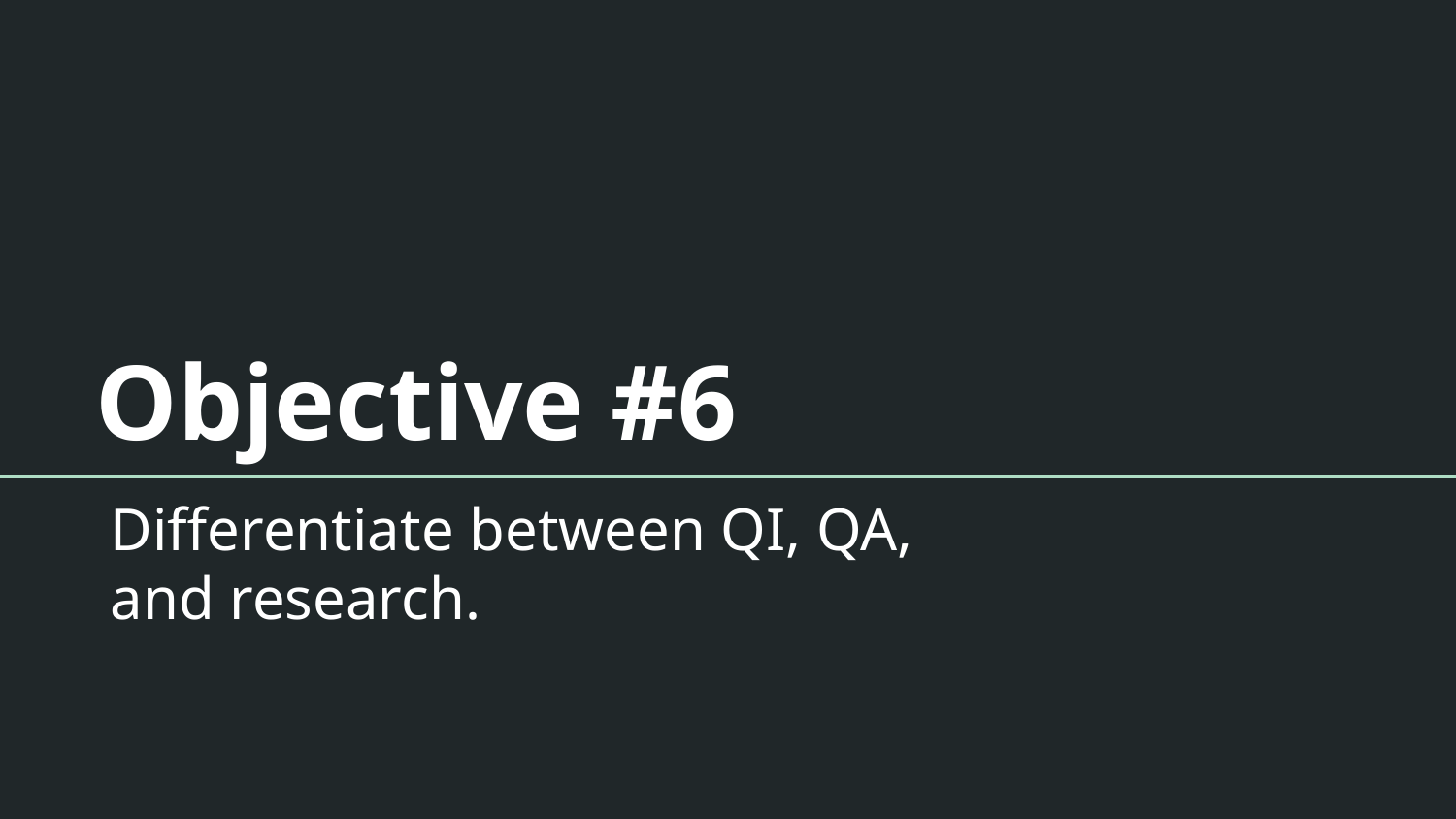

# Objective #6
Differentiate between QI, QA,
and research.

## Slide 50
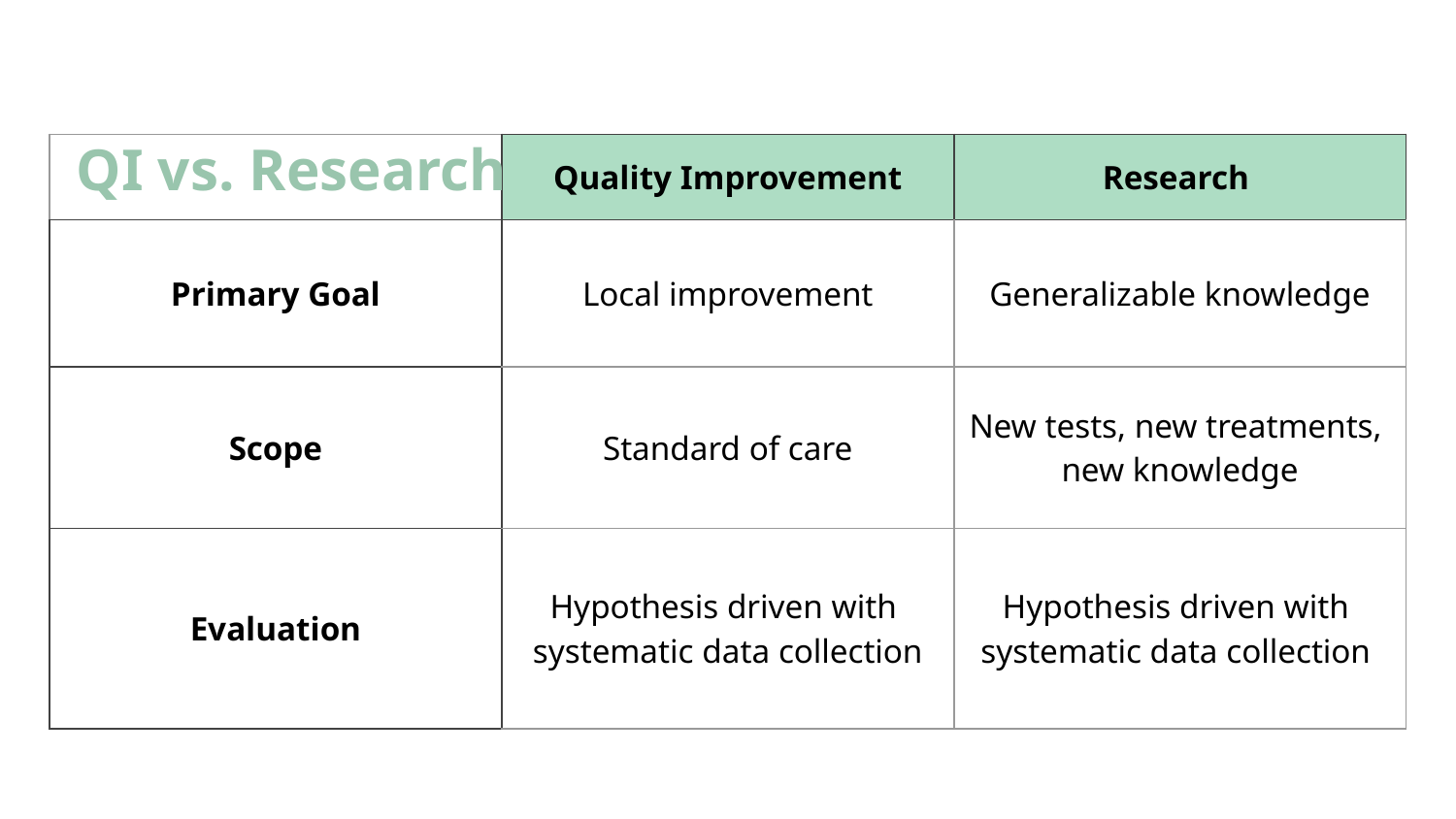

# QI vs. Research
| | Quality Improvement | Research |
| --- | --- | --- |
| Primary Goal | Local improvement | Generalizable knowledge |
| Scope | Standard of care | New tests, new treatments, new knowledge |
| Evaluation | Hypothesis driven with systematic data collection | Hypothesis driven with systematic data collection |

## Slide 51
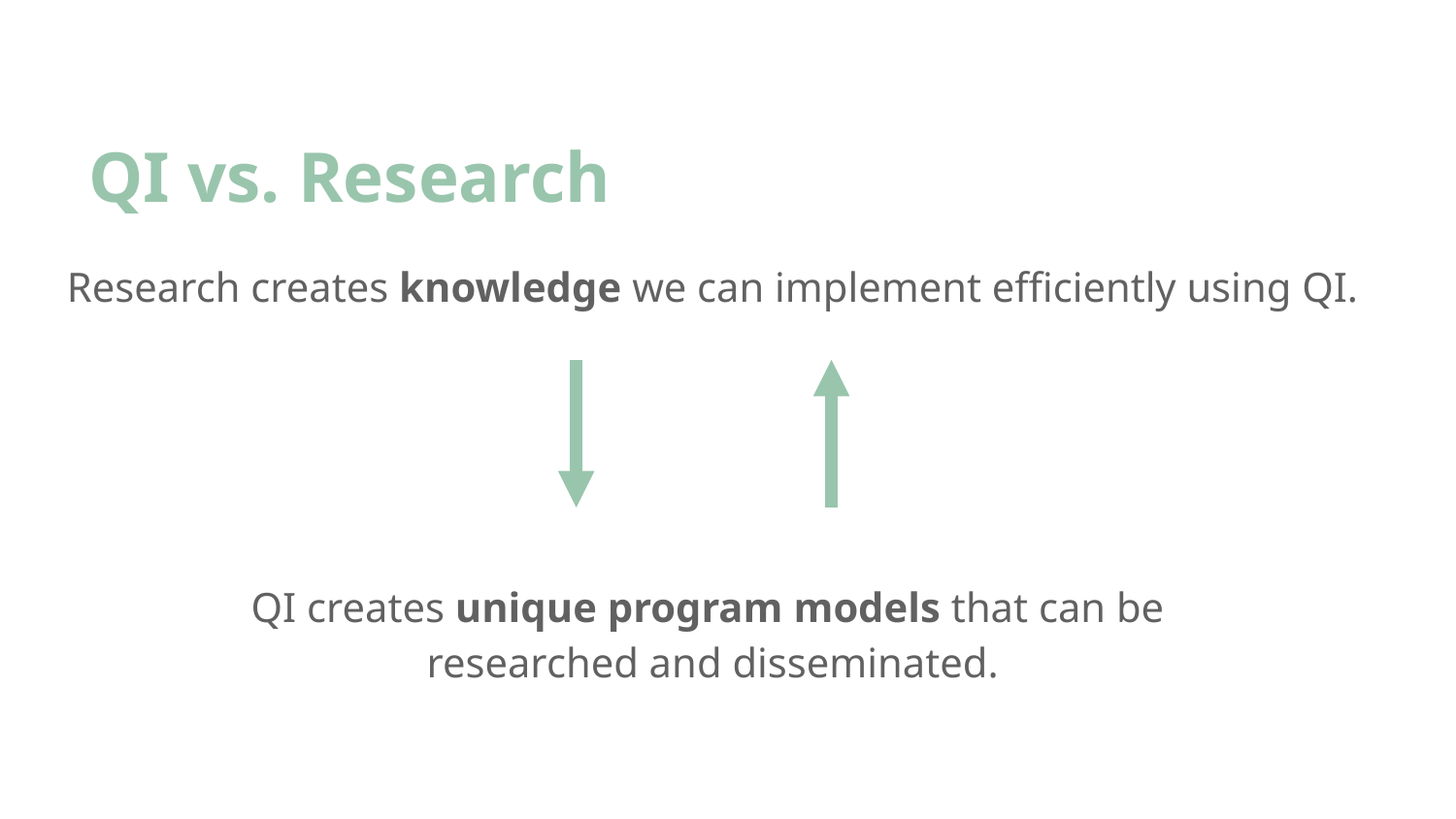

# QI vs. Research
Research creates knowledge we can implement efficiently using QI.
QI creates unique program models that can be
researched and disseminated.

## Slide 52
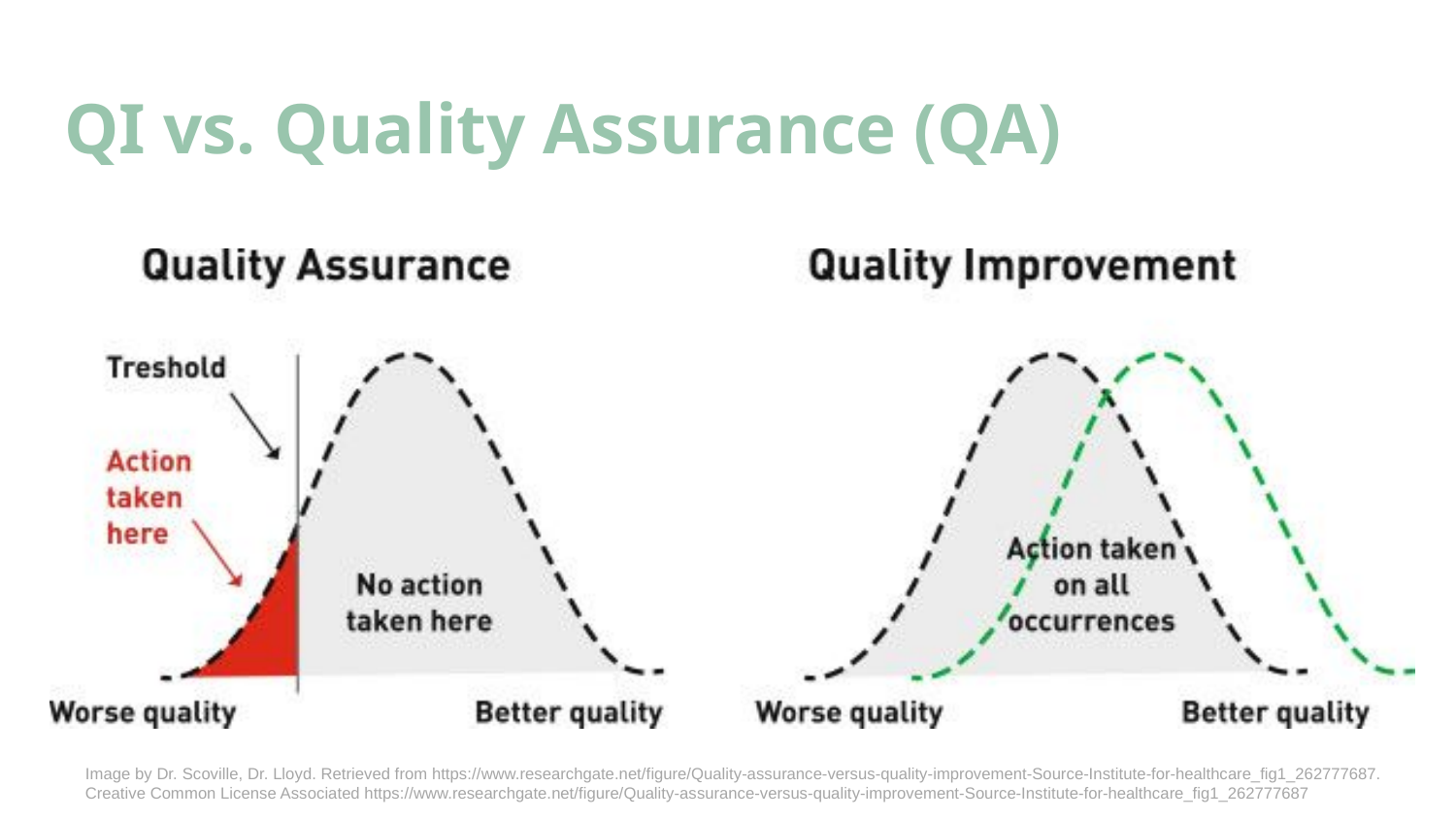

# QI vs. Quality Assurance (QA)
Image by Dr. Scoville, Dr. Lloyd. Retrieved from https://www.researchgate.net/figure/Quality-assurance-versus-quality-improvement-Source-Institute-for-healthcare_fig1_262777687. Creative Common License Associated https://www.researchgate.net/figure/Quality-assurance-versus-quality-improvement-Source-Institute-for-healthcare_fig1_262777687

## Slide 53
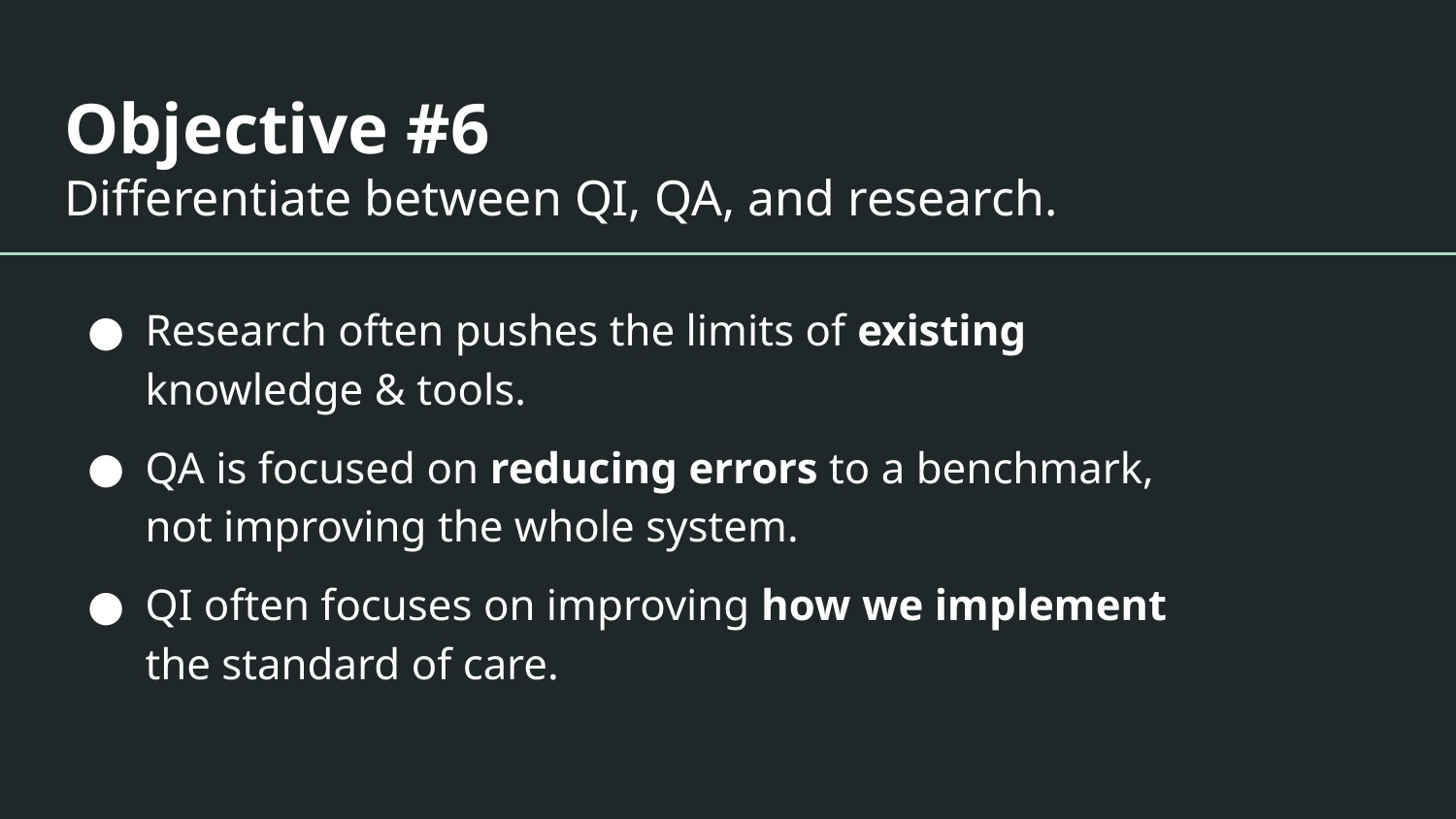

Objective #6
Differentiate between QI, QA, and research.
Research often pushes the limits of existing
knowledge & tools.
QA is focused on reducing errors to a benchmark,
not improving the whole system.
QI often focuses on improving how we implement
the standard of care.

## Slide 54
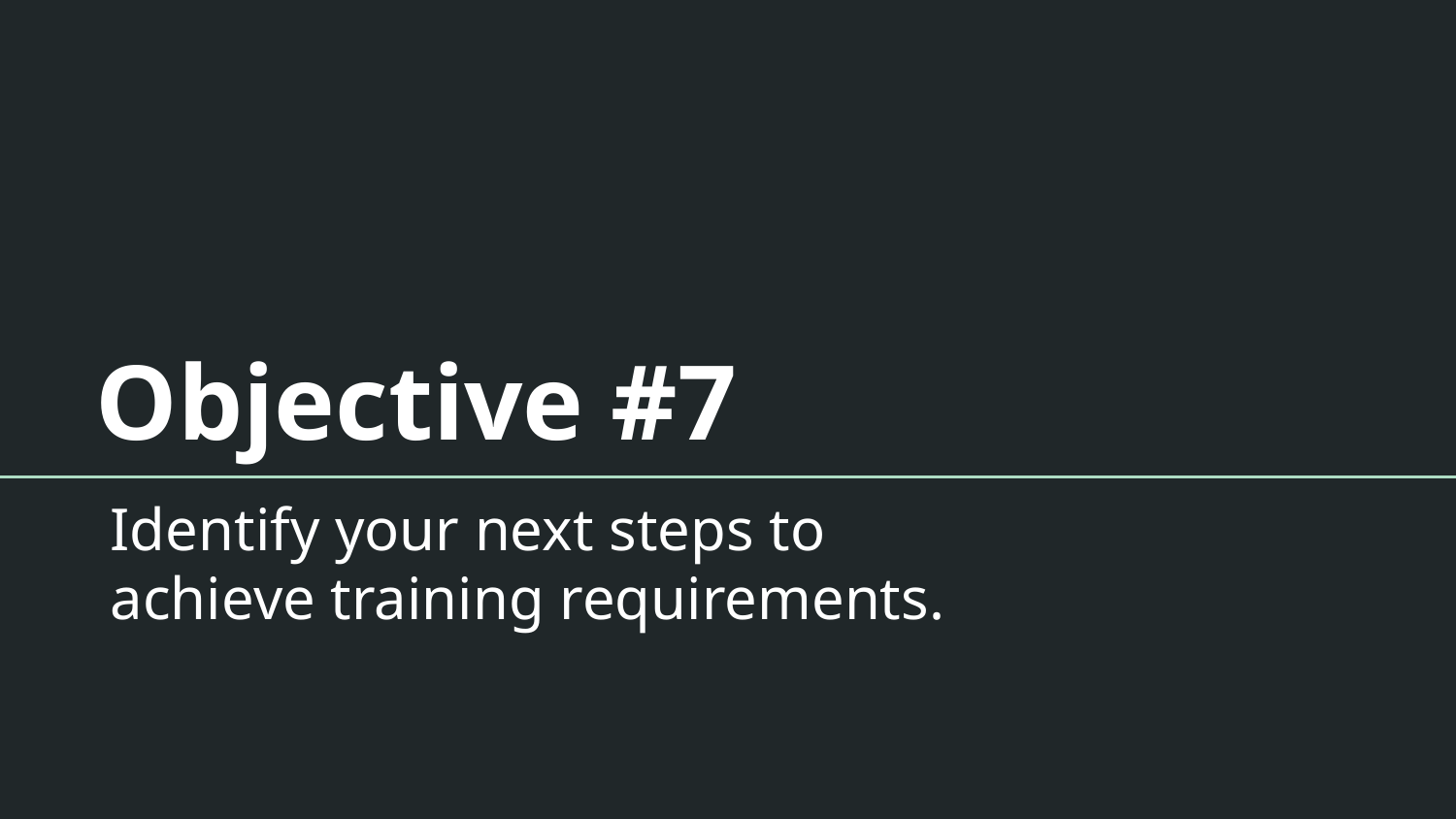

# Objective #7
Identify your next steps to achieve training requirements.

## Slide 55
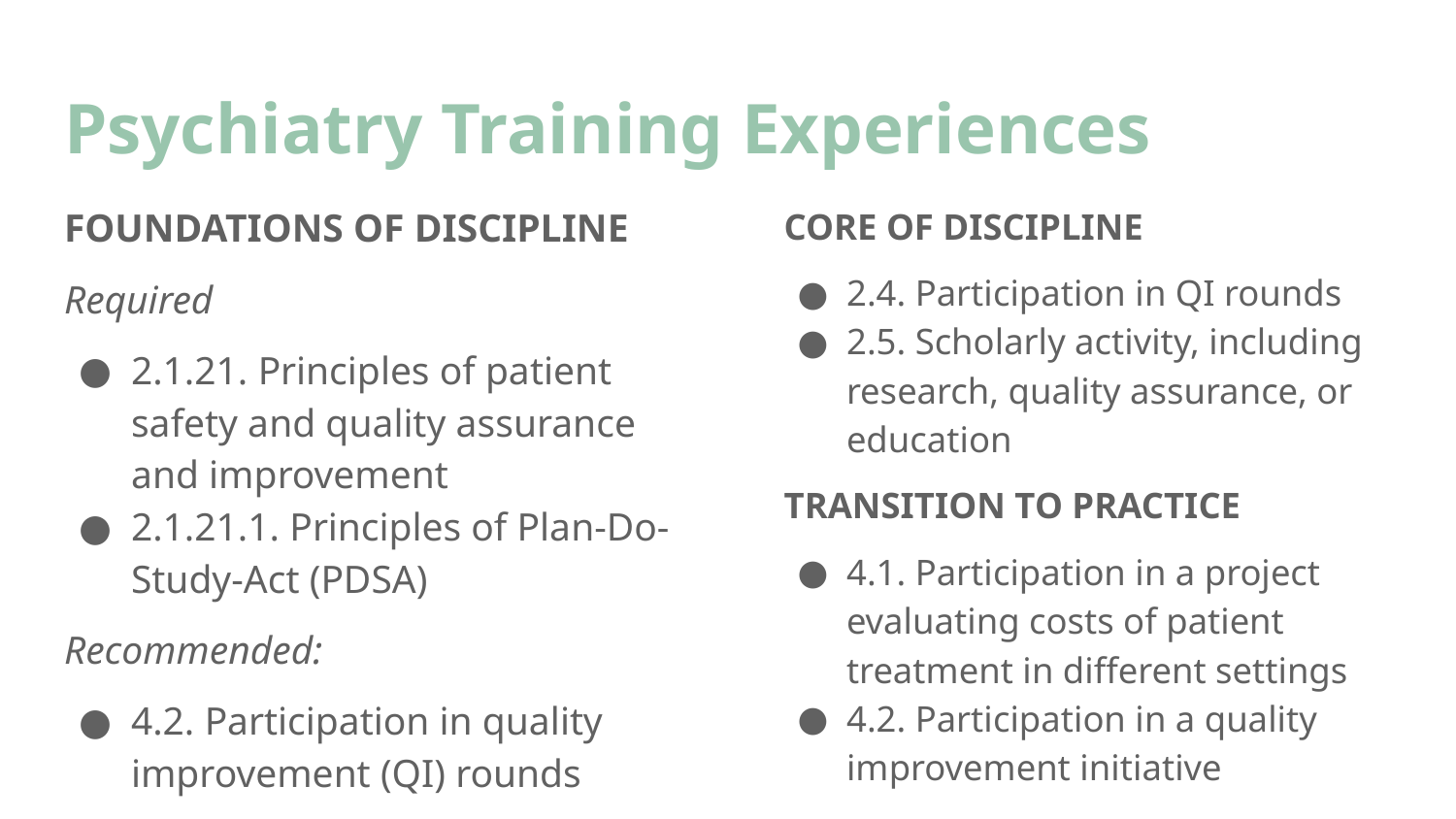

# Psychiatry Training Experiences
FOUNDATIONS OF DISCIPLINE
Required
2.1.21. Principles of patient safety and quality assurance and improvement
2.1.21.1. Principles of Plan-Do-Study-Act (PDSA)
Recommended:
4.2. Participation in quality improvement (QI) rounds
CORE OF DISCIPLINE
2.4. Participation in QI rounds
2.5. Scholarly activity, including research, quality assurance, or education
TRANSITION TO PRACTICE
4.1. Participation in a project evaluating costs of patient treatment in different settings
4.2. Participation in a quality improvement initiative

## Slide 56
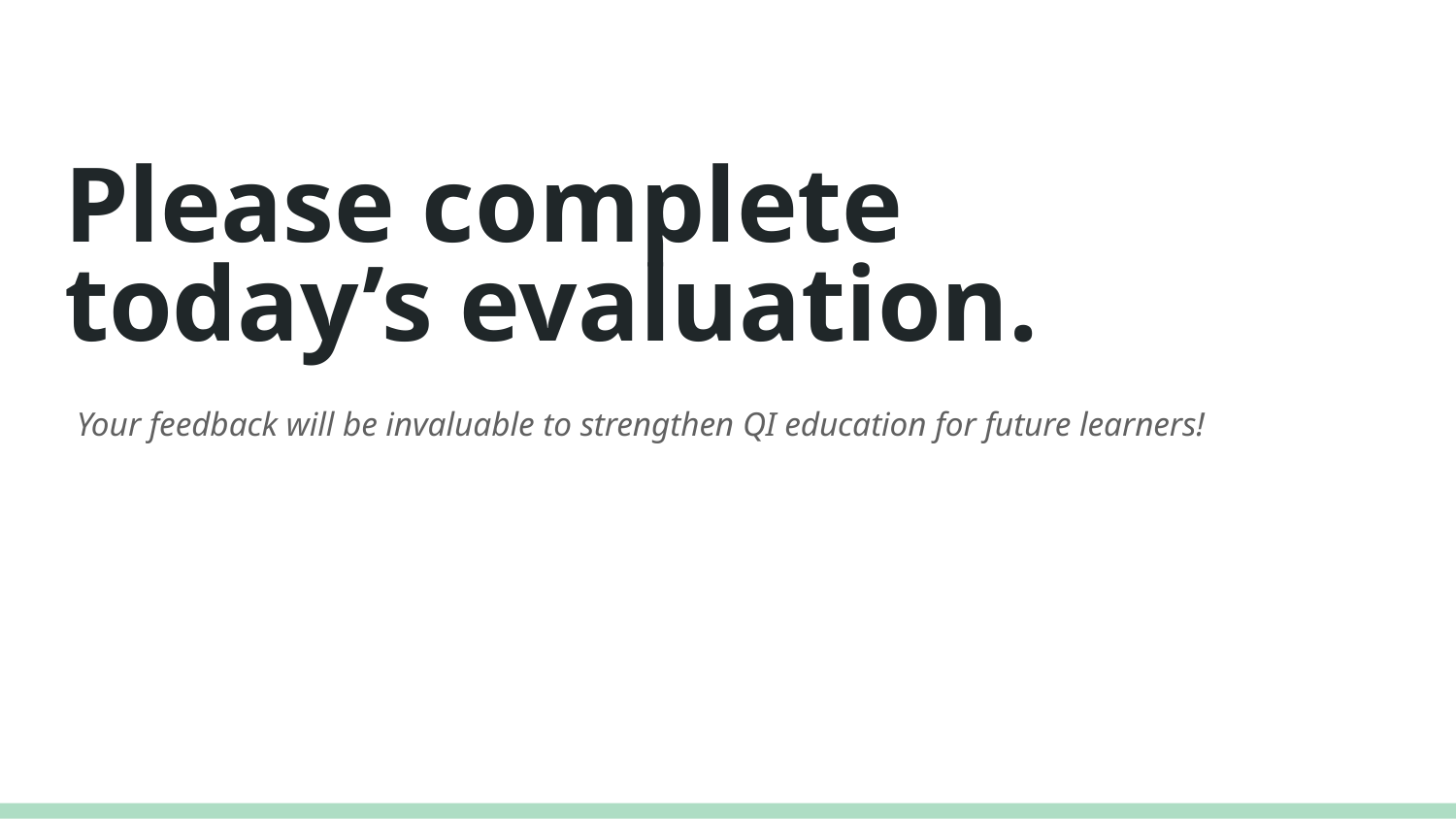

# Please complete today’s evaluation.
Your feedback will be invaluable to strengthen QI education for future learners!
