# Supplementary material for: From Theory to Practice: Development and Evaluation of a Quality Improvement Curriculum for Psychiatry Residents
Source: J Med Educ Curric Dev. 2024 Jan 30;11:23821205241228200. doi: 10.1177/23821205241228200 (PMC10832440; doi:10.1177/23821205241228200)
Supplement: sj-docx-2-mde-10.1177_23821205241228200 - Supplemental material for From Theory to Practice: Development and Evaluation of a Quality Improvement Curriculum for Psychiatry Residents [file sj-docx-2-mde-10.1177_23821205241228200.docx]

Quality Improvement Project Charter

*This handout will provide you with space to complete breakout room activities and will summarize key details discussed during today’s talk.*

**Quality gaps you have noticed during residency:**

**Describe in 2 to 3 sentences the quality gap you hope to improve:**

*Consider which of the quality dimensions it addresses: safety, patient centered, equity, efficiency, effectiveness, timeliness*

**How can you analyze your gap with the FOCUS model?**

1. Find a process (the system containing your gap) to improve

*You have already done this*

2. Organize a team

*Who in your institution really understands the problem, will be impacted by your intervention, or will be able to approve/support the idea through any barriers?*

3. Clarify current knowledge/practices

*Draw a simplified process map of how the process around your quality gap currently works*

4. Understand root causes

*Consider using the “5 whys” or the headers of a fishbone diagram (technology / resources, providers, environment, patients, policy, procedures)*

5. Select the improvement

*Among your ideas for improvement, are there any that are especially low effort but still high impact (i.e. a quick win)*

**What is your overall aim statement?**

*Include a specific goal, specific timeline, and specific target population or location*

**What are your measures?**

- *Outcomes measures: What are we ultimately trying to achieve?*
- *Process measures: Are we doing the right things to get to our goal?*
- *Balancing measures: Are the changes we are making to one part of the system causing problems in other parts of the system?*

**What will you do during your first PDSA cycle?**

*After clarifying your goal, identify what you hope to accomplish that is small in scale / feasible for a first PDSA*
