# Supplementary material for: From Theory to Practice: Development and Evaluation of a Quality Improvement Curriculum for Psychiatry Residents
Source: J Med Educ Curric Dev. 2024 Jan 30;11:23821205241228200. doi: 10.1177/23821205241228200 (PMC10832440; doi:10.1177/23821205241228200)
Supplement: sj-docx-3-mde-10.1177_23821205241228200 - Supplemental material for From Theory to Practice: Development and Evaluation of a Quality Improvement Curriculum for Psychiatry Residents [file sj-docx-3-mde-10.1177_23821205241228200.docx]

**From theory to practice: development and evaluation of a quality improvement curriculum for psychiatry residents**

**STATEMENTS/DISCLOSURES**

- Funding: the authors have no funding sources to disclose.
- Conflicts of Interest: the authors have no conflicts of interest to disclose.

**ACKNOWLEDGEMENTS:**

The authors would like to thank Dr. Lara Hazelton of the Dalhousie University Department of Psychiatry for her feedback on the manuscript, assistance with delivering one workshop, and support of this project.

**RESEARCH DATA:**

Curricular materials have been attached as a supplementary file. Should you have any further questions please contact the authors.
