# Supplementary material for: From Theory to Practice: Development and Evaluation of a Quality Improvement Curriculum for Psychiatry Residents
Source: J Med Educ Curric Dev. 2024 Jan 30;11:23821205241228200. doi: 10.1177/23821205241228200 (PMC10832440; doi:10.1177/23821205241228200)
Supplement: sj-docx-4-mde-10.1177_23821205241228200 - Supplemental material for From Theory to Practice: Development and Evaluation of a Quality Improvement Curriculum for Psychiatry Residents [file sj-docx-4-mde-10.1177_23821205241228200.docx]

| MANUSCRIPT ID: MDE-23-0198MANUSCRIPT TITLE: "From theory to practice: development and evaluation of a quality improvement curriculum for psychiatry residents" | | |
| --- | --- | --- |
| SQUIRE-EDU item | Description in text | Comments |
| **EDU 1.** Indicate that the manuscript concerns efforts to improve health professions education systems and learning | Introduction, paragraph 3 – this is an effort to develop and improve a curriculum using best practices and education theory |  |
| **EDU 2.**Keywords include a focus on education and learning | Keywords below abstract |  |
| **EDU 3.**Description of the nature and significance of the need for change in the local educational system | Methods, paragraph 1. This curriculum was mandatory per national training standards |  |
| **EDU 5.**Identify the guiding theory (learning, change, implementation, or other) and how it aligns with the need for change in the local educational system | Best practices were identified from a recent programme theory published by Brown et al. Many of the practices that were highlighted align with Adult Learning Theory |  |
| **EDU 7a.**Contextual elements for learning (e.g., setting, program, people, resources, social, geopolitical influences) before the intervention(s)  ______________  **EDU 7b:** The interrelationships between the contextual elements and the local educational and healthcare systems before the intervention(s) | Described in methodology paragraph 1 and 3. In short, this is a novel effort for a new curriculum that has not existed previously. The decision of when and how to deliver it was a de novo effort based on education theory in QI.  At this time, there is no significant interrelationship between the contextual elements and the local health system. There may be in the future however. |  |
| **EDU 8a .**Description of the primary interventions and co-interventions (e.g., faculty or tool development) | Outlined under curriculum design within methodology |  |
| **EDU 8b.**Specify how the interprofessional education team (e.g., faculty, staff, patients, and learners) was part of the design of the intervention | The curriculum itself was delivered by a resident and a staff, though this did not hold any relevance to the specific design of the intervention, which was wholly based on existing education theory. |  |
| **EDU 9a.**Approach used to understand the impact of the educational intervention(s) on the learner and beyond, such as impact on patients, families, the community, faculty, educational program, or the healthcare system | BASiC-QI scale was used to assess impact on learners. Iterative improvement was not impelemented from the learners themselves to ensure that subsequent cohorts of residents received the same intervention to increase sample size (otherwise sample size would have been 5). |  |
| **EDU 9b**. Approach to assess the fidelity of and the iterative changes to the planned intervention(s) over time | Not applicable as this is an initial intervention |  |
| **EDU 10.** Quantitative and/or qualitative measures chosen to assess the educational processes and outcomes on learners, faculty, educational programs, patients, families, healthcare systems, or communities | QIKAT-R for knowledge, BASiC-QI for beliefs and attitudes. |  |
| **EDU 12.** Approaches to address vulnerability of learner participants | Knowledge of which learners participated in the study was fully blinded to the author |  |
| **EDU 13a.** For each educational intervention and co-intervention, provide details about iterative modifications based on the assessment of the learning | Not applicable – no modifications |  |
| **EDU 14.** Connect the findings to the guiding theory (learning, change, implementation, other) used to direct the change in the local educational system | The approach used was linked to the guiding programme theory in both the methodology section and the discussion (to highlight future steps) |  |
| **EDU 15c.** Include the impact of the intervention(s) on learners, faculty, educational program, patients, families, healthcare systems, or communities | Using BASiC-QI, learners did appear to increase their appreciation of QI based on the curriculum. There was no system level measurement otherwise of impacts. |  |
| **EDU 17b.** Scalability of the work to other learners and contexts | Discussed thoroughly in discussion – this methodology used in the paper can be easily replicated by other educators |  |
| **EDU 17d.** Lessons learned for clinical practice, education, and policy | This paper is the first example of how a programme theory of QI can be used to rapidly develop and iterate a QI curriculum. This approach could be adopted by educators to assess and approve their own curricula. |  |
